# Installation of internal electric fields by non-redox active cations in transition metal complexes†
†Electronic supplementary information (ESI) available: Experimental methods, synthetic procedures, ^1^H NMR spectra, electronic absorption spectra, cyclic voltammetry, infrared spectra, crystallographic data, computational data and coordinates. CCDC 1922171 and 1922172. For ESI and crystallographic data in CIF or other electronic format see DOI: 10.1039/c9sc02870f


**DOI:** 10.1039/c9sc02870f

**Published:** 2019-09-09

**Authors:** Kevin Kang, Jack Fuller, Alexander H. Reath, Joseph W. Ziller, Anastassia N. Alexandrova, Jenny Y. Yang

**Affiliations:** a Department of Chemistry , University of California , Irvine 92697 , USA . Email: j.yang@uci.edu; b Department of Chemistry and Biochemistry , University of California, Los Angeles , Los Angeles , CA 90095 , USA . Email: ana@chem.ucla.edu; c California NanoSystems Institute , Los Angeles , CA 90095 , USA

## Abstract

Local electric fields contribute to the high selectivity and catalytic activity in enzyme active sites and confined reaction centers in zeolites by modifying the relative energy of transition states, intermediates and/or products. Proximal charged functionalities can generate equivalent internal electric fields in molecular systems but the magnitude of their effect and impact on electronic structure has been minimally explored. To generate quantitative insight into installing internal fields in synthetic systems, we report an experimental and computational study using transition metal (M_1_) Schiff base complexes functionalized with a crown ether unit containing a mono- or dicationic alkali or alkaline earth metal ion (M_2_). The synthesis and characterization of the complexes M_1_ = Ni(ii) and M_2_ = Na^+^ or Ba^2+^ are reported. The electronic absorption spectra and density functional theory (DFT) calculations establish that the cations generate a robust electric field at the metal, which stabilizes the Ni-based molecular orbitals without significantly changing their relative energies. The stabilization is also reflected in the experimental Ni(ii/i) reduction potentials, which are shifted 0.12 V and 0.34 V positive for M_2_ = Na^+^ and Ba^2+^, respectively, compared to a complex lacking a proximal cation. To compare with the cationic Ni complexes, we also synthesized a series of Ni(salen) complexes modified in the 5′ position with electron-donating and -withdrawing functionalities (–CF_3_, –Cl, –H, –*t*Bu, and –OCH_3_). Data from this series of compounds provides further evidence that the reduction potential shifts observed in the cationic complexes are not due to inductive ligand effects. DFT studies were also performed on the previously reported monocationic and dicatonic Fe(ii)(CH_3_CN) and Fe(iii)Cl analogues of this system to analyze the impact of an anionic chloride on the electrostatic potential and electronic structure of the Fe site.

## Abstract 2

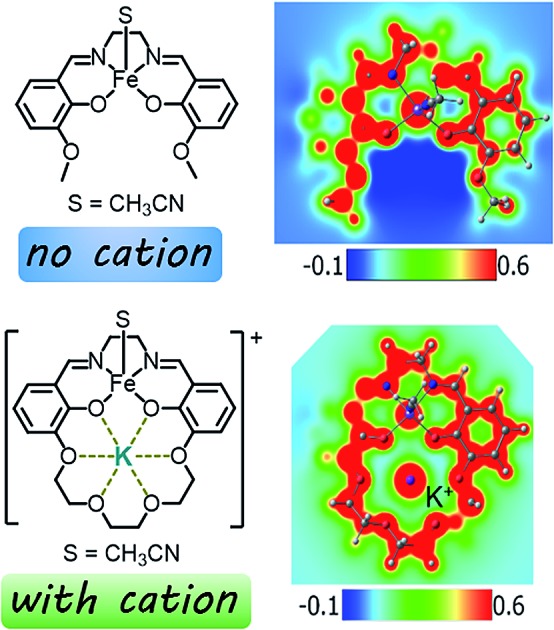
Experimental and computational study quantifying internal electric fields in synthetic systems using transition metal Schiff base complexes functionalized with a crown ether unit containing a mono- or dicationic alkali or alkaline earth metal ion.

## Introduction

Directional electric fields can play a critical role in directing the rate and selectivity of chemical catalysis by manipulating activation energies and the relative energies of catalytic intermediates.^1^,^2^ Electrostatic fields are believed to be key contributors to the accelerated reactivity or selectivity observed in a wide-range of environments, from enzymatic active sites^3^,^4^ to zeolite pores.^5^,^6^

Quantum mechanical studies have described harnessing directional electric fields as a ‘smart reagent’ for mediating chemical reactivity.^7^ Recent experimental studies have illustrated the utility of applied (or external) electrostatic fields in mediating reactivity. For example, Kanan *et al.* demonstrated the use of electric fields generated at electrode surfaces to control metal oxide catalyzed epoxide rearrangement^8^ and a carbene reaction.^9^ Additionally, electric fields generated between an STM tip and surface can accelerate localized isomerization reactions,^10^ a single molecule Diels–Alder reaction,^11^ or cleavage of a C–O bond.^12^ Localized electric fields from electrode nanostructures also plays a significant role in electrocatalysis, with a prominent case revealing significantly improved selectivity for CO_2_ reduction to hydrocarbons.^13^ These experiments demonstrate the power of electric fields to accelerate or induce desirable reactivity. However, the magnitude of an external electric field generated at an electrode diminishes rapidly, limiting its effect. Similarly, electric field generation at an STM tip is limited to localized or single molecule reactivity. Additionally, higher external fields can result in dielectric breakdown or deleterious faradaic processes.

In contrast, installation of an internal electric field in a molecular system can provide a predictable, directional, and consistent electric field at each active site. Electric fields generated by internal dipoles have been increasingly cited in selective or increased reactivity for organic synthesis.^14^–^30^ Kanan *et al.* have demonstrated that the field generated by ion pairing modifies the regioselectivity of Au(i)-catalyzed hydroarylation of 3-substituted phenyl propargyl ethers.^31^ Coote *et al.* have also reported using pH switchable fields that rely on protonation–deprotonation to vary radical stability.^32^

**Chart 1:**
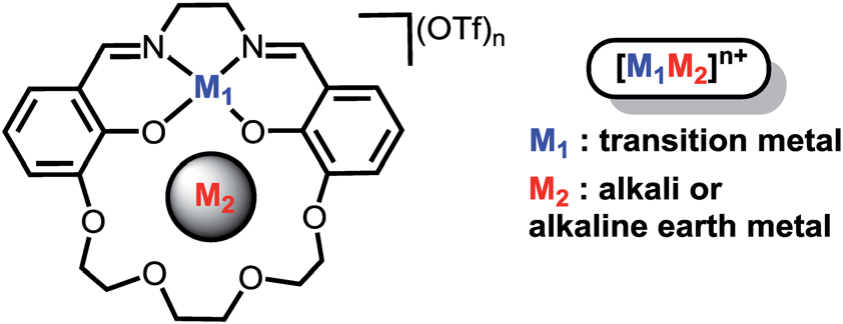


Our efforts to generate controllable and directional internal electric fields have focused on installation of non-redox active cations proximal to redox-active reaction sites. We first reported the cobalt derivatives of the salen-crown complex shown in Chart 1. The crown moiety appended to the four-coordinate Schiff-base ligand can accommodate a large range of cationic metal centers (M_2_). Alkali and alkaline earth metals were utilized because they are known to generate significant electric fields in enzymes^33^,^34^ and zeolite cavities.^35^ A significant shift in the Co(ii/i) redox potential was observed upon installation of the redox-inactive cation. Electronic absorption spectroscopy indicated the ligand field is not significantly perturbed by M_2_. Instead, we suggested the shifts in redox potential were likely due to the electrostatic field generated by M_2_, which was supported by modelling M_2_ as a point charge.^36^ A subsequent study on the iron analogues of the ligand in Chart 1 found the magnitude of the Fe(iii/ii) redox potential also had a strong dependence on both M_2_ and whether a counterion was coordinated to the Fe.^37^ However, our initial studies lacked definitive evidence for the magnitude of the electric field, its impact on the electronic structure of the redox-active center, and the effect of inner-sphere anions. These details are critical to effectively utilize cations to generate directional electric fields to optimize reaction rates or selectivity.

**Scheme 1:**
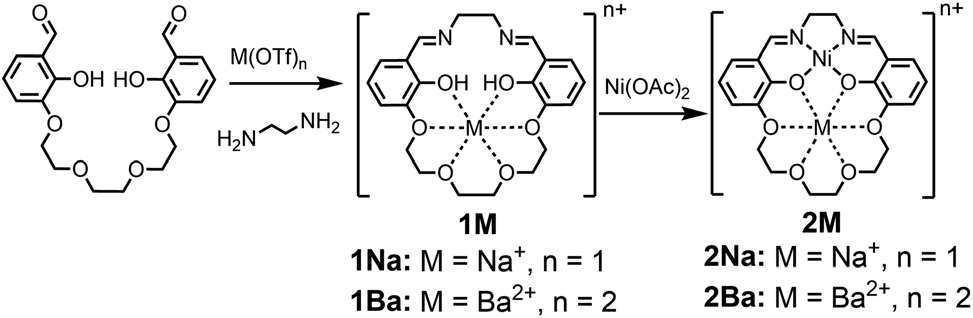


In order to investigate the electric field effect in more detail, we have synthesized the Ni(ii) (M_2_) analogue of the ligand with Na^+^ and Ba^2+^ cations as M_2_ (Scheme 1). As electric fields scale with charge, we would expect dications to have a larger effect on the magnitude than monocations. The salen framework easily accommodates a square-planar closed-shell Ni(ii) coordination environment. In this study, we detail the synthesis and spectroscopic properties of Ni(ii) complexes with Na^+^ or Ba^2+^ incorporated in the crown with computational methods to more fully understand the changes in redox potential and electronic properties. Computational methods are also applied to the previously reported iron complexes in this framework to understand the impact of anion coordination on the electronic structure and redox potential. Additionally, we have synthesized a series of Ni(salen) complexes with varying electron-donating and -withdrawing functionalities in the 5′ position. We find that the relationship between the spectroscopic properties and reduction potential contrast with that of **2M**, indicating minimal inductive ligand effects with the latter. Taken together, this study provides a detailed and quantitative examination of how positioned cations can be utilized in synthetic systems to install electric field of fixed magnitude and direction.

## Results and discussion

### Synthesis

The **1M** Schiff base ligands (Scheme 1) were synthesized using a modified literature procedure.^38^ A dialdehyde with an ether chain was templated with an alkali or alkaline earth metal triflate salt, which subsequently underwent macrocyclization *via* condensation with one equivalent of ethylene diamine. **1Na(OTf)** and **1Ba(OTf)_2_** were synthesized using the corresponding metal triflate salts. They were purified *via* recrystallization by layering diethyl ether on a methanol solution of the crude product (Scheme 1, M = Na^+^, Ba^2+^).

**1M** (M = Na^+^, Ba^2+^) ligand and an equivalent of Ni(OAc)_2_ were refluxed in methanol to synthesize the heterobimetallic **2M** Ni(ii) complexes (Scheme 1). Synthesis was followed by removal of methanol and acetic acid *in vacuo*. The heterobimetallic complexes were purified and recrystallized by vapor diffusion of diethyl ether into the methanol solutions of each compound. The compound identities were confirmed using high resolution mass spectrometry and single crystal X-ray diffraction analysis. Purities were determined by elemental analysis. Ni(3′-OCH_3_-salen) (3′-OCH_3_-salen = *N*,*N*′-bis(3-methoxysalicylidene)-1,2-diaminoethane) was synthesized according to a literature procedure^39^ to compare with the **2M** complexes.

The compounds were characterized by ^1^H NMR spectroscopy, shown in Fig. S2 and S3.† Compared to Ni(3′-OCH_3_-salen), each **2M** complex exhibited a downfield shift in the imine proton signal with a greater shift correlating with higher cationic charge. However, the imine proton in **1M** complexes was shifted even farther downfield than in the corresponding **2M** complexes.

The 5′-R salen ligands (R = CF_3_, Cl, *t*Bu, OCH_3_) were synthesized using a modified literature procedure.^39^ Two equivalents of 2-hydroxy-5-R-benzaldehyde were dissolved in methanol and condensed with one equivalent of ethylenediamine to form the substituted salen ligand. Each 5′-R-salen ligand and an equivalent of Ni(OAc)_2_ were refluxed in methanol to form the Ni(5′-R-salen) complexes. The products were isolated by removal of methanol and acetic acid under reduced pressure. ^1^H NMR spectra for each Ni complex is shown in Fig. S4–S8.†

### Structural studies

**Fig. 1:**
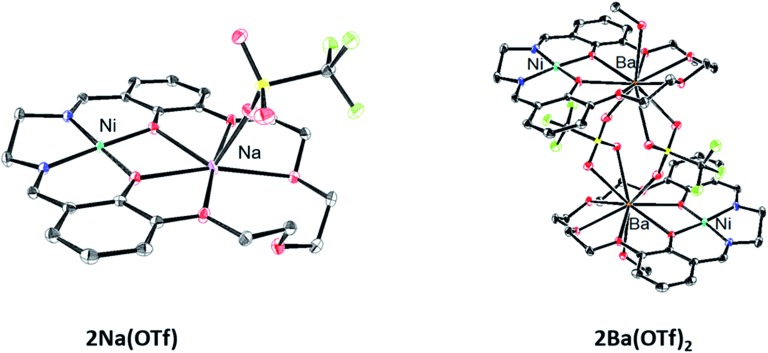
Solid-state structures of **2Na(OTf)** and **2Ba(OTf)_2_**. Thermal ellipsoids are drawn to 50% probability. Hydrogen atoms, outer-sphere anions, and solvent molecules have been omitted for clarity.

Single crystals for X-ray diffraction were grown by diffusion of diethyl ether into methanol solutions of the **2M** triflate salts; the solid-state structures are shown in Fig. 1. In each heterobimetallic complex, the triflate counteranions are bound to the M_2_ cation. In **2Na(OTf)**, the Na^+^ ion only appears bound to 5 of the 6 available oxygen donors. In **2Ba(OTf)_2_**, two triflate anions are coordinated to the Ba^2+^ and form a symmetric bridge to an adjacent **2Ba(OTf)_2_** complex. The mass spectrum does not contain peaks consistent with a dimer, suggesting that the dimeric species only exists in the solid-state form. A methanol molecule is also coordinated to each Ba^2+^ cation.

Each complex contains a minimally distorted square planar nickel ion with the M_2_ cation inhabiting the crown ether pocket. The *τ*_4_ values describing the coordination around the Ni(ii) ions are listed in Table 1 and confirm similar coordination environments between the compounds.^40^ The distances between the Ni(ii) ion and M_2_ cations are also listed in Table 1.

**Table 1:** Summary of structural, spectroscopic, and electrochemical data for Ni(3′-OCH_3_-salen) and **2M** complexes. Electrochemical and electronic absorption spectroscopy were taken in dimethylformamide

Complex	*E*_1/2_ Ni(ii/i)^*a*^, V	Δ*E*^*b*^, V	Ni···M, Å	Ni(ii) ion, *τ*_4_^*c*^	*ν*(C=N), cm^–1^	*λ*(d → d), nm (*ε*, M^–1^ cm^–1^)	Calc. *λ*(d → d), nm	*λ*(π → π*)^*e*^, nm (*ε*, M^–1^ cm^–1^)	Calc. *λ* (π → π*), nm	*λ*(MLCT)^*e*^, nm (*ε*, M^–1^ cm^–1^)	Calc. *λ*(MLCT), nm
Ni(3′-OCH_3_-salen)	–2.11	—	—	0.0152^*d*^	1545	547 (111)	563	349 (8033)	345	415 (5019)	390
**2Na(OTf)**	–1.99	0.12	3.4374(6)	0.0217	1553	552 (113)	565	345 (9800)	333	406 (5695)	389
**2Ba(OTf)_2_**	–1.77	0.34	3.7095(4)	0.0882	1554	555 (105)	607	344 (9358)	327	404 (5176)	386

^*a*^Reduction potentials referenced to Fe(C_5_H_5_)_2_^+/0^.

^*b*^Difference between the reduction potential of **2M** and Ni(3′-OCH_3_-salen).

^*c*^ *τ*_4_ value describing the coordination geometry around the Ni(ii) ions, where *τ*_4_ = 1 for a tetrahedral geometry and *τ*_4_ = 0 for a square planar geometry.

^*d*^From ref. 40.

^*e*^Assignment based on ref. 42.

### Electrochemistry

**Fig. 2:**
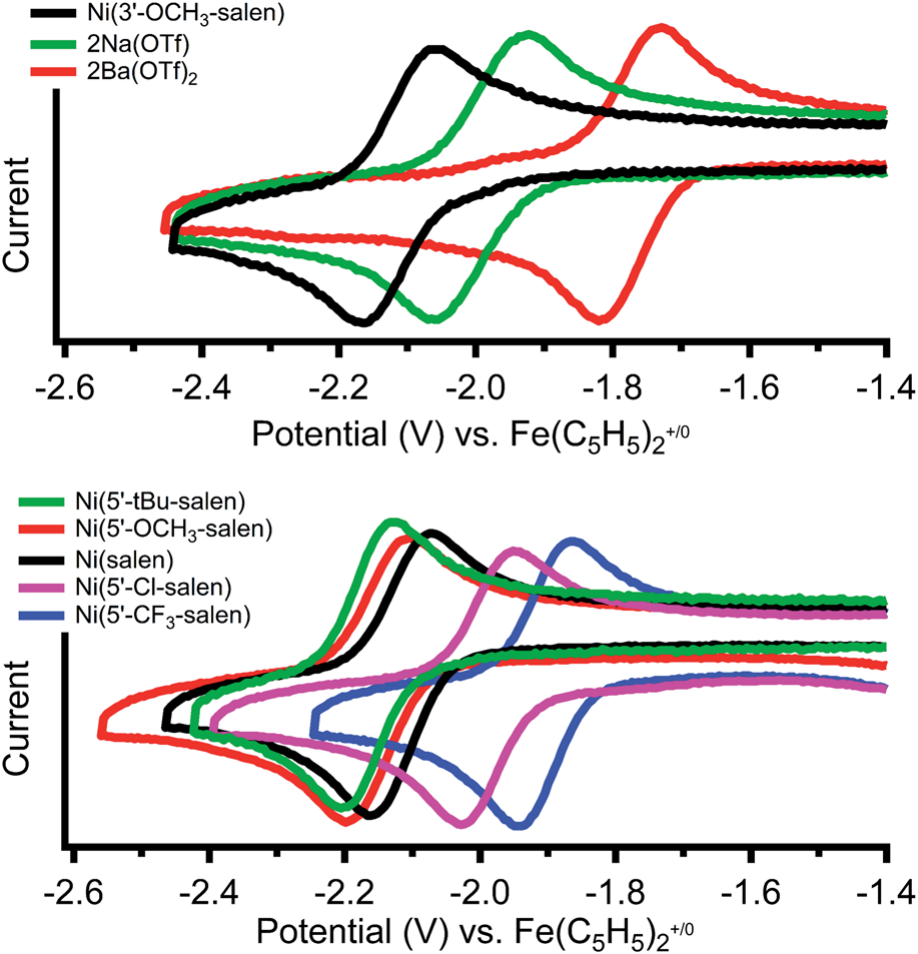
Cyclic voltammograms of the reversible Ni(ii/i) redox couples of (top) Ni(3′-OCH_3_-salen) and **2M** (M = Na^+^, Ba^2+^) and (bottom) Ni(5′-R-salen) where (R = CF_3_, Cl, *t*Bu, OCH_3_) in 0.1 M tetrabutylammonium hexafluorophosphate in dimethylformamide under N_2_ at a scan rate of 1 V s^–1^.

Cyclic voltammetry of Ni(3′-OCH_3_-salen) in dimethylformamide *versus* Fe(C_5_H_5_)_2_^+/0^ reveals a reversible reduction at –2.11 V that we assign to a Ni(ii/i) redox event (Fig. 2, top, individual CVs are shown in Fig. S26–S28†). The corresponding Ni(ii/i) reduction events in **2Na** and **2Ba** are also reversible and found at –1.99 and –1.77 V, respectively. Compared to Ni(3′-OCH_3_-salen), the redox potential for **2Na** and **2Ba** are shifted positive by 120 mV and 340 mV, respectively. The increased difference in redox potential correlates with increased cationic charge, which we have also observed in similar complexes.^36^

Cyclic voltammetry of the Ni(5′-R-salen) (R = CF_3_, Cl, *t*Bu, OCH_3_) compounds are also reversible as shown in Fig. 2 (bottom) and summarized in Table 2. As expected, the appended inductive groups result in changes to the Ni(ii/i) reduction potential. Compared to Ni(salen), addition of the trifluoromethyl and chloro electron-withdrawing substituents resulted in positive shifts in redox potential by 190 mV and 140 mV, respectively. Conversely, the addition of the methoxy and *tert*-butyl electron-donating groups caused the redox potential to shift negatively by 50 mV and 90 mV, respectively.

**Table 2:** Summary of structural, spectroscopic, and electrochemical data for Ni(5′-R-salen). Electrochemical and electronic absorption spectroscopy were taken in dimethylformamide

Complex	*E*_1/2_ Ni(ii/i)^*a*^, V	*ν*(C=N), cm^–1^	*λ*(d → d)^*b*^, nm (*ε*, M^–1^ cm^–1^)	*λ*(π–π*)^*b*^, nm (*ε*, M^–1^ cm^–1^)	*λ*_MLCT_ ^*b*^, nm (*ε*, M^–1^ cm^–1^)
Ni(5′-*t*Bu-salen)	–2.17	1525	538 (163)	335 (9328)	416 (7071)
Ni(5′-OCH_3_-salen)	–2.15	1538	551 (200)	344 (5947)	434 (6932)
Ni(salen)	–2.12	1533	532 (141)	334 (8500)	410 (6548)
Ni(5′-Cl-salen)	–1.99	1527	534 (180)	337 (7738)	417 (6205)
Ni(5′-CF_3_-salen)	–1.91	1541	528 (144)	336 (6249)	400 (5289)

^*a*^Reduction potentials referenced to Fe(C_5_H_5_)_2_^+/0^.

^*b*^Assignment based on ref. 42.

### Electronic and vibrational spectroscopy

Solid state infrared spectroscopy of the complexes was taken to compare the electronic environment of the nickel center (Fig. S29–S32†). As cationic charge increases, the vibrational frequency of the imine C=N stretch increases (Table 1). Ni(5′-R-salen) displayed shifts in vibrational frequency of the C=N stretch over a range of 14 cm^–1^ compared to Ni(salen). These shifts did not correlate with electron-donating or -withdrawing capability (Table 2).

The compounds were examined by electronic absorption and infrared spectroscopy to further probe the electronic effects of the Na^+^ and Ba^2+^ cation on the Ni(ii) ligand field. The electronic absorption spectra in dimethylformamide are shown in Fig. S10–S15† and are summarized in Table 1. The absorption spectrum of Ni(3′-OCH_3_-salen) displays a π → π* transition at 349 nm and a d → π* (MLCT) transition at 415 nm.^41^,^42^ The **2M** complexes exhibit a slight blue shift in both their π → π* bands (≤5 nm) and MLCT bands (≤11 nm) relative to Ni(3′-OCH_3_-salen). Additionally, Ni(3′-OCH_3_-salen) has a d → d absorption band at 547 nm, which appears in **2M** with a small red shift (≤8 nm). Molar absorptivity for each transition for **2M** is comparable to Ni(3′-OCH_3_-salen). Changes in absorption in **2M** compared to Ni(3′-OCH_3_-salen) indicate that the presence of M has a small influence on the ligand field around the Ni(ii) center.

The electronic absorption spectra of the monometallic compounds are shown in Fig. S16–S25† and are summarized in Table 2. Ni(salen) displays a d → d absorption band at 532 nm. Compared to Ni(salen), the same transition appears in the Ni(5′-R-salen) complexes with a small red shift (≤9 nm) with the exception of Ni(5′-OCH_3_-salen) which shifted this transition by 23 nm. As expected, modifying the ligand with electron withdrawing functionalities shifts the reduction potential positive. The inductive effect on the ligand field is reflected in the increasing energy of the d–d band, which correlates to changes in the HOMO–LUMO gap (although R = OCH_3_ does not fit the trend). In contrast, addition of the cations in **2M** show slightly smaller changes in their d–d band absorption despite larger changes in reduction potential. The absorption wavelength for **2M** also shifts to slight lower energies as the reduction potential shifts positive, or the opposite trend observed in the monomeric complexes.

## Computational studies

### Nickel complexes

To clarify the effect of the secondary cation, density functional theory (DFT) calculations were performed using the ωB97X-D functional.^43^ The def2-SVP basis set was used for geometry optimizations and the def2-TZVP basis for final electronic energies and spectra.^44^ Solvent was modeled using the SMD solvent model^45^ with the corresponding parameters for dimethylformamide or acetonitrile. Calculations were performed using Gaussian 09 (ref. 46) and electrostatic potential maps were plotted using ChemCraft.^47^

**Fig. 3:**
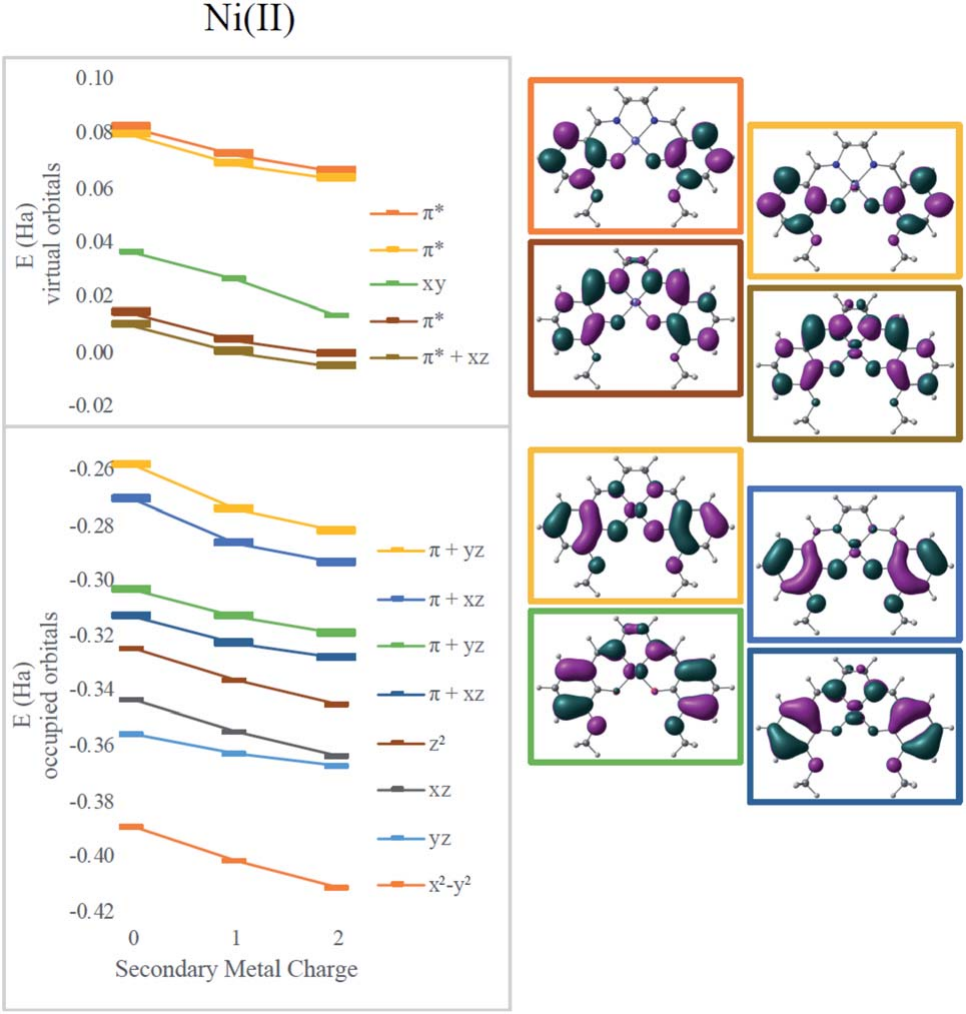
Effect of secondary metals on orbital energies in Ni complexes. Orbitals involved in MLCT are bolded in the graph and plotted on the right for the Ni(salen–OMe) complex.

Electronic absorption spectra simulated using time-dependent DFT (TD-DFT)^48^ showed fairly good agreement with experimental spectra with errors less than 0.2 eV (typical errors for TD-DFT being 0.1–0.5 eV);^48^ only small shifts in wavelengths were observed for different secondary cations (Table 1). However, all the frontier orbitals shift in energy by a similar magnitude with increasing cationic charge, as shown in Fig. 3. The small relative changes in orbital energies is consistent with the modest differences observed in absorption spectra. In addition, the energies of all the MOs near the HOMO–LUMO gap trend in response to the field in a nearly parallel fashion. Hence, minimal spectral changes can be seen. Both the experimental and computational results indicate the major impact of the secondary cations is electrostatic as they do not significantly alter the ligand field at the redox site. Instead, as the LUMO energy decreases from Ni(3′-OCH_3_-salen) to **2Na** (by –0.27 eV) and to **2Ba** (by –0.42 eV), the reduction potential shifts positive.

### Iron complexes

The synthesis, structure, and characterization of several iron analogues were previously reported.^37^ Specifically, complexes with M_1_ = Fe(ii)(CH_3_CN) or Fe(iii)Cl and M_2_ = K^+^ and Ba^2+^ were prepared. Both the Fe(ii) and Fe(iii) complexes displayed a similar trend in redox potential to the Ni complexes (*i.e.* positive shift was larger for M_2_ = Ba^2+^ than M_2_ = K^+^ and both were shifted relative to the congener without any proximate cation as described in Table 3). The Fe(iii/ii) reduction potential of M_1_ = Fe(ii)(CH_3_CN) were shifted by 440 mV (M_2_ = K^+^) and 640 mV (M_2_ = Ba^2+^) more positive than Fe(ii)(salen). In contrast, when M_1_ = Fe(iii)Cl, the equivalent potential was only 70 mV (M_2_ = K^+^) and 230 mV (M_2_ = Ba^2+^) more positive compared to Fe(iii)(salen)Cl. Both reductions are electrochemically reversible, indicating ligand loss upon electron transfer is not impacting the observed reduction potentials. Hence, unlike in the Ni(ii/i) case, the pure effect of the secondary metal gets entangled with the effect of the auxiliary ligand on Fe, suggesting that the intramolecular field effects are affected. We sought to explain the difference between the Ni and Fe complexes using computational techniques.

**Table 3:** Structural and redox properties from the previously reported iron complexes in this ligand framework from ref. 37

Compound (M_1_–M_2_)	Fe–M distance, Å	*τ*_5_ ^*a*^	Fe–Cl distance, Å	*E*_1/2_ (Fe(iii/ii))^*b*^, V
Fe(ii)(CH_3_CN)–K^+^	—	—	—	–0.29
Fe(ii)(CH_3_CN)–Ba^2+^	3.6592(4)	0.11	—	–0.09
Fe(ii)(salen)	—	—	—	–0.73
Fe(iii)Cl–K^+^	3.6596(6)	0.36	2.218	–0.69
Fe(iii)ClBa^2+^	3.8115(3)	0.13	2.228	–0.53
[Fe(iii)(Cl)(salen)]	—	0.20	2.238	–0.76^102^

^*a*^ *τ*_5_ = 0 for a square pyramidal geometry and *τ*_5_ = 1 for a trigonal bipyramidal geometry.

^*b*^Reduction potentials in acetonitrile referenced to ferrocene/ferrocenium.

**Fig. 4:**
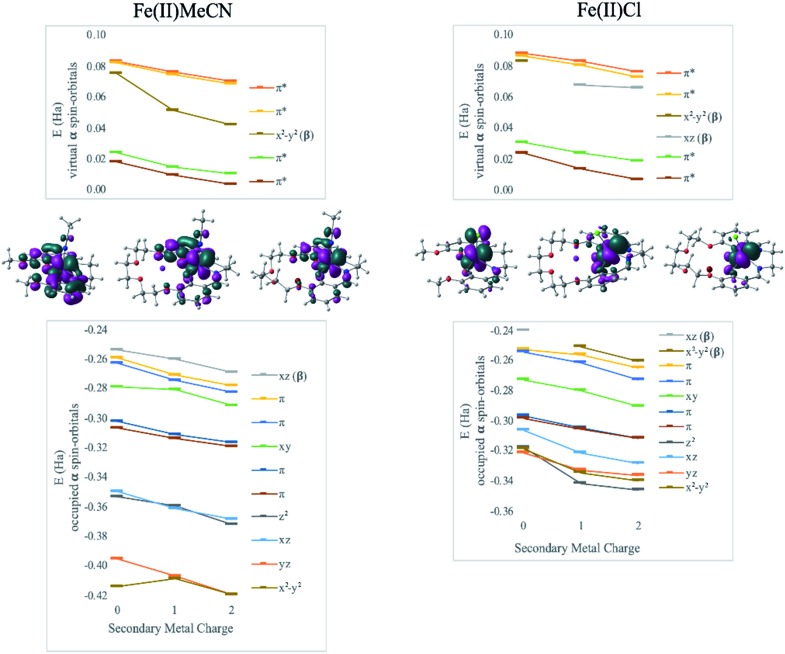
Effect of secondary metals on orbital energies in Fe(ii)MeCN and Fe(ii)Cl complexes. Geometries and HOMOs are depicted, to show the lack of geometric distortion in Fe(ii)MeCN and distortion in Fe(ii)Cl: Cl^–^ bends toward the secondary metal due to electrostatic attraction, while Cl withdraws from the participation in the HOMO. All orbitals trend down in energy as a function of the charge of the secondary metal, indicating the consistent effect of the intramolecular electric field.

DFT calculations revealed geometry distortions in the Fe(iii)Cl complexes in the presence of the secondary metals. In the reduced form of each complex, the chloride ligand was bent toward the secondary metal, significantly distorting the square pyramidal ligand field, and considerably reducing the ligand field splitting (notice a much more compressed MO manifold in this case, Fig. 4). The distortion causes electronic changes to the redox active metal and its coordination. The HOMO of Fe(ii)salen, which is also the LUMO of Fe(iii)salen, is delocalized over the d-AO on Fe and the p-AO on Cl. As the distortion occurs in the presence of the secondary metal, the HOMO acquires a pure d-character. Due to symmetry breaking, the nature of the HOMO in Fe(ii)Cl changes from d_*xz*_ in Fe(ii)salen to d_*x*^2^–*y*^2^_ when the secondary metal is present. Nevertheless, the field exerted by the secondary metal shifts all the orbital energies in a predictable downward trend with the increase of the charge of the secondary metal. Furthermore, the trend lines for MO energies as a function of the secondary metal charge have similar slopes for the two Fe(ii) complexes, indicating that the field experienced by Fe is similar in strength in the two complexes.

Hence, the redox-inactive cation may cause not only the shift in orbital energies by exerting an electric field, but also a significant perturbation to geometric and electronic structures of the redox-active metal. In this case, the redox-active metal structurally distorts as the anionic axial chloride ligand moves towards the cation. When the axial ligand is missing, as in the case of Ni, or does not hold a negative charge, as in Fe(ii)CH_3_CN, the isolated field effect of the secondary metal is seen. Hence, the ligand environment must be sufficiently rigid for the addition of a secondary metal to have a purely electrostatic effect. However, even accounting for geometric effects the electrostatic effect is still robust and predictable.

**Fig. 5:**
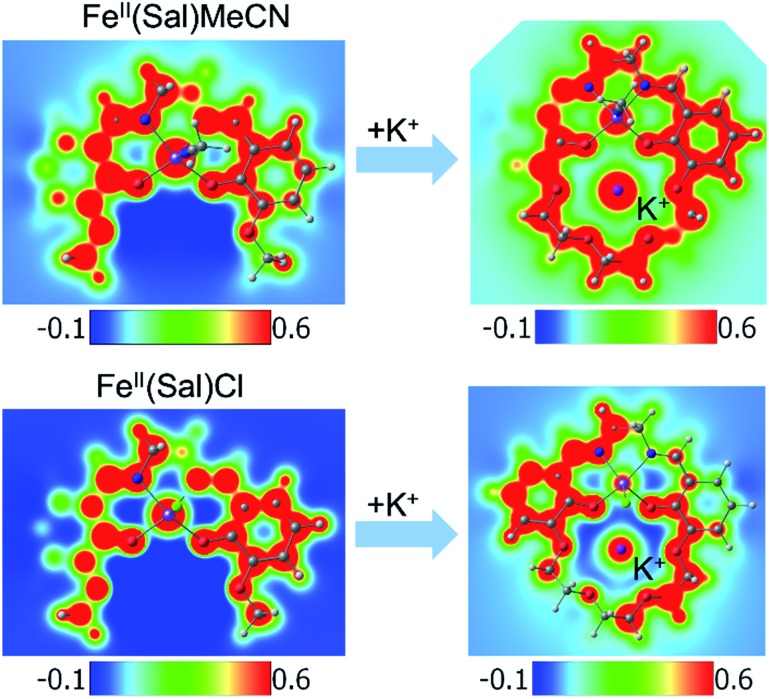
Electrostatic potential maps. (Top) Fe(ii)(sal)CH_3_CN and (bottom) Fe(ii)(sal)Cl complexes, without (left) and with (right) K^+^ in the secondary coordination sphere. The ligand for the complexes is 3′-OCH_3_-salen (left) or salen-crown (right). In the case of CH_3_CN, the metal coordination appears minimally disturbed, apart from the global shift in the magnitude of the electrostatic potential. In the case of Cl, the global shift is smaller, and the metal coordination appears visibly perturbed by the presence of K^+^.

Another way to illustrate the presence or absence of significant distortions to the electronic structure of the redox site is the electrostatic potential maps of the complexes (Fig. 5). For Fe(ii) complexes with acetonitrile, the maps for Fe(3′-OCH_3_-salen) and Fe(ii)(CH_3_CN)–K^+^ look nearly indistinguishable at first sight, apart from the magnitude of the potential (seen in the scale bars), confirming a clean intramolecular field effect of the secondary metal. In the case of the Fe(ii)Cl analogues, the change of the magnitude of the electrostatic potential upon the addition of the secondary metal is still seen, but smaller. However, the potential gains a significant asymmetry, in accord with the changing geometry of the complex.

## Conclusions

Our study demonstrates the use of proximal cations to install directional internal electric fields in transition metal complexes and the resulting effects on electronic structure. Computational methods quantified the impact of the field, which shifts the frontier orbitals to a similar degree while their relative energies are minimally affected. For the nickel complexes, the field shifts the reduction potentials by up to 340 mV with minimal changes to the ligand field. Computational methods were also used to investigate the variable shift in the Fe(iii/ii) potential in the presence of a neutral (CH_3_CN) or anionic (Cl^–^) axial ligand. We find the anionic ligand is involved in a structural distortion of the Fe(ii) complex in response to the direction of the electrostatic field, which also impacts the electronic structure. Although, the Fe experiences the effects of the field in both cases, the structural distortion results in a smaller positive shift in redox potential for the Cl^–^ analogue (70 mV for K^+^ and 230 mV for Ba^2+^) *versus* the CH_3_CN analogue (440 mV for K^+^ and 640 mV for Ba^2+^).

Even with some geometric distortion in the framework, it is clear the cation exerts significant internal electric fields. Consistent with an electrostatic effect, the impact of a dication is always larger than the monocation. Evidence for the electric field by changes in the redox potential is also notable because the measurements are made in solutions containing ∼100-fold excess of electrolyte. Hence, the rigid positioning of the cation and molecular architecture results in an electric field that is not easily quenched by solvation or presence of ions in high concentrations.

Electric fields have emerged as an important tool for accelerated or selective reactivity.^8^–^32^ We have previously described examples of how these types of cationic compounds disrupt traditional linear free energy relationships.^37^,^49^ Other potential applications of directional fields include stabilizing polar transition states to improve reaction rates. Additionally, directional fields can enable product selectivity by selectively stabilizing intermediates that would otherwise be comparable in energy in the absence of a field. Rate acceleration and improved selectivity have been demonstrated using *external* electric fields. This study demonstrates how the magnitude and direction of *internal* electric fields can be controlled through synthetic design. Incorporating a field *into each reaction site* does not require any external distance-dependent field source and can now be scaled.

## Conflicts of interest

There are no conflicts to declare.

## Supplementary Material

Supplementary information

Crystal structure data
