# Supplementary material for: Installation of internal electric fields by non-redox active cations in transition metal complexes
Source: Chem Sci. 2019 Sep 9;10(43):10135–42. doi: 10.1039/c9sc02870f (PMC6968733; doi:10.1039/c9sc02870f)
Supplement: Supplementary file 1 [file SC-010-C9SC02870F-s001.pdf]

Electronic Supplementary Material (ESI) for Chemical Science.
This journal is © The Royal Society of Chemistry 2019

Installation of an Internal Electric Fields by Non-Redox Active Cations in Transition Metal Complexes

Kevin Kang,[†a] Jack Fuller III,[†b] Alexander H. Reath,[a] Joseph W. Ziller,[a] Anastassia N. Alexandrova,[b,c]* Jenny Y. Yang[a]*

[a.] Department of Chemistry, University of California, Irvine 92697
[b.] Department of Chemistry and Biochemistry, University of California, Los Angeles, Los Angeles, CA, 90095, USA
[c.] California NanoSystems Institute, Los Angeles, CA, 90095, USA
† These authors contributed equally

Table of Contents

Experimental Details & Synthetic Procedures....3
Figure S1. ^{1}H NMR spectra of **Ni(3'-OCH$_3$-salen)** in CD_3CN. 7
Figure S2. ^{1}H NMR spectra of **2Na(OTf)** in CD_3CN. 7
Figure S3. ^{1}H NMR spectra of **2Ba(OTf)$_2$** in CD_3CN. 8
Figure S4. ^{1}H NMR spectra of **Ni(5'-*t*Bu-salen)** in $(CD_3)_2SO$. 8
Figure S5. ^{1}H NMR spectra of **Ni(5'-OCH$_3$-salen)** in $(CD_3)_2SO$. 9
Figure S6. ^{1}H NMR spectra of **Ni(salen)** in $(CD_3)_2SO$. 9
Figure S7. ^{1}H NMR spectra of **Ni(5'-Cl-salen)** in $(CD_3)_2SO$. 10
Figure S8. ^{1}H NMR spectra of **Ni(5'-CF$_3$-salen)** in $(CD_3)_2SO$. 10
Figure S9. ^{1}H NMR spectra of **5'-CF$_3$-salen** in $(CD_3)_2SO$. 11
Figure S10. UV-vis spectrum of **Ni(3'-OCH$_3$-salen)**, 37.5-50.0 μM in *N,N*-dimethylformamide. 11
Figure S11. UV-vis spectrum of **Ni(3'-OCH$_3$-salen)**, highlighting the d→d absorption band, 375-500 μM in *N,N*-dimethylformamide. 12
Figure S12. UV-vis spectrum of **2Na(OTf)**, 37.5-50.0 μM in *N,N*-dimethylformamide. 12
Figure S13. UV-vis spectrum of **2Na(OTf)**, highlighting the d→d absorption band, 375-500 μM in *N,N*-dimethylformamide. 13
Figure S14. UV-vis spectrum of **2Ba(OTf)$_2$**, 37.5-50.0 μM in *N,N*-dimethylformamide. 13
Figure S15. UV-vis spectrum of **2Ba(OTf)$_2$**, highlighting the d→d absorption band, 375-500 μM in *N,N*-dimethylformamide. 14
Figure S16. UV-vis spectrum of **Ni(5'-*t*Bu-salen)**, 37.5-50.0 μM in *N,N*-dimethylformamide. 14
Figure S17. UV-vis spectrum of **Ni(5'-*t*Bu-salen)**, highlighting the d→d absorption band, 375-500 μM in *N,N*-dimethylformamide. 15
Figure S18. UV-vis spectrum of **Ni(5'-OCH$_3$-salen)**, 37.5-50.0 μM in *N,N*-dimethylformamide. 15
Figure S19. UV-vis spectrum of **Ni(5'-OCH$_3$-salen)**, highlighting the d→d absorption band, 375-500 μM in *N,N*-dimethylformamide. 16
Figure S20. UV-vis spectrum of **Ni(salen)**, 37.5-50.0 μM in *N,N*-dimethylformamide. 16
Figure S21. UV-vis spectrum of **Ni(salen)**, highlighting the d→d absorption band, 375-500 μM in *N,N*-dimethylformamide. 17
Figure S22. UV-vis spectrum of **Ni(5'-Cl-salen)**, 37.5-50.0 μM in *N,N*-dimethylformamide. 17
Figure S23. UV-vis spectrum of **Ni(5'-Cl-salen)**, highlighting the d→d absorption band, 375-500 μM in *N,N*-dimethylformamide. 18
Figure S24. UV-vis spectrum of **Ni(5'-CF$_3$-salen)**, 37.5-50.0 μM in *N,N*-dimethylformamide. 18

Figure S25. UV-vis spectrum of **Ni(5'-CF_3-salen)**, highlighting the d→d absorption band, 375-500 μM in *N,N*-dimethylformamide. 19
Figure S26. Cyclic voltammogram of **Ni(3'-OCH_3-salen)** in *N,N*-dimethylformamide. Current is given in μA. 19
Figure S27. Cyclic voltammogram of **2Na(OTf)** in *N,N*-dimethylformamide. Current is given in μA. 20
Figure S28. Cyclic voltammogram of **$2Ba(OTf)_2$** in *N,N*-dimethylformamide. Current is given in μA. 20
Figure S29. Solid state infrared spectrum of **Ni(3'-OCH_3-salen)**. 21
Figure S30. Solid state infrared spectrum of **2Na(OTf).** 21
Figure S31. Solid state infrared spectrum of **$2Ba(OTf)_2$**. 22
Figure S32. Overlay of the C=N vibrational stretches of **Ni(3'-OCH_3-salen)** and **2M** complexes. 22
Figure S33. Solid state infrared spectrum of **Ni(5'-*t*Bu-salen)**. 23
Figure S34. Solid state infrared spectrum of **Ni(5'-OCH_3-salen)**. 23
Figure S35. Solid state infrared spectrum of **Ni(salen)**. 24
Figure S36. Solid state infrared spectrum of **Ni(5'-Cl-salen)**. 24
Figure S37. Solid state infrared spectrum of **Ni(5'-CF_3-salen)**. 25
Figure S38. Overlay of the C=N vibrational stretches of **Ni(5'-R-salen)** complexes. 25
Figure S39. ORTEP of **2Na(OTf)**. Thermal ellipsoids are drawn to 50% probability. Hydrogen atoms and outersphere anions and solvent molecules have been omitted for clarity. 26
Crystallographic data tables for **2Na(OTf)** 26
Figure S40. ORTEP of **$2Ba(OTf)_2$**. Thermal ellipsoids are drawn to 50% probability. Hydrogen atoms and outersphere anions and solvent molecules have been omitted for clarity. 41
Crystallographic data tables for **$2Ba(OTf)_2$** 41
Comparison of ωB97X-D and PBE0 spectra for Ni(II) complexes 61
UωB97X-D/def2-TZVP//UωB97X-D/def2-SVP SMD(*N,N*-Dimethylformamide or Acetonitrile)-Solvated Electronic Energies (Hartrees), Free Energy Corrections (Hartrees), and Cartesian Coordinates (Å) 61

Experimental Details:

General Considerations: For syntheses using air and moisture sensitive reagents or products, manipulations were carried out in a glovebox or using standard Schlenk techniques under an inert atmosphere of nitrogen. Unless otherwise noted, all experiments were carried out at room temperature (21-24 °C). All solvents used were degassed by sparging with argon and dried by passing through columns of neutral alumina or molecular sieves. Deuterated acetonitrile was purchased from Cambridge Isotopes Laboratories, Inc. and was degassed and stored over activated 3 Å molecular sieves prior to use. Reagents were purchased from commercial vendors and used without further purification unless otherwise noted. 3,3'-(((ethane-1,2-diylbis(oxy))bis(ethane-2,1-diyl))bis(oxy))bis(2-hydroxybenzaldehyde) was synthesized according to a literature preparation[1] with the following modification: the crude product was purified by silica gel column chromatography using a ratio of ethyl acetate to hexanes of 1:1 that progressed to a ratio of 2:1.

Physical Methods: NMR spectra were taken on a 500 MHz Bruker Avance GN500 (^{1}H) with a BBO probe at 20 °C. Electrospray ionization mass spectrometry was performed using an ESI LC-TOF Micromass LCT 3 mass spectrometer. Elemental analysis was taken on a PerkinElmer 2400 Series II CHNS elemental analyzer. Infrared (IR) absorption measurements were taken as compressed solids on a Thermo Scientific Nicolet iS5 spectrophotometer with an iD5 ATR attachment. UV-vis spectra were collected in *N,N*-dimethylformamide solution using an Agilent Technologies Cary 60 UV−vis.

Electrochemical procedures: All measurements performed on a Pine Wavedriver 10 bipotentiostat with a 2 mm diameter glassy carbon disc working electrode, a glassy carbon counter electrode, and a Ag/Ag+ pseudoreference electrode separated from the bulk solution by a Vicor frit. Potentials were referenced to a ferrocene internal standard at 0 V, and all experiments were performed in dry, degassed acetonitrile at a concentration of 1 mM analyte and 0.1 M tetrabutylammonium hexafluorophosphate and at a 1 V/s scan rate unless otherwise noted.

X-ray Crystallographic Methods:

X-ray Data Collection, Structure Solution and Refinement for **2Na(OTf)**.

A violet crystal of approximate dimensions 0.759 x 0.342 x 0.208 mm was mounted on a glass fiber and transferred to a Bruker SMART APEX II diffractometer. The APEX2[2] program package was used to determine the unit-cell parameters and for data collection (15 sec/frame scan time for a sphere of diffraction data). The raw frame data was processed using SAINT[3] and SADABS[4] to yield the reflection data file. Subsequent calculations were carried out using the SHELXTL[5] program. The diffraction symmetry was 2/*m* and the systematic absences were consistent with the monoclinic space group $P2_1/n$ that was later determined to be correct.

The structure was solved by direct methods and refined on F^2 by full-matrix least-squares techniques. The analytical scattering [factors5] for neutral atoms were used throughout the analysis. Hydrogen atoms were located from a difference-Fourier map and refined (x,y,z and U_{iso}).

Least-squares analysis yielded wR2 = 0.0599 and Goof = 1.046 for 361 variables refined against 6354 data (0.73 Å), R1 = 0.0233 for those 5947 data with $I > 2.0\sigma(I)$.

X-ray Data Collection, Structure Solution and Refinement for **2Ba(OTf)$_2$**

A red crystal of approximate dimensions 0.204 x 0.366 x 0.367 mm was mounted in a cryoloop and transferred to a Bruker SMART APEX II diffractometer. The APEX2[1] program package was used to determine the unit-cell parameters and for data collection (15 sec/frame scan time for a sphere of diffraction data). The raw frame data was processed using SAINT[3] and SADABS[4] to yield the reflection data file. Subsequent calculations were carried out using the SHELXTL[5] program. There were no systematic absences nor any diffraction symmetry other than the Friedel condition. The centrosymmetric triclinic space group $P\bar{1}$ was assigned and later determined to be correct.

The structure was solved by direct methods and refined on F^2 by full-matrix least-squares techniques. The analytical scattering factors[5] for neutral atoms were used throughout the analysis. Hydrogen atom H(13) was located from a difference-Fourier map and refined (x,y,z and U_{iso}). The remaining hydrogen atoms were included using a riding model. The molecule was a dimer located about an inversion center. There were two non-coordinated triflate ions present per dimeric formula-unit.

Least-squares analysis yielded wR2 = 0.0594 and Goof = 1.054 for 456 variables refined against 7985 data (0.73 Å), R1 = 0.0219 for those 7701 data with $I > 2.0\sigma(I)$.

Definitions:

$wR2 = [\Sigma[w(F_o^2-F_c^2)^2] / \Sigma[w(F_o^2)^2]]^{1/2}$

$R1 = \Sigma||F_o|-|F_c|| / \Sigma|F_o|$

$Goof = S = [\Sigma[w(F_o^2-F_c^2)^2] / (n-p)]^{1/2}$ where n is the number of reflections and p is the total number of parameters refined.

The thermal ellipsoid plot is shown at the 50% probability level.

Synthetic Procedures:

Method 1. General procedure for synthesis of 2M: 1M (M = Na^+, Ba^{2+}) ligands were synthesized using a modified literature procedure.[1] In an inert atmosphere glovebox, 1 equivalent of **1M** (M = Na^+, Ba^{2+}) was dissolved in refluxing methanol. 1 equivalent of $Ni(OAc)_2$ was added dropwise in a concentrated methanol solution over several minutes, and the reaction was refluxed for ten minutes. The crude red product was isolated under reduced pressure at 80 °C, re-dissolved in methanol, and passed through a glass microfiber filter. Vapor diffusion of diethyl ether into the methanol solution precipitated the product as crystals, which were isolated by filtration and washed with diethyl ether.

2Na(OTf). Synthesized using method 1 with **1Na(OTf)** (0.010 g, 0.017 mmol) and $Ni(OAc)_2$ (0.003 g, 0.017 mmol) to give 0.009 g product (67% yield). ESI mass spectrometry: Calculated m/z for **(2Na(OTf)-CF_3SO_3]**$^+$: 493.09. Found: 493.09. Analytical calculation for $C_{23}H_{24}NaNiF_3O_9N_2S$: C, 42.95; H, 3.76; N, 4.36. Found: C, 4.95; H, 3.70; N, 4.19. ^{1}H NMR (500 MHz, CD_3CN) δ 7.77 (s, 2H), 6.95 (d, 2H), 6.90 (d, 2H), 6.62 (t, 2H), 4.07 (t, 4H), 3.84 (t, 4H), 3.72 (s, 4H), 3.47 (s, 4H).

2Ba(OTf)$_2$. Synthesized using method 1 with **1Ba(OTf)$_2$** (0.050 g, 0.059 mmol) and $Ni(OAc)_2$ (0.0146 g, 0.059 mmol) to give 0.042 g product (80% yield). ESI mass spectrometry: Calculated m/z for **2Ba**$^+$: 756.96. Found: 756.95. Analytical calculation for $C_{24}H_{24}BaNiF_6O_{12}N_2S_2$: C, 31.80; H, 2.67; N, 3.09. Found: C, 31.93; H, 2.67; N, 3.01. ^{1}H NMR (500 MHz, CD_3CN) δ 7.80 (s, 2H), 7.04 (t, 4H), 6.75 (t, 2H), 4.24 (t, 4H), 3.97 (t, 4H), 3.87 (s, 4H), 3.49 (s, 4H).

Synthesis of Salen Ligands. Synthesis of the 5'-R-salen ligands (R = t*Bu*, OCH_3, H, Cl) followed a literature procedure.[6]

5'-CF_3-salen. Synthesized using the same procedure[6] as the other salen ligands using 2-hydroxy-5-(trifluoromethyl)benzaldehyde (0.250 mg, 1.32 mmol) and ethylenediamine (0.040 g, 0.66 mmol) to give 220 mg of product (82.8 % yield). ^{1}H NMR (500 MHz, $(CD_3)_2SO$) δ 14.08 (s, 2H), 8.72 (s, 2H), 7.86 (s, 2H), 7.63 (m, 2H), 7.00 (d, 2H), 3.97 (s, 4H).

Method 2. General procedure for synthesis of monometallic Ni salen complexes followed a literature procedure.[7]

Ni(3'-OCH_3-salen). Synthesized using method 2 with 3'-OCH_3 salen (N,N'-bis(3-methoxysalicylidene)-1,2-diaminoethane) (0.05 g, 0.152 mmol) and $Ni(OAc)_2$ (0.038 g, 0.152 mmol) to give 47 mg product (80% yield). ESI mass spectrometry: Calculated m/z for Ni(3'-OCH_3-salen): 407.05. Found: 407.05. ^{1}H NMR (500 MHz, CD_3CN) δ 7.69 (s, 2H), 6.83 (d, 2H), 6.79 (d, 2H), 6.49 (t, 2H), 3.78 (s, 6H), 3.41 (s, 4H).

Ni(5'-*t*Bu-salen). Synthesized using method 2 with 5'-*t*Bu-salen (N,N'-bis(5-tertbutylsalicylidene)-1,2-diaminoethane) (0.150 g, 0.394 mmol) and $Ni(OAc)_2$ (0.0697 g, 0.394 mmol) to give 105 mg product (60.9.% yield). ^{1}H NMR (500 MHz, $(CD_3)_2SO$) δ 7.89 (s, 2H), 7.26 (m, 2H), 7.20 (d, 2H), 6.65 (d, 2H), 3.39 (s, 4H), 1.22 (s, 18H).

Ni(5'-OCH_3-salen). Synthesized using method 2 with 5'-OMe-salen (N,N'-bis(5-methoxysalicylidene)-1,2-diaminoethane) (0.200 g, 0.609 mmol) and $Ni(OAc)_2$ (0.1077 g, 0.609 mmol) to give 218 mg product (93.0% yield). ^{1}H NMR (500 MHz, $(CD_3)_2SO$) δ 7.85 (s, 2H), 6.88 (m, 2H), 6.78 (d, 2H), 6.64 (d, 2H), 3.65 (s, 6H), 3.40 (s, 4H). The ^{1}H NMR spectra of this compound is consistent with previously reported literature data.[8]

Ni(salen). Synthesized using method 2 with salen (N,N'-bis(salicylidene)-1,2-diaminoethane) (0.100g, 0.373 mmol) and $Ni(OAc)_2$ (0.066 g, 0.373 mmol) to give 108 mg product (89.1% yield). ^1H NMR (500 MHz, $(CD_3)_2SO$) δ 7.90 (s, 2H), 7.27 (m, 2H), 7.17 (m, 2H), 6.71 (d, 2H), 6.51 (t, 2H), 3.42 (s, 4H).

Ni(5'-Cl-salen). Synthesized using method 2 with 5'-Cl-salen (N,N'-bis(5-chlorosalicylidene)-1,2-diaminoethane) (0.200 g, 0.593 mmol) and $Ni(OAc)_2$ (0.105 g, 0.593 mmol) to give 219 mg product (93.8% yield). ^1H NMR (500 MHz, $(CD_3)_2SO$) δ 7.91 (s, 2H), 7.35 (d, 2H), 7.17 (m, 2H), 6.72 (d, 2H), 3.44 (s, 4H).

Ni(5'-CF_3-salen). Synthesized using method 2 with 5'-CF_3-salen (N,N'-bis(5-trifluoromethylsalicylidene)-1,2-diaminoethane) (0.100 g, 0.247 mmol) and $Ni(OAc)_2$ (0.0437 g, 0.247 mmol) to give 64 mg product (56.2 % yield). ^1H NMR (500 MHz, $(CD_3)_2SO$) δ 8.17 (s, 2H), 7.72 (s, 2H), 7.44 (m, 2H), 6.86 (d, 2H), 3.50 (s, 4H).

NMR Spectra

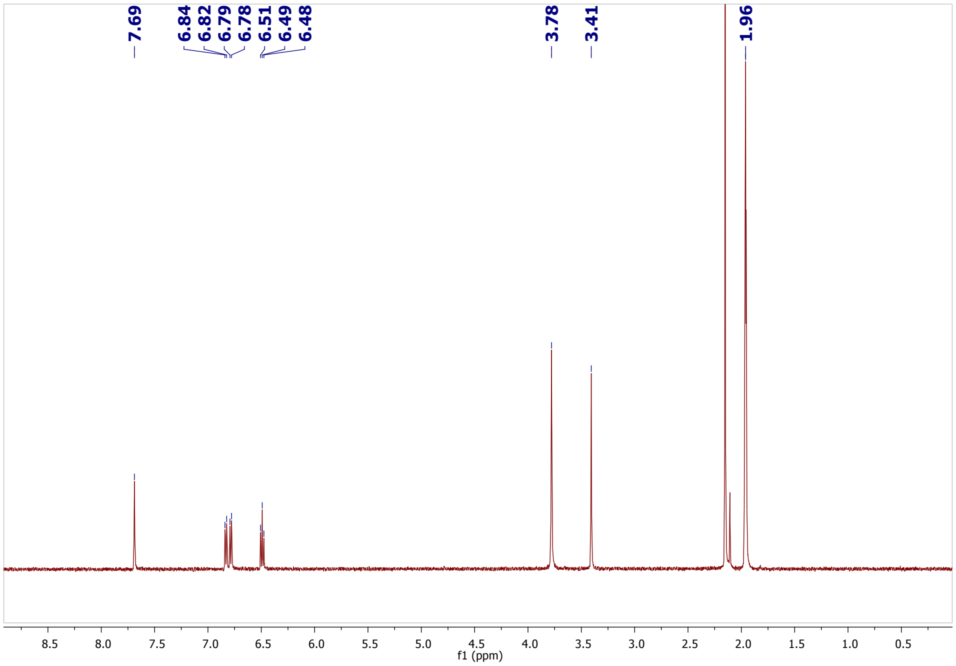

Figure S1. ^{1}H NMR spectra of **Ni(3’-OCH$_3$-salen)** in CD_3CN.

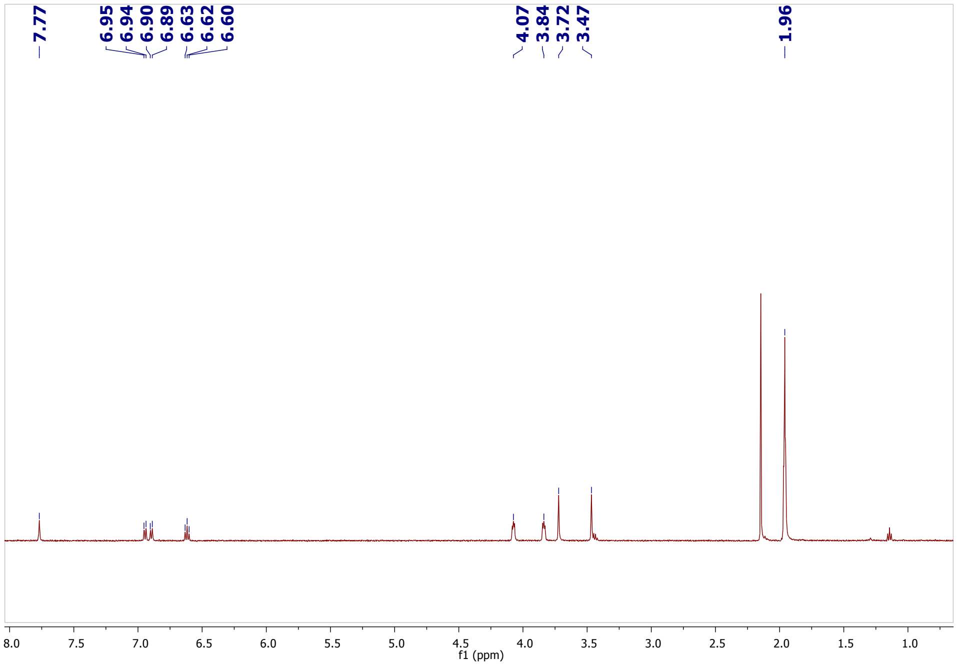

Figure S2. ^{1}H NMR spectra of **2Na(OTf)** in CD_3CN.

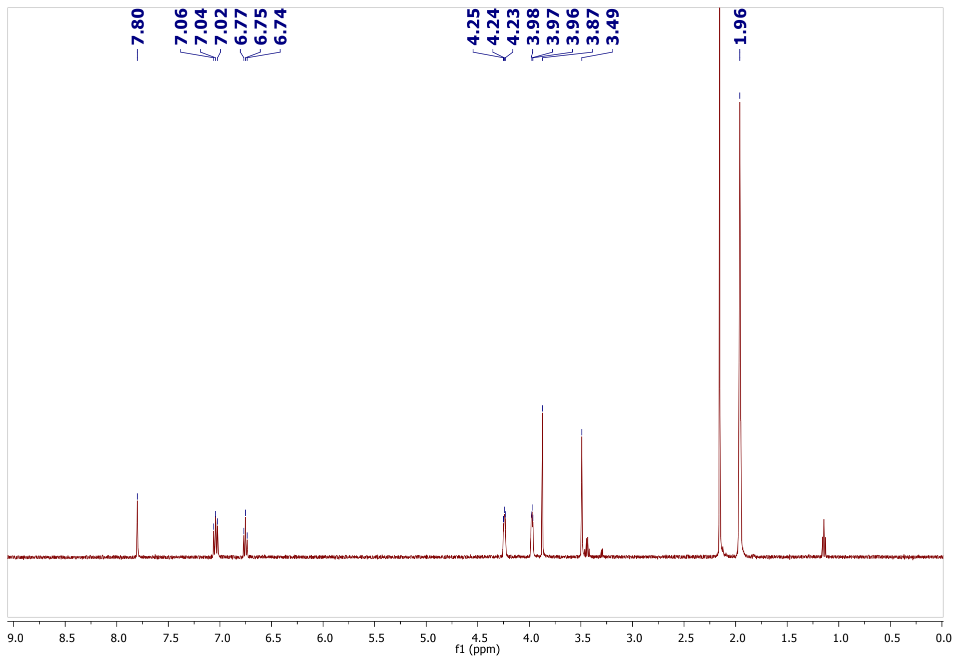

Figure S3. ^{1}H NMR spectra of **2Ba(OTf)$_2$** in CD_3CN.

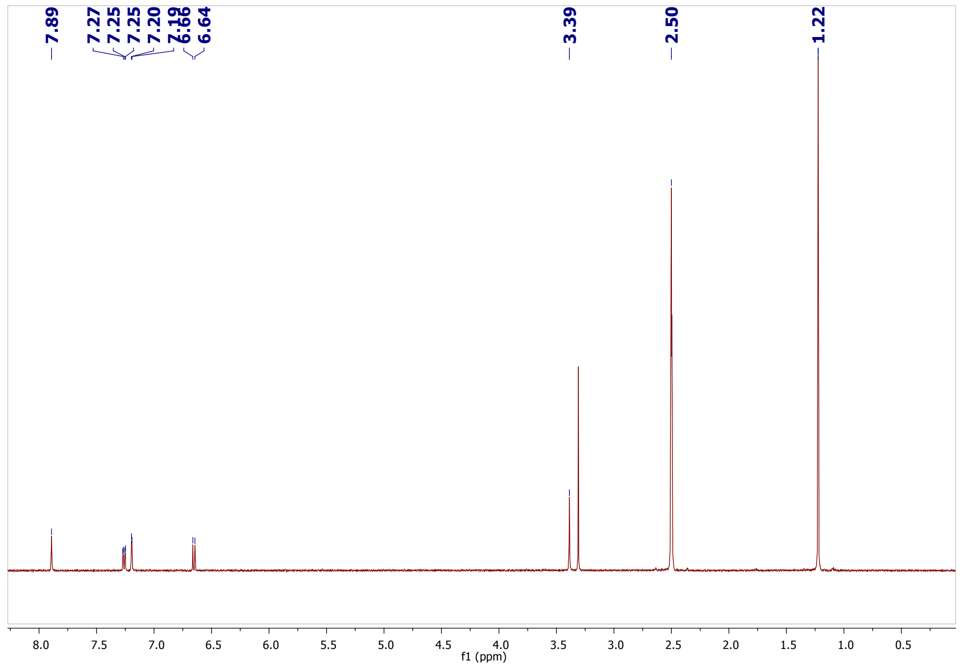

Figure S4. ^{1}H NMR spectra of **Ni(5’-*t*Bu-salen)** in $(CD_3)_2SO$.

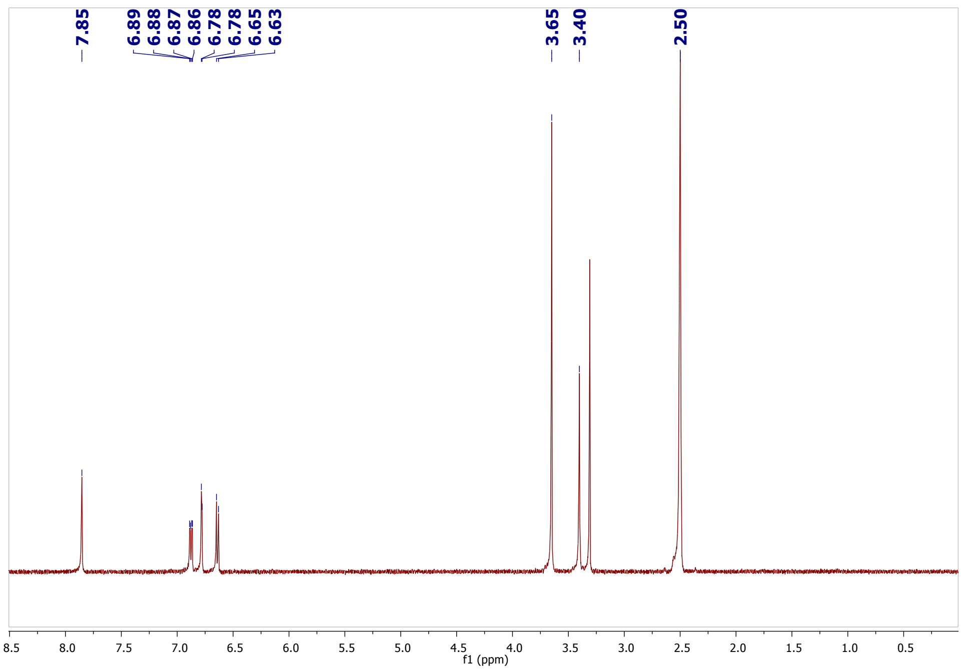

Figure S5. ^{1}H NMR spectra of **Ni(5'-OCH$_3$-salen)** in $(CD_3)_2SO$.

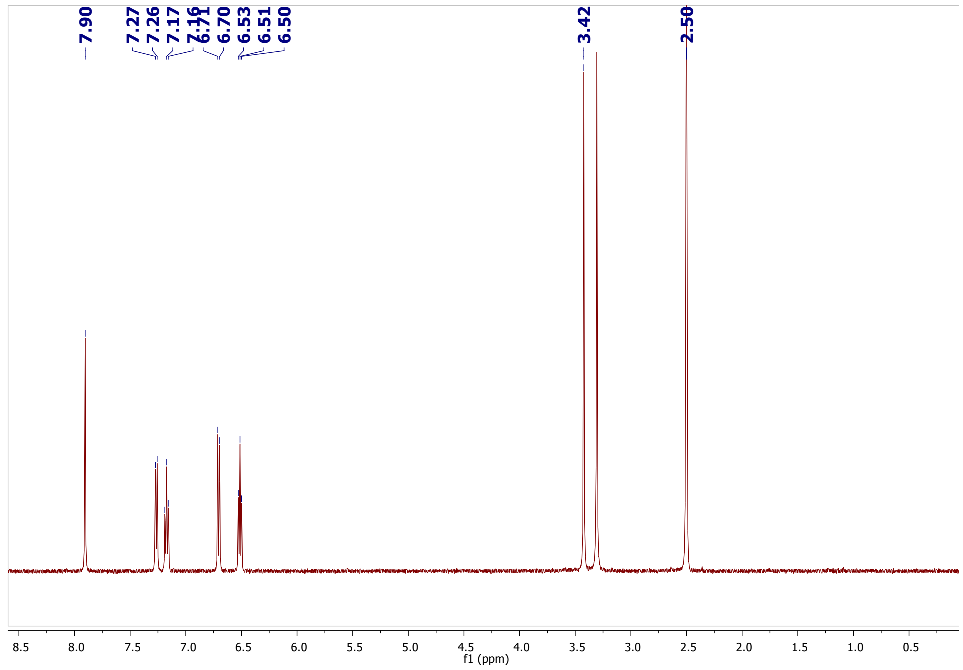

Figure S6. ^{1}H NMR spectra of **Ni(salen)** in $(CD_3)_2SO$.

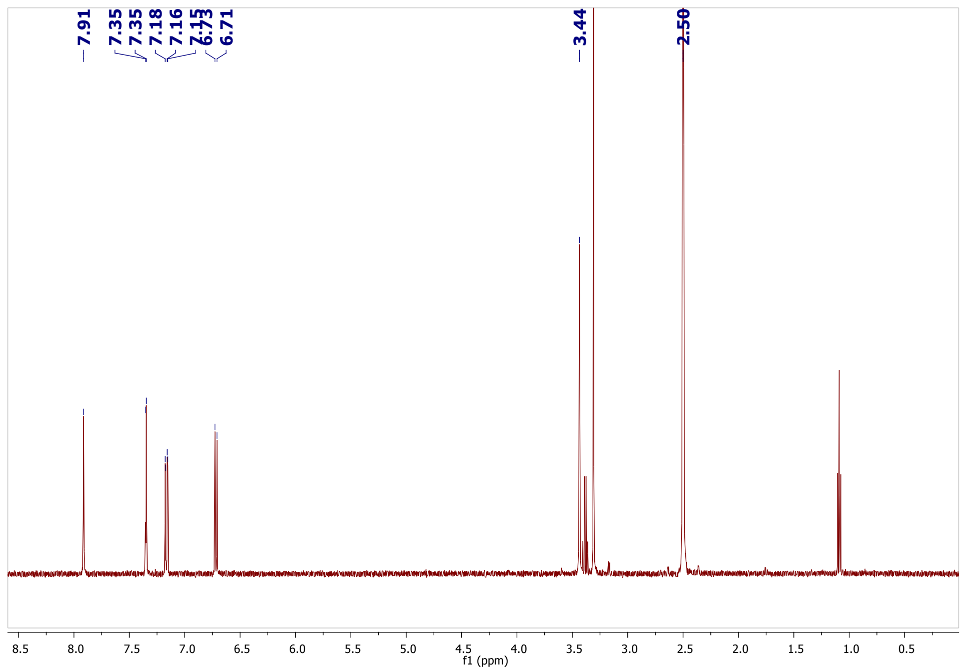

Figure S7. ^{1}H NMR spectra of **Ni(5’-Cl-salen)** in $(CD_3)_2SO$.

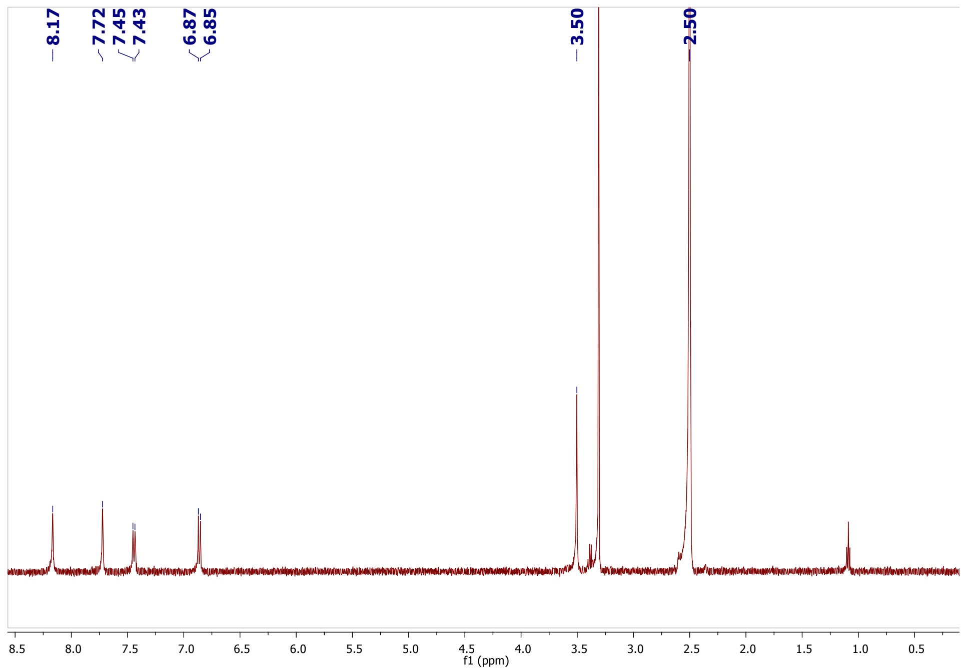

Figure S8. ^{1}H NMR spectra of **Ni(5’-CF_3-salen)** in $(CD_3)_2SO$.

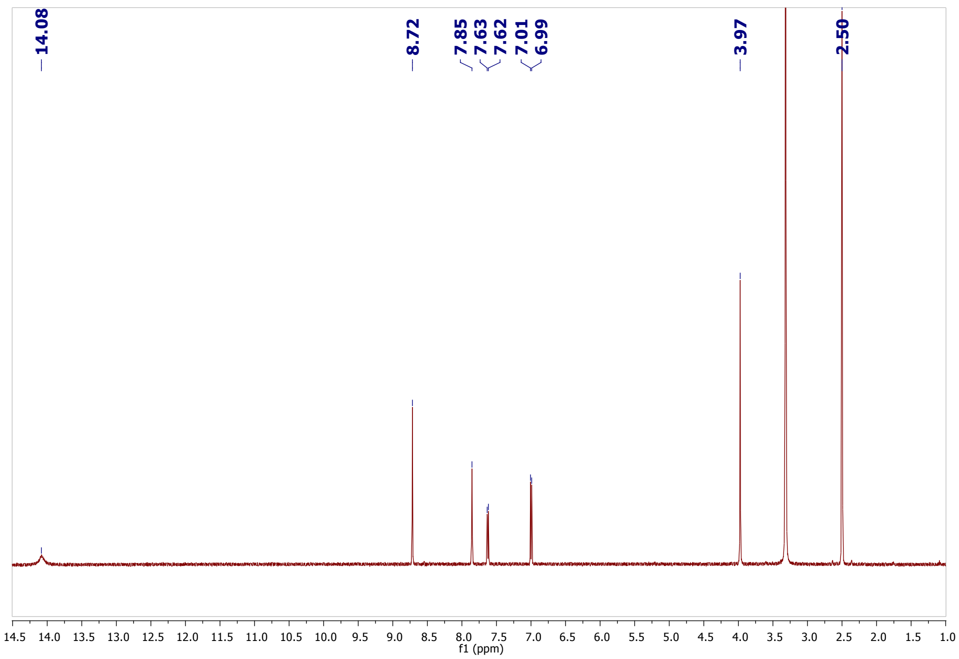

Figure S9. ^{1}H NMR spectra of **5'-CF$_3$-salen** in $(CD_3)_2SO$.

UV-vis Spectra

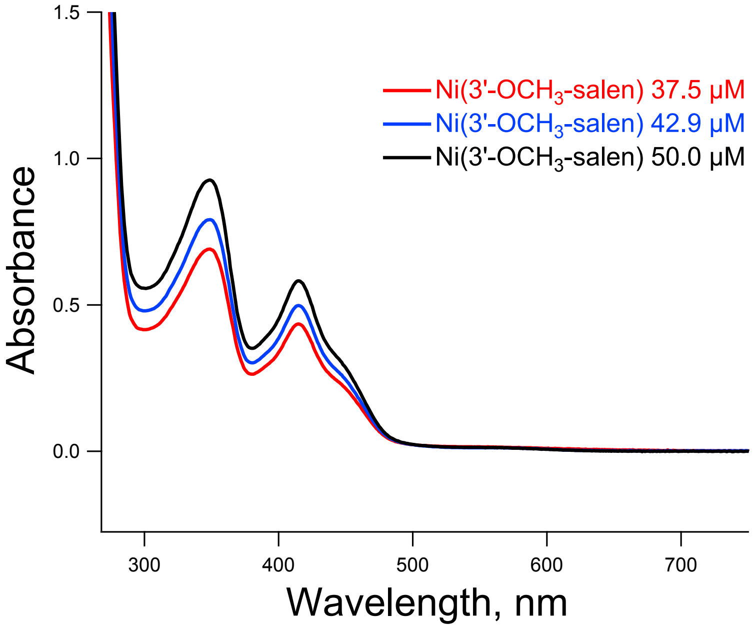

Figure S10. UV-vis spectrum of **Ni(3'-OCH$_3$-salen)**, 37.5-50.0 μM in *N,N*-dimethylformamide.

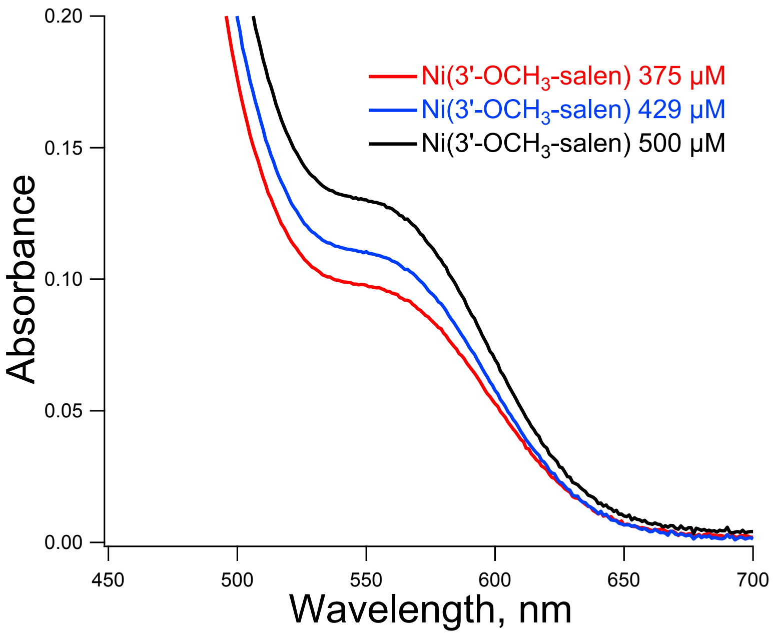

Figure S11. UV-vis spectrum of **Ni(3’-OCH$_3$-salen)**, highlighting the d→d absorption band, 375-500 µM in *N,N*-dimethylformamide.

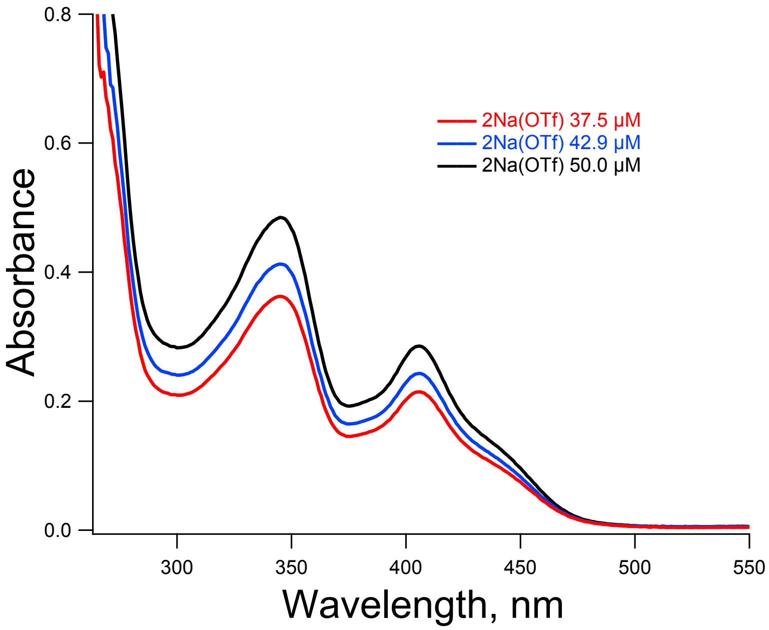

Figure S12. UV-vis spectrum of **2Na(OTf)**, 37.5-50.0 µM in *N,N*-dimethylformamide.

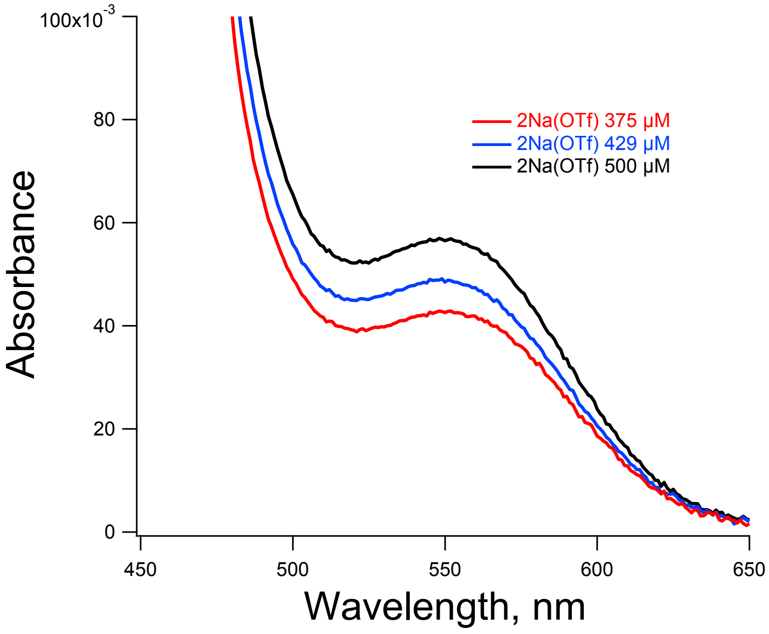

Figure S13. UV-vis spectrum of **2Na(OTf)**, highlighting the d→d absorption band, 375-500 µM in *N,N*-dimethylformamide.

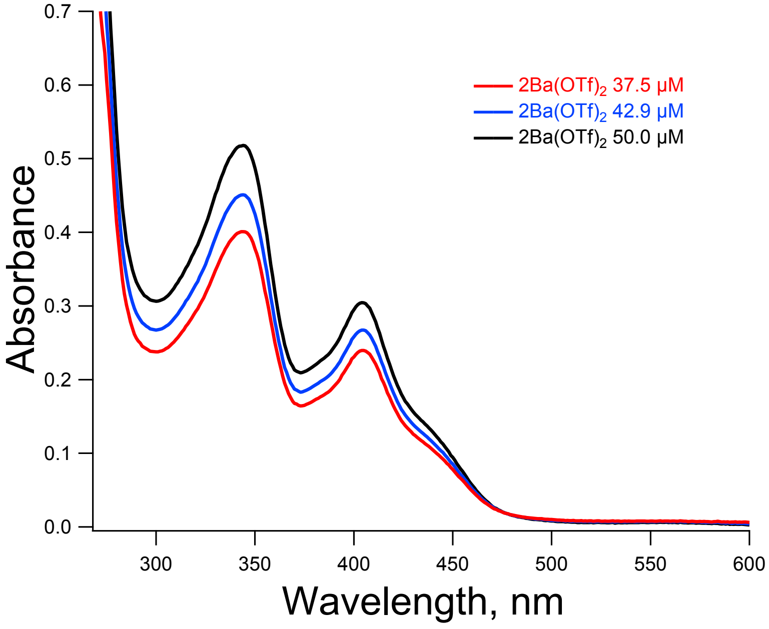

Figure S14. UV-vis spectrum of **$2Ba(OTf)_2$**, 37.5-50.0 µM in *N,N*-dimethylformamide.

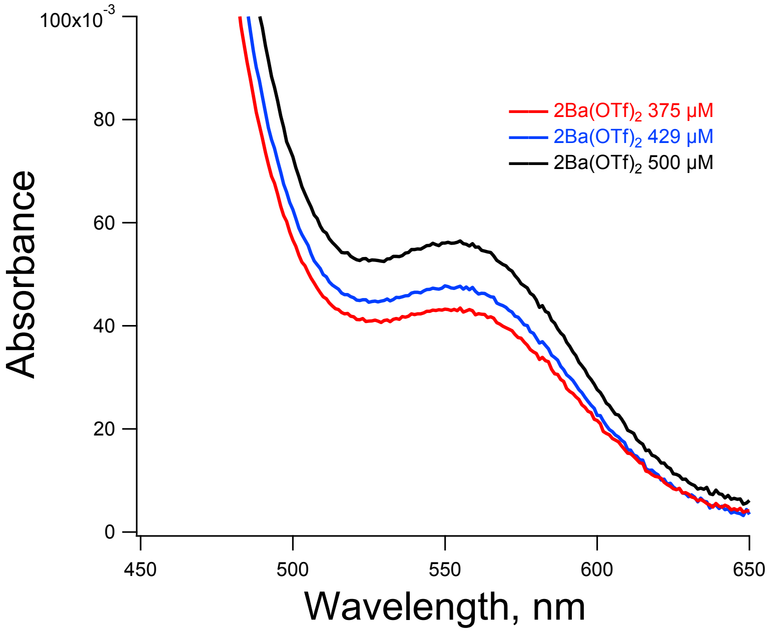

Figure S15. UV-vis spectrum of **2Ba(OTf)$_2$**, highlighting the d→d absorption band, 375-500 µM in *N,N*-dimethylformamide.

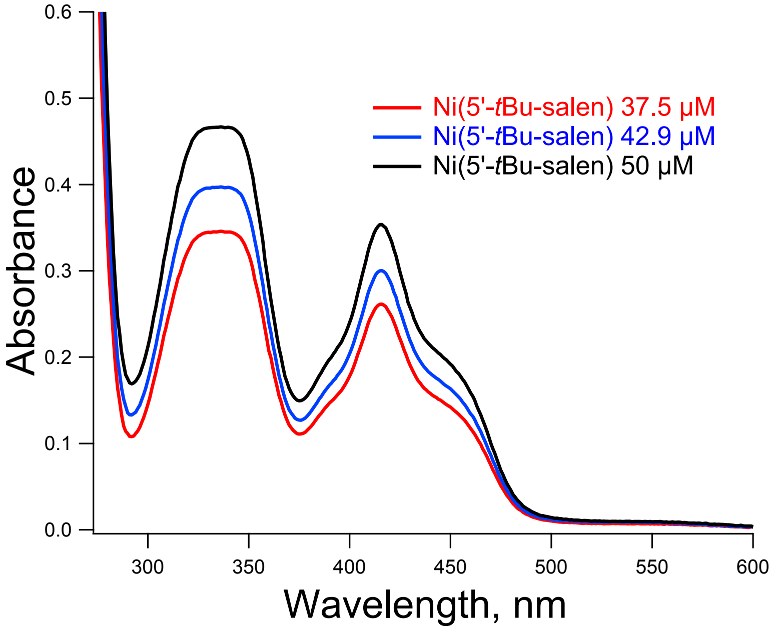

Figure S16. UV-vis spectrum of **Ni(5’-*t*Bu-salen)**, 37.5-50.0 µM in *N,N*-dimethylformamide.

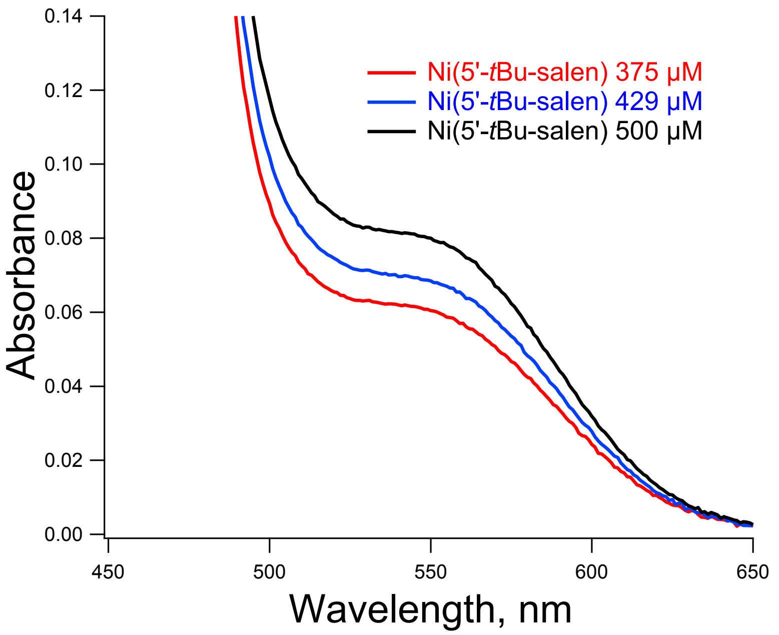

Figure S17. UV-vis spectrum of **Ni(5’-*t*Bu-salen)**, highlighting the d→d absorption band, 375-500 μM in *N,N*-dimethylformamide.

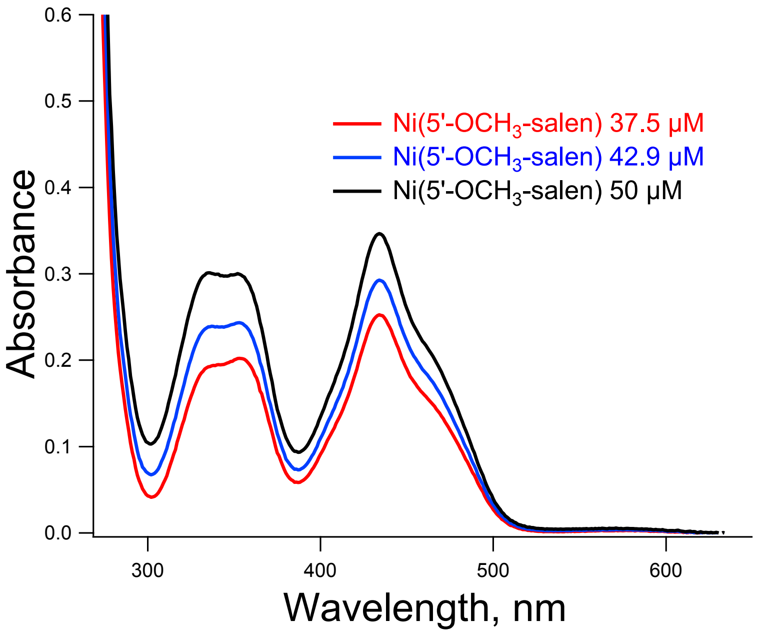

Figure S18. UV-vis spectrum of **Ni(5’-OCH_3-salen)**, 37.5-50.0 μM in *N,N*-dimethylformamide.

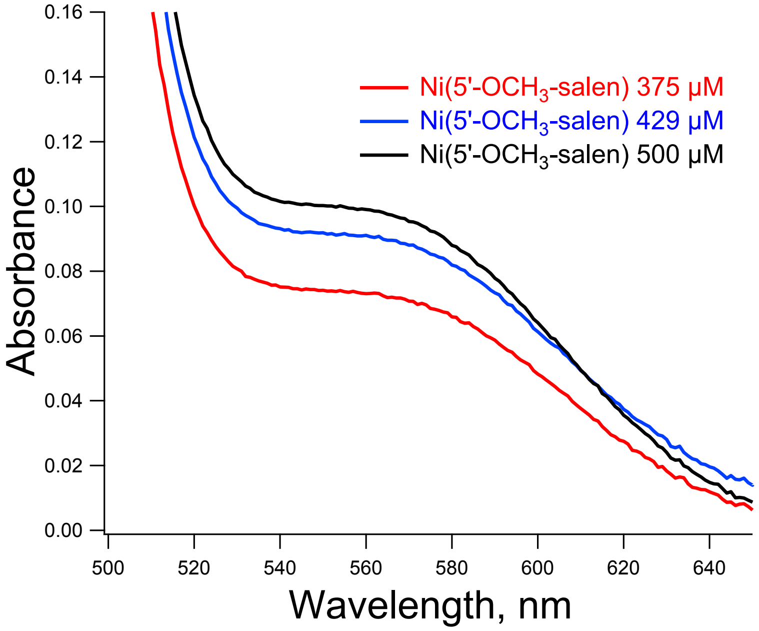

Figure S19. UV-vis spectrum of **Ni(5’-OCH$_3$-salen)**, highlighting the d→d absorption band, 375-500 µM in N,N-dimethylformamide.

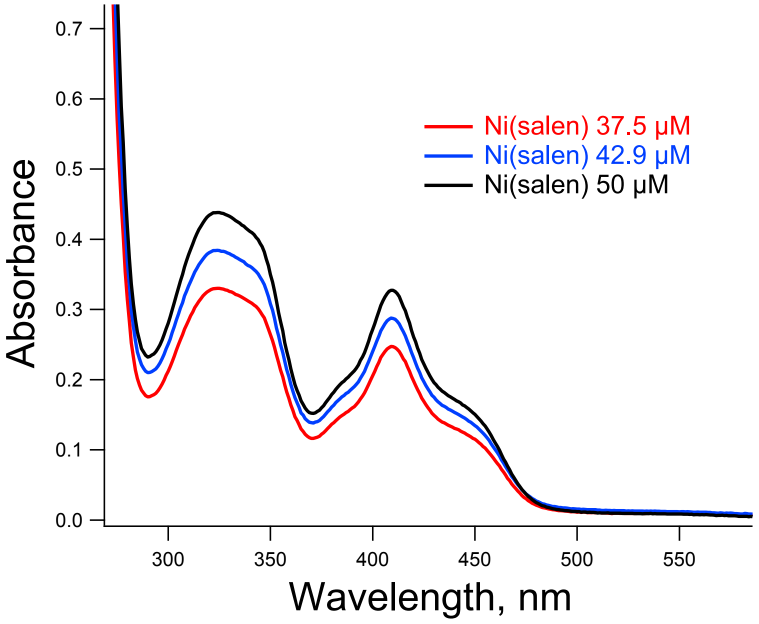

Figure S20. UV-vis spectrum of **Ni(salen)**, 37.5-50.0 µM in *N,N*-dimethylformamide.

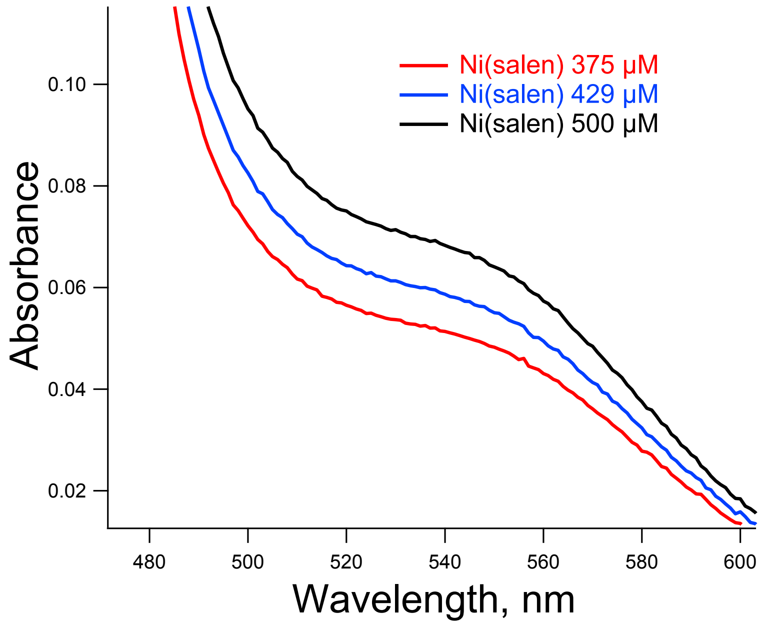

Figure S21. UV-vis spectrum of **Ni(salen)**, highlighting the d→d absorption band, 375-500 μM in *N,N*-dimethylformamide.

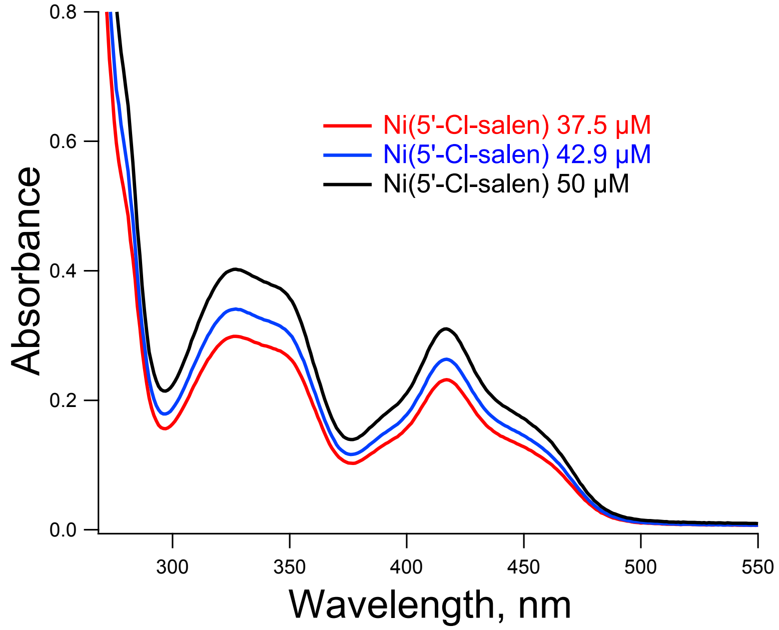

Figure S22. UV-vis spectrum of **Ni(5’-Cl-salen)**, 37.5-50.0 μM in *N,N*-dimethylformamide.

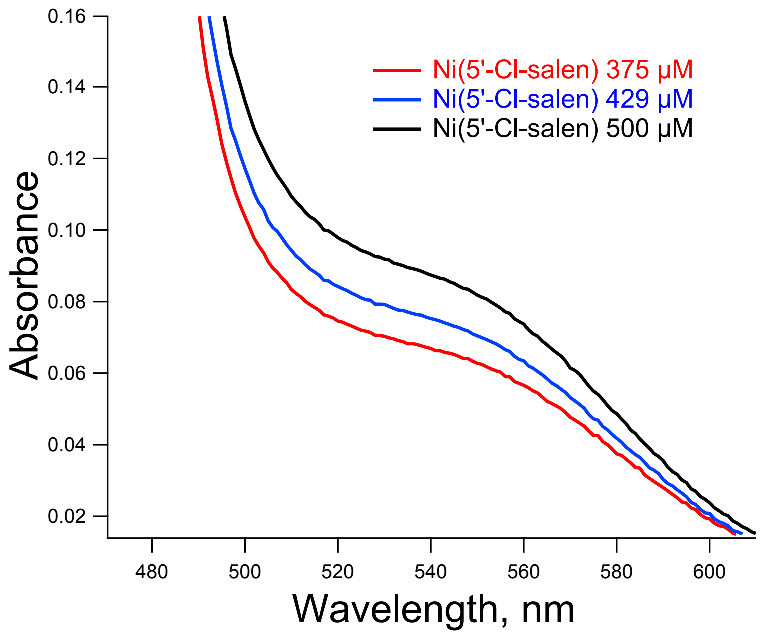

Figure S23. UV-vis spectrum of **Ni(5'-Cl-salen)**, highlighting the d→d absorption band, 375-500 µM in N,N-dimethylformamide.

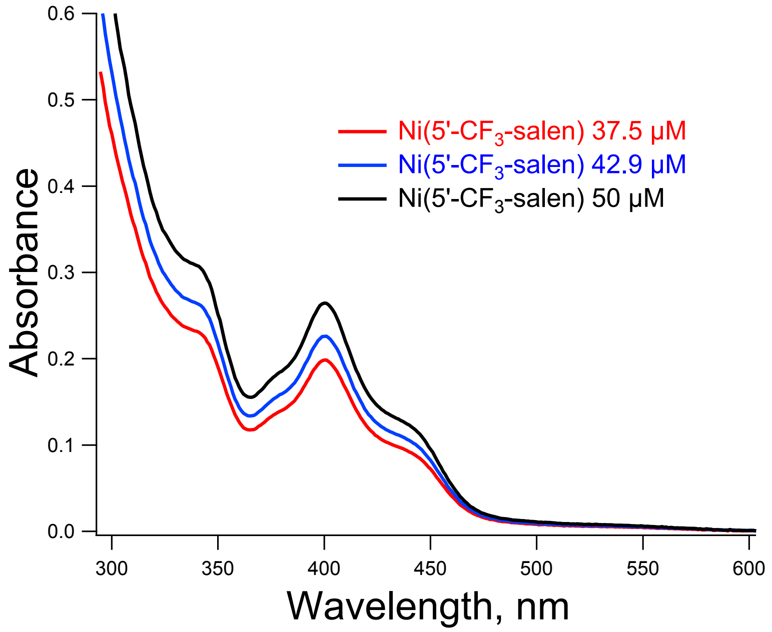

Figure S24. UV-vis spectrum of **Ni(5'-CF_3-salen)**, 37.5-50.0 µM in *N,N*-dimethylformamide.

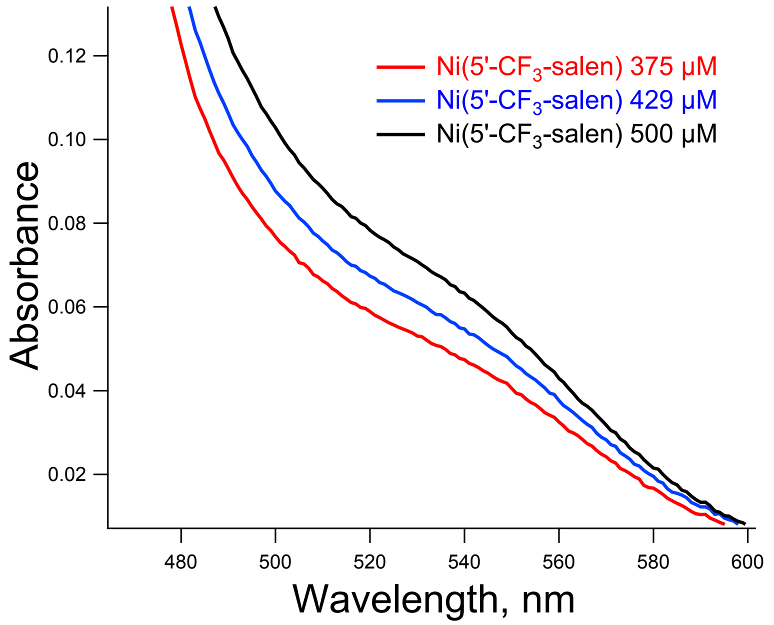

Figure S25. UV-vis spectrum of **Ni(5’-CF_3-salen)**, highlighting the d→d absorption band, 375-500 µM in N,N-dimethylformamide.

Cyclic Voltammetry

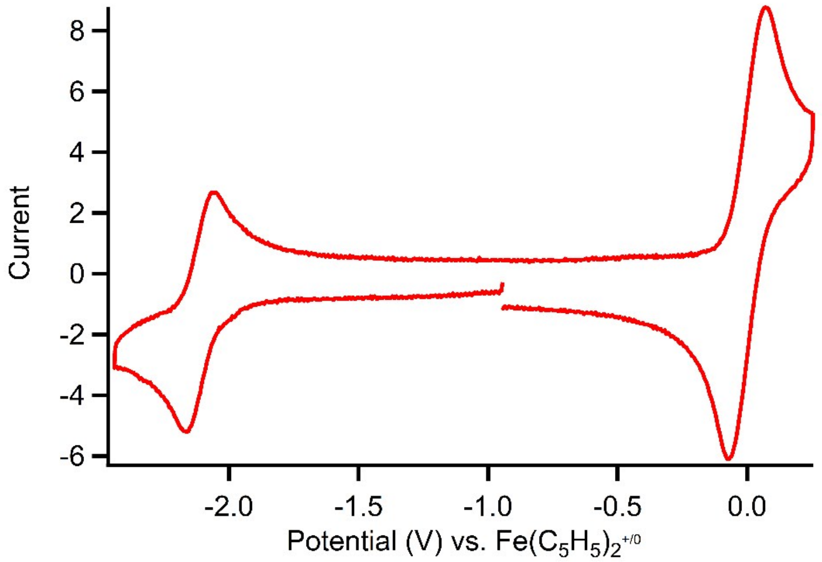

Figure S26. Cyclic voltammogram of **Ni(3’-OCH_3-salen)** in dimethylformamide. Current is given in µA.

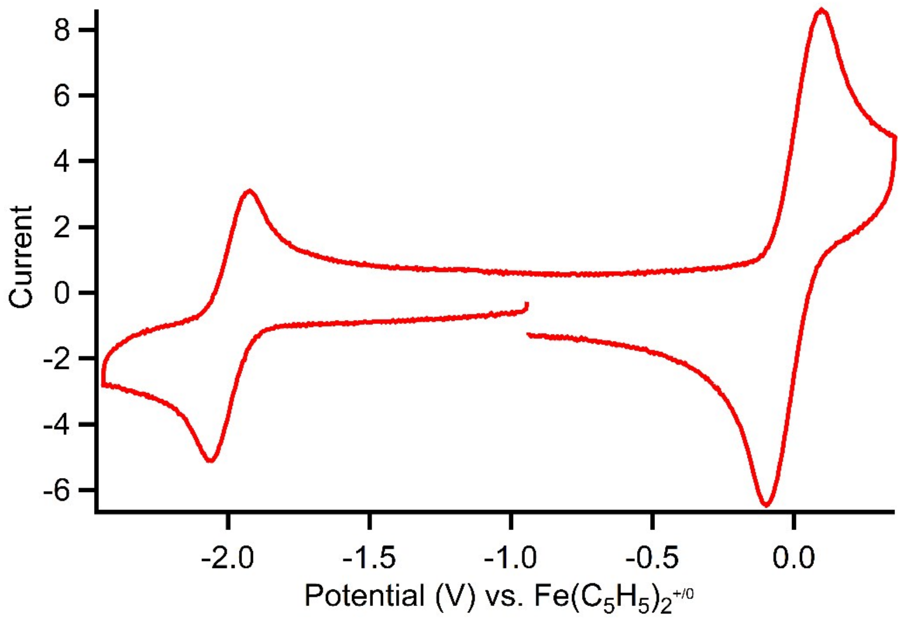

Figure S27. Cyclic voltammogram of **2Na(OTf)** in dimethylformamide. Current is given in μA.

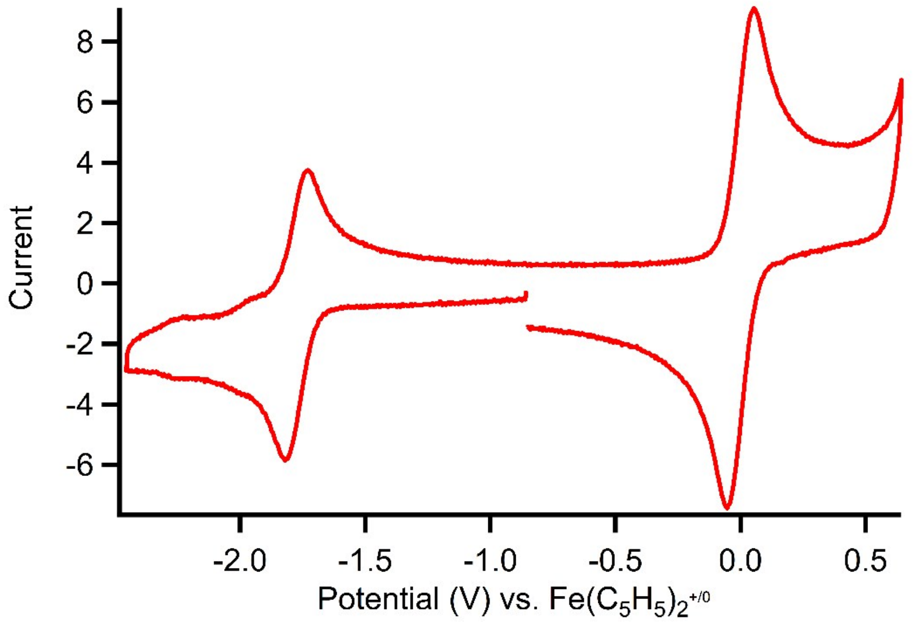

Figure S28. Cyclic voltammogram of **$2Ba(OTf)_2$** in dimethylformamide. Current is given in μA.

Infrared Spectra

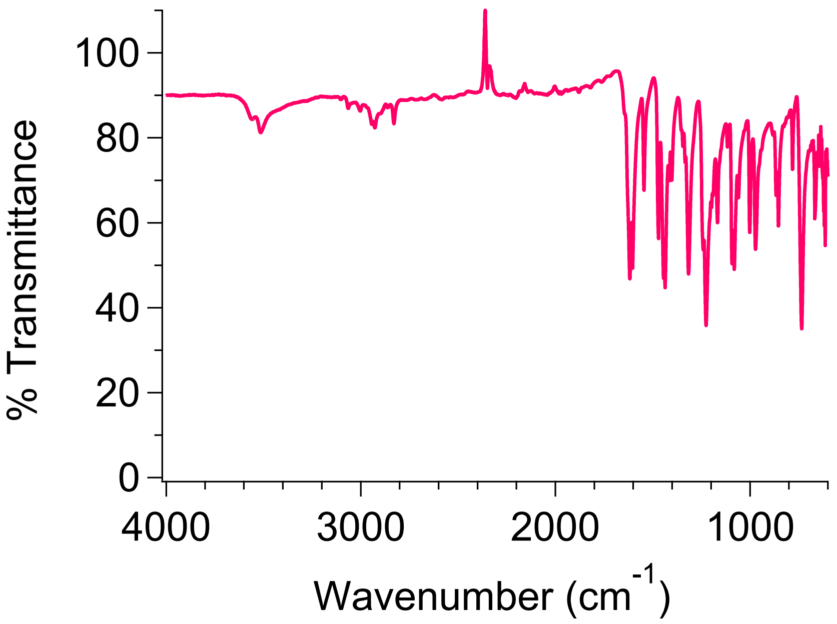

Figure S29. Solid state infrared spectrum of **Ni(3'-OCH$_3$-salen)**.

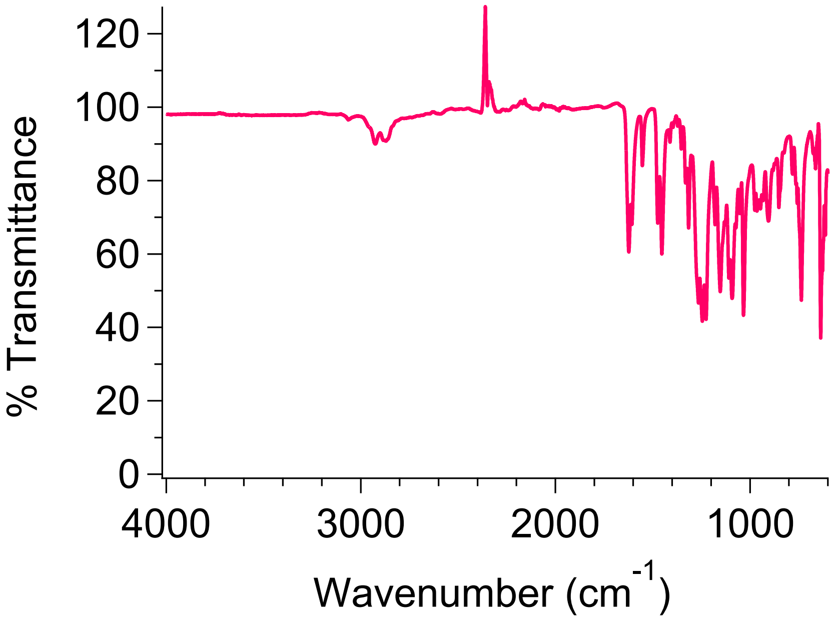

Figure S30. Solid state infrared spectrum of **2Na(OTf).**

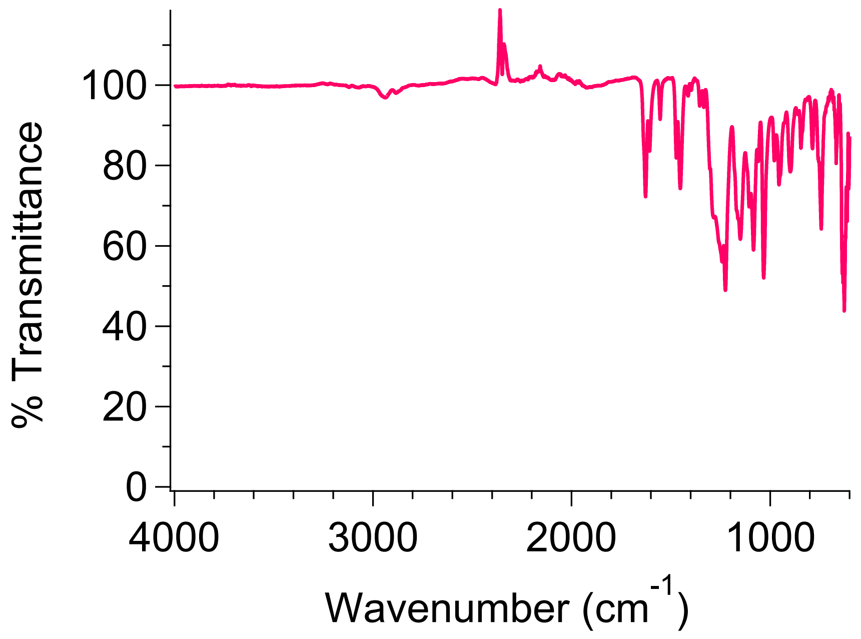

Figure S31. Solid state infrared spectrum of **2Ba(OTf)$_2$**.

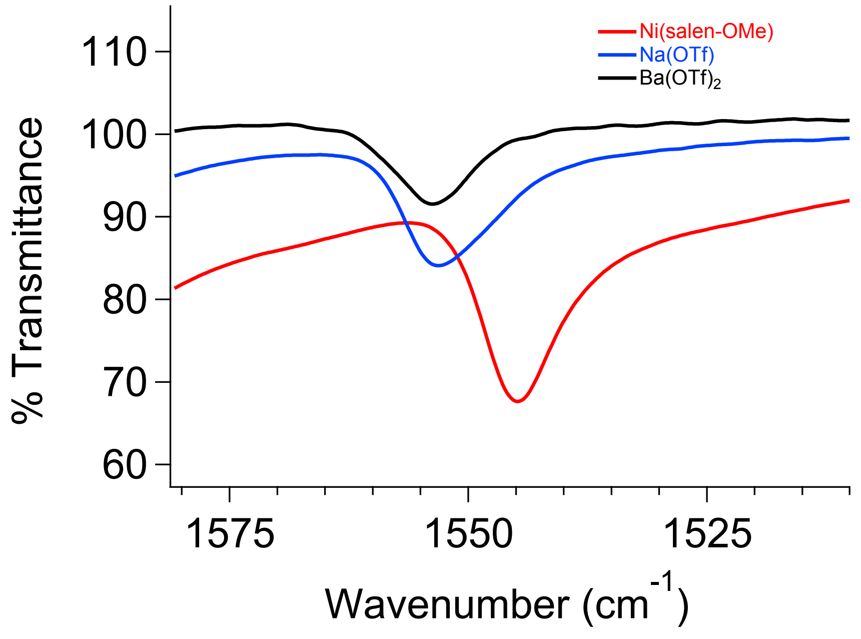

Figure S32. Overlay of the C=N vibrational stretches of **Ni(3'-OCH$_3$-salen)** and **2M** complexes.

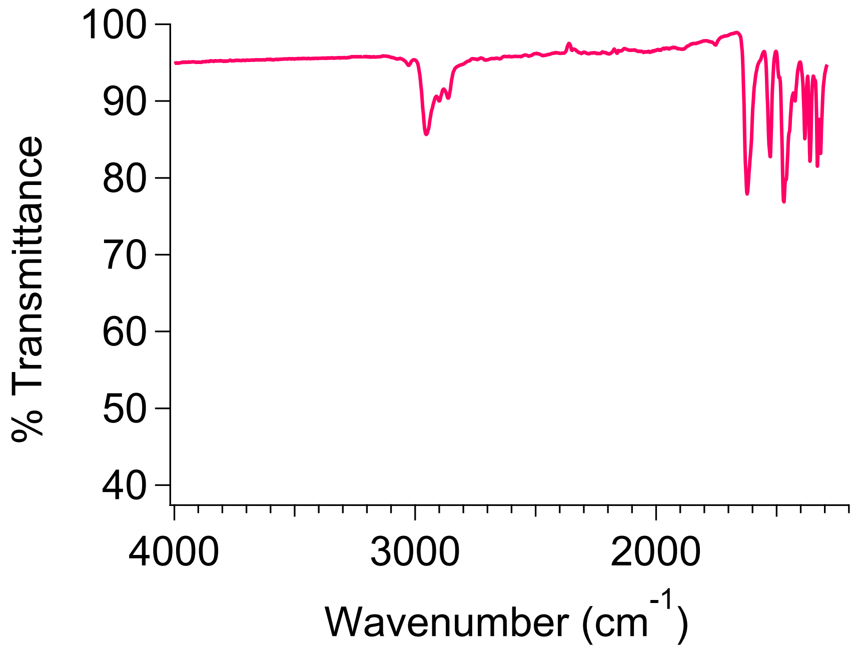

Figure S33. Solid state infrared spectrum of **Ni(5’-*t*Bu-salen)**.

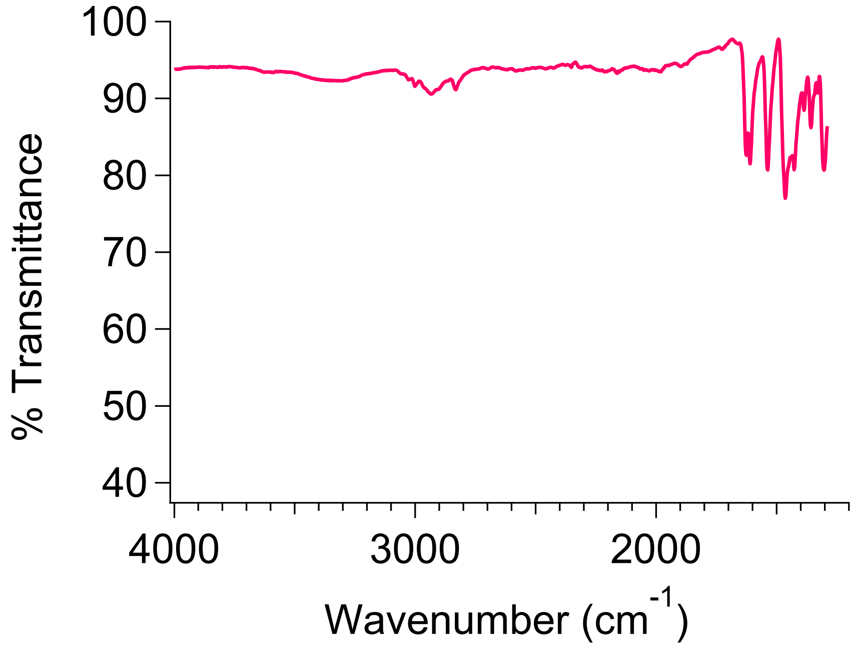

Figure S34. Solid state infrared spectrum of **Ni(5’-OCH$_3$-salen)**.

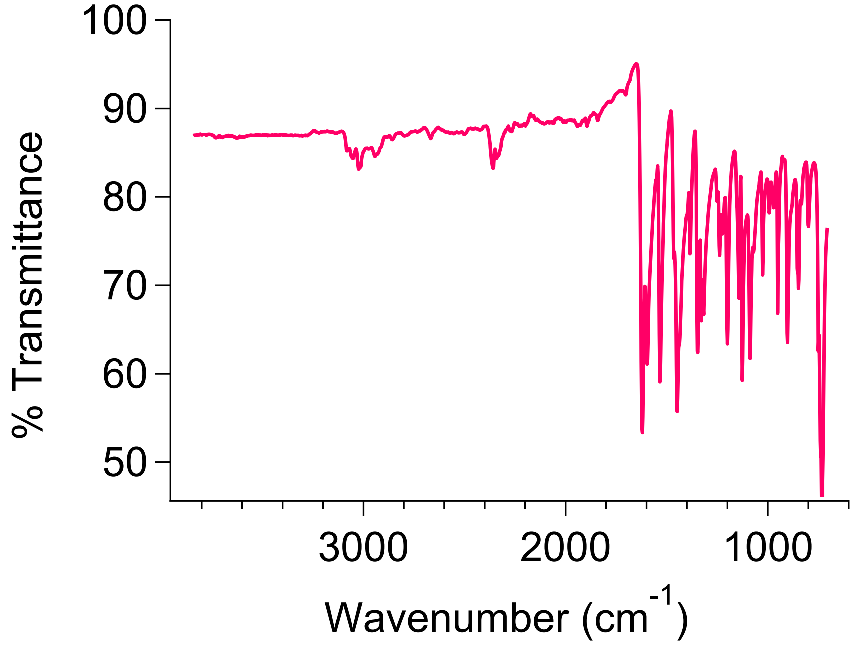

Figure S35. Solid state infrared spectrum of **Ni(salen)**.

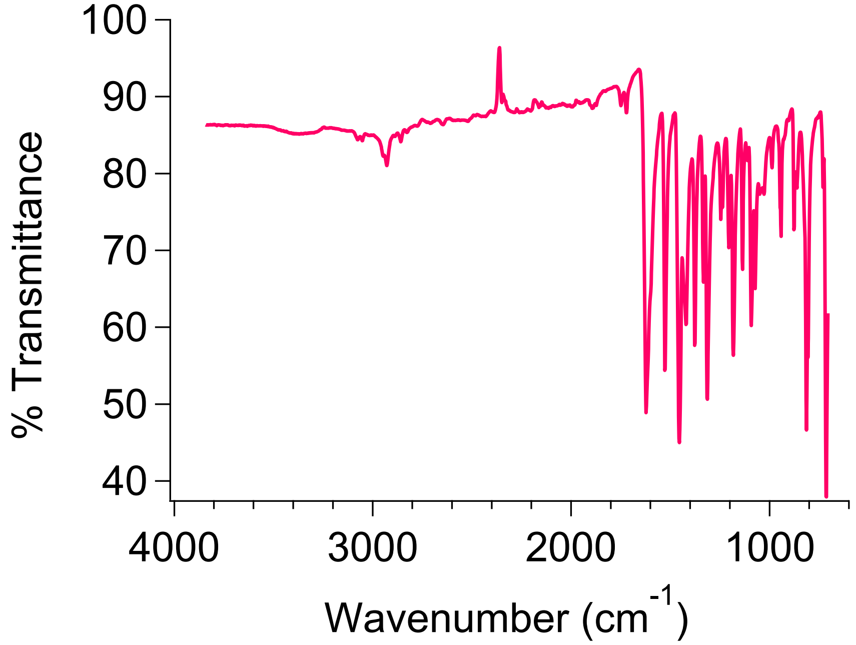

Figure S36. Solid state infrared spectrum of **Ni(5’-Cl-salen)**.

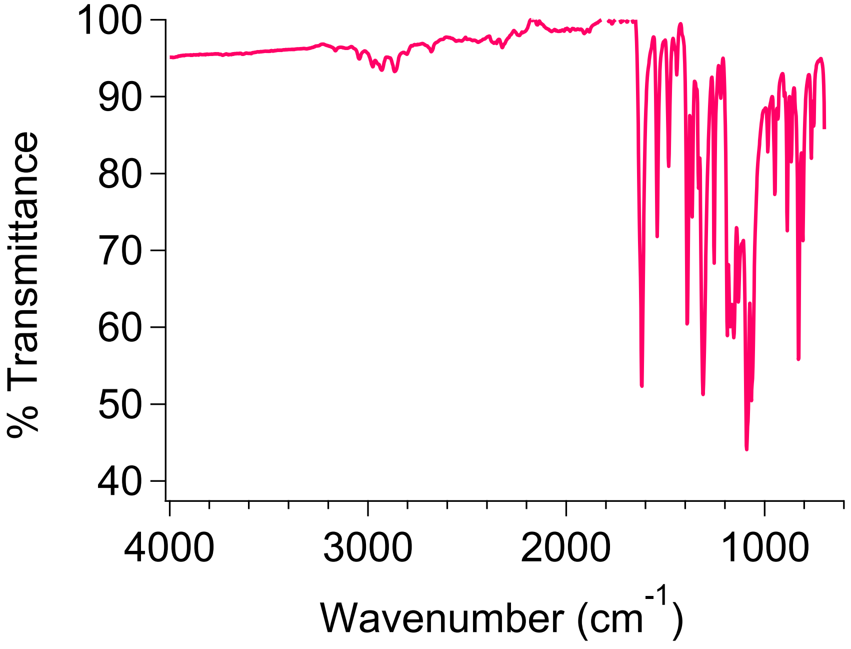

Figure S37. Solid state infrared spectrum of **Ni(5’-CF$_3$-salen)**.

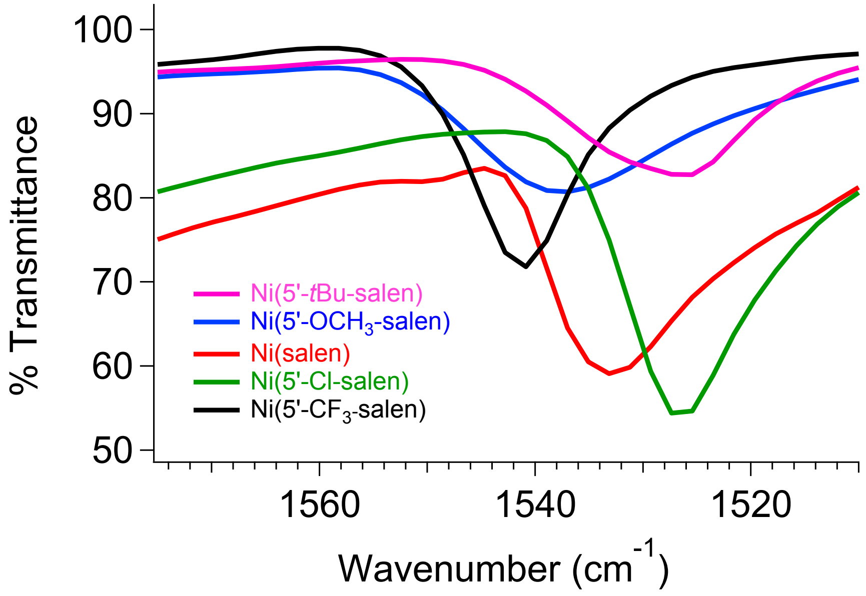

Figure S38. Overlay of the C=N vibrational stretches of **Ni(5’-R-salen)** complexes.

Crystallographic Data Tables

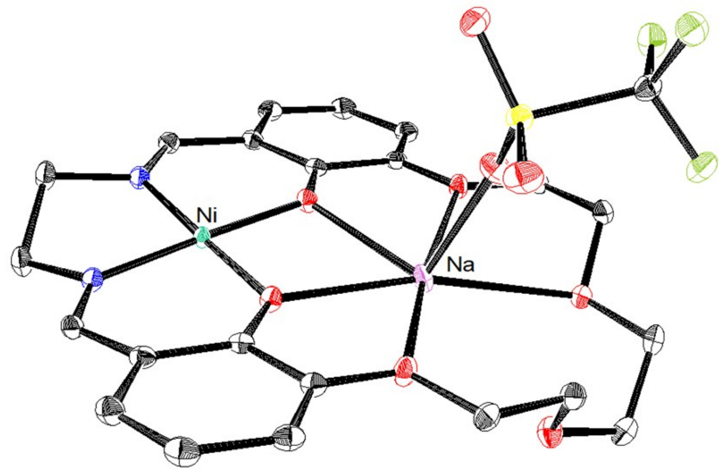

Figure S39. ORTEP of **2Na(OTf)**. Thermal ellipsoids are drawn to 50% probability. Hydrogen atoms and outersphere anions and solvent molecules have been omitted for clarity.

Table S1. Crystal data and structure refinement for **2Na(OTf)**.

Identification code	jyy166 (Kevin Kang)	
Empirical formula	$C_{23} H_{24} F_3 N_2 Na Ni O_9 S$	
Formula weight	643.20	
Temperature	88(2) K	
Wavelength	0.71073 Å	
Crystal system	Monoclinic	
Space group	$P2_1/n$	
Unit cell dimensions	a = 9.1543(8) Å	α= 90°.
	b = 16.514(2) Å	β= 101.501(2)°.
	c = 16.9255(16) Å	γ = 90°.
Volume	2507.3(4) Å^3	
Z	4	
Density (calculated)	1.704 Mg/m^3	

Absorption coefficient	0.955 mm^{-1}
F(000)	1320
Crystal color	violet
Crystal size	0.759 x 0.342 x 0.208 mm^3
Theta range for data collection	1.740 to 29.108°
Index ranges	$-12 \leq h \leq 12$, $-21 \leq k \leq 21$, $-21 \leq l \leq 22$
Reflections collected	30534
Independent reflections	6354 [R(int) = 0.0161]
Completeness to theta = 25.500°	100.0 %
Absorption correction	Semi-empirical from equivalents
Max. and min. transmission	0.7458 and 0.6594
Refinement method	Full-matrix least-squares on F^2
Data / restraints / parameters	6354 / 0 / 361
Goodness-of-fit on F^2	1.046
Final R indices [I>2sigma(I) = 5947 data]	R1 = 0.0233, wR2 = 0.0586
R indices (all data, 0.73 Å)	R1 = 0.0255, wR2 = 0.0599
Largest diff. peak and hole	0.559 and -0.358 e.$Å^{-3}$

Table S2. Atomic coordinates (x 10^4) and equivalent isotropic displacement parameters ($Å^2$x 10^3) for **2Na(OTf)**. U(eq) is defined as one third of the trace of the orthogonalized U^{ij} tensor.

	x	y	z	U(eq)
Ni(1)	2504(1)	3382(1)	5553(1)	9(1)
S(1)	2411(1)	3887(1)	8882(1)	14(1)
Na(1)	2398(1)	4887(1)	6943(1)	14(1)
O(1)	3824(1)	3889(1)	6372(1)	12(1)
O(2)	1068(1)	3984(1)	5919(1)	12(1)
O(3)	-423(1)	5080(1)	6522(1)	13(1)
O(4)	1145(1)	6286(1)	7417(1)	16(1)
O(5)	4345(1)	6251(1)	7754(1)	17(1)
O(6)	5174(1)	4684(1)	7621(1)	16(1)
O(7)	1977(1)	4348(1)	8147(1)	21(1)
O(8)	3979(1)	3910(1)	9245(1)	22(1)
O(9)	1701(1)	3104(1)	8880(1)	19(1)
N(1)	3968(1)	2809(1)	5181(1)	12(1)
N(2)	1160(1)	2860(1)	4755(1)	13(1)
F(1)	71(1)	4532(1)	9311(1)	21(1)
F(2)	2102(1)	5221(1)	9691(1)	22(1)
F(3)	1753(1)	4115(1)	10311(1)	21(1)
C(1)	5230(1)	3693(1)	6637(1)	11(1)
C(2)	6014(1)	4105(1)	7335(1)	12(1)
C(3)	7481(1)	3921(1)	7662(1)	15(1)

C(4)	8251(1)	3328(1)	7305(1)	17(1)
C(5)	7542(1)	2941(1)	6618(1)	15(1)
C(6)	6029(1)	3116(1)	6273(1)	12(1)
C(7)	5358(1)	2736(1)	5526(1)	13(1)
C(8)	3429(1)	2427(1)	4388(1)	16(1)
C(9)	1824(1)	2183(1)	4374(1)	17(1)
C(10)	-236(1)	3034(1)	4508(1)	13(1)
C(11)	-1008(1)	3667(1)	4840(1)	12(1)
C(12)	-2498(1)	3848(1)	4454(1)	14(1)
C(13)	-3270(1)	4453(1)	4746(1)	16(1)
C(14)	-2599(1)	4884(1)	5444(1)	15(1)
C(15)	-1167(1)	4706(1)	5835(1)	12(1)
C(16)	-308(1)	4099(1)	5532(1)	11(1)
C(17)	-1212(1)	5710(1)	6839(1)	14(1)
C(18)	-229(1)	6015(1)	7602(1)	16(1)
C(19)	1983(2)	6749(1)	8065(1)	17(1)
C(20)	3489(2)	6948(1)	7874(1)	18(1)
C(21)	4806(1)	5778(1)	8466(1)	15(1)
C(22)	5909(1)	5157(1)	8296(1)	15(1)
C(23)	1547(1)	4469(1)	9585(1)	15(1)

Table S3. Bond lengths [Å] and angles [°] for **2Na(OTf)**.

Ni(1)-O(1)	1.8475(8)
Ni(1)-N(2)	1.8488(10)
Ni(1)-O(2)	1.8506(8)
Ni(1)-N(1)	1.8506(10)
Ni(1)-Na(1)	3.4374(6)
S(1)-O(8)	1.4448(10)
S(1)-O(7)	1.4448(10)
S(1)-O(9)	1.4474(10)
S(1)-C(23)	1.8283(13)
Na(1)-O(7)	2.3243(11)
Na(1)-O(1)	2.4209(10)
Na(1)-O(2)	2.4225(10)
Na(1)-O(3)	2.5580(10)
Na(1)-O(6)	2.5936(10)
Na(1)-O(4)	2.7680(11)
O(1)-C(1)	1.3159(14)
O(2)-C(16)	1.3124(14)
O(3)-C(15)	1.3720(14)
O(3)-C(17)	1.4317(14)
O(4)-C(18)	1.4271(15)
O(4)-C(19)	1.4290(15)
O(5)-C(21)	1.4269(15)
O(5)-C(20)	1.4291(16)

O(6)-C(2)	1.3745(15)
O(6)-C(22)	1.4341(14)
N(1)-C(7)	1.2958(16)
N(1)-C(8)	1.4763(15)
N(2)-C(10)	1.2955(16)
N(2)-C(9)	1.4801(15)
F(1)-C(23)	1.3425(14)
F(2)-C(23)	1.3397(14)
F(3)-C(23)	1.3404(14)
C(1)-C(6)	1.4147(16)
C(1)-C(2)	1.4274(16)
C(2)-C(3)	1.3803(16)
C(3)-C(4)	1.4107(18)
C(4)-C(5)	1.3715(18)
C(5)-C(6)	1.4207(16)
C(6)-C(7)	1.4356(17)
C(8)-C(9)	1.5194(17)
C(10)-C(11)	1.4376(17)
C(11)-C(16)	1.4112(16)
C(11)-C(12)	1.4217(16)
C(12)-C(13)	1.3715(18)
C(13)-C(14)	1.4105(18)
C(14)-C(15)	1.3777(16)
C(15)-C(16)	1.4307(16)
C(17)-C(18)	1.5048(18)

C(19)-C(20)	1.5133(19)
C(21)-C(22)	1.5067(18)

O(1)-Ni(1)-N(2)	178.36(4)
O(1)-Ni(1)-O(2)	84.68(4)
N(2)-Ni(1)-O(2)	94.67(4)
O(1)-Ni(1)-N(1)	94.42(4)
N(2)-Ni(1)-N(1)	86.27(5)
O(2)-Ni(1)-N(1)	178.23(4)
O(1)-Ni(1)-Na(1)	42.47(3)
N(2)-Ni(1)-Na(1)	137.03(3)
O(2)-Ni(1)-Na(1)	42.55(3)
N(1)-Ni(1)-Na(1)	136.36(3)
O(8)-S(1)-O(7)	115.56(6)
O(8)-S(1)-O(9)	115.58(6)
O(7)-S(1)-O(9)	115.06(6)
O(8)-S(1)-C(23)	103.36(6)
O(7)-S(1)-C(23)	101.72(6)
O(9)-S(1)-C(23)	102.63(6)
O(7)-Na(1)-O(1)	106.65(4)
O(7)-Na(1)-O(2)	104.14(4)
O(1)-Na(1)-O(2)	61.90(3)
O(7)-Na(1)-O(3)	87.55(4)
O(1)-Na(1)-O(3)	124.62(3)
O(2)-Na(1)-O(3)	62.74(3)

O(7)-Na(1)-O(6)	83.21(4)
O(1)-Na(1)-O(6)	61.98(3)
O(2)-Na(1)-O(6)	123.07(3)
O(3)-Na(1)-O(6)	170.10(4)
O(7)-Na(1)-O(4)	85.11(4)
O(1)-Na(1)-O(4)	166.32(4)
O(2)-Na(1)-O(4)	122.87(3)
O(3)-Na(1)-O(4)	61.52(3)
O(6)-Na(1)-O(4)	113.93(3)
O(7)-Na(1)-Ni(1)	110.83(3)
O(1)-Na(1)-Ni(1)	31.02(2)
O(2)-Na(1)-Ni(1)	31.10(2)
O(3)-Na(1)-Ni(1)	93.67(2)
O(6)-Na(1)-Ni(1)	92.92(2)
O(4)-Na(1)-Ni(1)	150.65(3)
C(1)-O(1)-Ni(1)	126.41(8)
C(1)-O(1)-Na(1)	126.82(7)
Ni(1)-O(1)-Na(1)	106.51(4)
C(16)-O(2)-Ni(1)	125.84(8)
C(16)-O(2)-Na(1)	124.30(7)
Ni(1)-O(2)-Na(1)	106.34(4)
C(15)-O(3)-C(17)	116.29(9)
C(15)-O(3)-Na(1)	118.88(7)
C(17)-O(3)-Na(1)	122.90(7)
C(18)-O(4)-C(19)	111.18(9)

C(18)-O(4)-Na(1)	103.67(7)
C(19)-O(4)-Na(1)	118.61(7)
C(21)-O(5)-C(20)	113.48(10)
C(2)-O(6)-C(22)	116.94(9)
C(2)-O(6)-Na(1)	120.07(7)
C(22)-O(6)-Na(1)	122.99(7)
S(1)-O(7)-Na(1)	153.00(6)
C(7)-N(1)-C(8)	119.37(10)
C(7)-N(1)-Ni(1)	127.37(9)
C(8)-N(1)-Ni(1)	113.23(8)
C(10)-N(2)-C(9)	119.44(10)
C(10)-N(2)-Ni(1)	127.05(9)
C(9)-N(2)-Ni(1)	113.50(8)
O(1)-C(1)-C(6)	124.93(11)
O(1)-C(1)-C(2)	117.50(11)
C(6)-C(1)-C(2)	117.56(10)
O(6)-C(2)-C(3)	125.43(11)
O(6)-C(2)-C(1)	113.55(10)
C(3)-C(2)-C(1)	121.01(11)
C(2)-C(3)-C(4)	120.71(11)
C(5)-C(4)-C(3)	119.60(11)
C(4)-C(5)-C(6)	120.73(12)
C(1)-C(6)-C(5)	120.33(11)
C(1)-C(6)-C(7)	120.66(10)
C(5)-C(6)-C(7)	118.90(11)

N(1)-C(7)-C(6)	124.46(11)
N(1)-C(8)-C(9)	105.90(10)
N(2)-C(9)-C(8)	105.88(10)
N(2)-C(10)-C(11)	124.40(11)
C(16)-C(11)-C(12)	120.72(11)
C(16)-C(11)-C(10)	121.00(11)
C(12)-C(11)-C(10)	118.29(11)
C(13)-C(12)-C(11)	120.21(11)
C(12)-C(13)-C(14)	120.01(11)
C(15)-C(14)-C(13)	120.54(11)
O(3)-C(15)-C(14)	125.12(11)
O(3)-C(15)-C(16)	113.81(10)
C(14)-C(15)-C(16)	121.06(11)
O(2)-C(16)-C(11)	124.96(11)
O(2)-C(16)-C(15)	117.63(10)
C(11)-C(16)-C(15)	117.40(10)
O(3)-C(17)-C(18)	107.72(10)
O(4)-C(18)-C(17)	108.63(10)
O(4)-C(19)-C(20)	108.96(10)
O(5)-C(20)-C(19)	113.84(11)
O(5)-C(21)-C(22)	107.93(10)
O(6)-C(22)-C(21)	107.28(10)
F(2)-C(23)-F(3)	107.57(10)
F(2)-C(23)-F(1)	107.69(10)
F(3)-C(23)-F(1)	107.35(10)

F(2)-C(23)-S(1)	111.87(9)
F(3)-C(23)-S(1)	111.58(9)
F(1)-C(23)-S(1)	110.58(8)

Table S4. Anisotropic displacement parameters ($Å^2$x 10^3) for **2Na(OTf)**. The anisotropic displacement factor exponent takes the form: $-2\pi^2[h^2 a^{*2}U^{11} + ... + 2 h k a^* b^* U^{12}]$

	U^{11}	U^{22}	U^{33}	U^{23}	U^{13}	U^{12}
Ni(1)	8(1)	10(1)	10(1)	-1(1)	1(1)	1(1)
S(1)	13(1)	14(1)	16(1)	0(1)	3(1)	0(1)
Na(1)	10(1)	19(1)	10(1)	-3(1)	1(1)	3(1)
O(1)	9(1)	12(1)	14(1)	-2(1)	0(1)	2(1)
O(2)	9(1)	14(1)	12(1)	-2(1)	1(1)	2(1)
O(3)	12(1)	13(1)	15(1)	-3(1)	3(1)	2(1)
O(4)	18(1)	17(1)	15(1)	-5(1)	5(1)	-4(1)
O(5)	21(1)	18(1)	14(1)	0(1)	3(1)	2(1)
O(6)	12(1)	17(1)	15(1)	-7(1)	-1(1)	0(1)
O(7)	21(1)	25(1)	16(1)	4(1)	6(1)	1(1)
O(8)	12(1)	23(1)	31(1)	2(1)	2(1)	1(1)
O(9)	20(1)	14(1)	24(1)	-3(1)	6(1)	-2(1)
N(1)	12(1)	11(1)	13(1)	-2(1)	2(1)	1(1)
N(2)	13(1)	12(1)	13(1)	-2(1)	3(1)	0(1)
F(1)	14(1)	27(1)	21(1)	-5(1)	2(1)	2(1)
F(2)	28(1)	14(1)	24(1)	-4(1)	5(1)	-6(1)
F(3)	27(1)	21(1)	13(1)	1(1)	2(1)	-2(1)
C(1)	9(1)	11(1)	12(1)	3(1)	2(1)	0(1)
C(2)	12(1)	12(1)	13(1)	1(1)	2(1)	0(1)
C(3)	12(1)	17(1)	14(1)	2(1)	0(1)	-2(1)

C(4)	10(1)	20(1)	20(1)	3(1)	0(1)	2(1)
C(5)	11(1)	15(1)	19(1)	2(1)	3(1)	3(1)
C(6)	10(1)	12(1)	15(1)	2(1)	2(1)	0(1)
C(7)	12(1)	11(1)	17(1)	0(1)	5(1)	2(1)
C(8)	13(1)	20(1)	15(1)	-7(1)	2(1)	3(1)
C(9)	14(1)	17(1)	19(1)	-8(1)	1(1)	2(1)
C(10)	12(1)	14(1)	12(1)	-1(1)	2(1)	-2(1)
C(11)	9(1)	14(1)	13(1)	1(1)	3(1)	0(1)
C(12)	10(1)	17(1)	15(1)	1(1)	0(1)	-2(1)
C(13)	9(1)	18(1)	21(1)	2(1)	1(1)	0(1)
C(14)	11(1)	14(1)	21(1)	2(1)	5(1)	2(1)
C(15)	11(1)	11(1)	14(1)	1(1)	4(1)	-1(1)
C(16)	9(1)	11(1)	12(1)	3(1)	3(1)	0(1)
C(17)	14(1)	12(1)	18(1)	-1(1)	7(1)	3(1)
C(18)	19(1)	15(1)	18(1)	-2(1)	9(1)	0(1)
C(19)	21(1)	15(1)	16(1)	-5(1)	2(1)	1(1)
C(20)	21(1)	14(1)	19(1)	-3(1)	1(1)	-1(1)
C(21)	16(1)	17(1)	12(1)	-1(1)	2(1)	-2(1)
C(22)	14(1)	18(1)	13(1)	-4(1)	0(1)	-3(1)
C(23)	15(1)	14(1)	14(1)	-1(1)	0(1)	-2(1)

Table S5. Hydrogen coordinates (x 10^4) and isotropic displacement parameters ($Å^2$x 10^3) for **2Na(OTf)**.

	x	y	z	U(eq)
H(3A)	7977	4196	8132	18
H(4A)	9255	3198	7539	20
H(5A)	8068	2552	6369	18
H(7A)	5976	2410	5267	15
H(8A)	4039	1946	4322	20
H(8B)	3481	2814	3947	20
H(9A)	1280	2100	3813	20
H(9B)	1786	1675	4680	20
H(10A)	-788	2723	4077	15
H(12A)	-2961	3548	3993	17
H(13A)	-4255	4582	4478	20
H(14A)	-3141	5300	5647	18
H(17A)	-2159	5499	6956	17
H(17B)	-1448	6156	6443	17
H(18A)	-726	6467	7828	20
H(18B)	-40	5576	8007	20
H(19A)	2115	6435	8573	21
H(19B)	1444	7255	8136	21
H(20A)	3338	7286	7382	22
H(20B)	4064	7272	8323	22

H(21A)	3935	5506	8614	18
H(21B)	5275	6130	8920	18
H(22A)	6798	5428	8167	19
H(22B)	6233	4806	8772	19

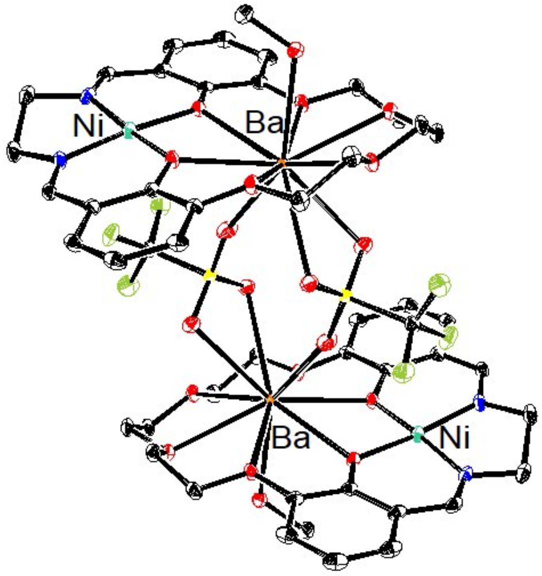

Figure S40. ORTEP of **2Ba(OTf)₂**. Thermal ellipsoids are drawn to 50% probability. Hydrogen atoms and outersphere anions and solvent molecules have been omitted for clarity.

Table S6. Crystal data and structure refinement for **2Ba(OTf)₂**.

Identification code	jyy150 (Kevin Kang)	
Empirical formula	C_{50} H_{56} Ba_2 F_{12} N_4 Ni_2 O_{26} S_4	
Formula weight	1877.32	
Temperature	88(2) K	
Wavelength	0.71073 Å	
Crystal system	Triclinic	
Space group	$P\bar{1}$	
Unit cell dimensions	a = 10.0313(10) Å	α= 80.3583(10)°.
	b = 12.0323(12) Å	β= 74.2611(11)°.
	c = 14.3201(14) Å	γ = 79.4090(11)°.
Volume	1622.6(3) Å^3	
Z	1	

Density (calculated)	1.921 Mg/m^3
Absorption coefficient	2.016 mm^{-1}
F(000)	932
Crystal color	red
Crystal size	0.367 x 0.366 x 0.204 mm^3
Theta range for data collection	1.735 to 29.076°
Index ranges	$-13 \le h \le 13$, $-16 \le k \le 16$, $-19 \le l \le 19$
Reflections collected	20047
Independent reflections	7985 [R(int) = 0.0205]
Completeness to theta = 25.500°	99.8 %
Absorption correction	Semi-empirical from equivalents
Max. and min. transmission	0.4318 and 0.3734
Refinement method	Full-matrix least-squares on F^2
Data / restraints / parameters	7985 / 0 / 456
Goodness-of-fit on F^2	1.054
Final R indices [I>2sigma(I) = 7701 data]	R1 = 0.0219, wR2 = 0.0589
R indices (all data, 0.73 Å)	R1 = 0.0228, wR2 = 0.0594
Largest diff. peak and hole	0.676 and -1.380 e.$Å^{-3}$

Table S7. Atomic coordinates (x 10^4) and equivalent isotropic displacement parameters ($Å^2$x 10^3) for **2Ba(OTf)$_2$**. U(eq) is defined as one third of the trace of the orthogonalized U^{ij} tensor.

	x	y	z	U(eq)
Ba(1)	7793(1)	6010(1)	6879(1)	9(1)
Ni(1)	7255(1)	3192(1)	8316(1)	14(1)
S(1)	10465(1)	6574(1)	4862(1)	13(1)
S(2)	7555(1)	743(1)	11778(1)	14(1)
F(1)	10537(2)	8723(1)	4959(1)	29(1)
F(2)	12551(1)	7763(1)	4400(1)	29(1)
F(3)	11094(2)	8272(1)	3497(1)	25(1)
F(4)	8389(2)	657(1)	13383(1)	32(1)
F(5)	8461(2)	-984(1)	12942(1)	26(1)
F(6)	6480(2)	-48(1)	13608(1)	35(1)
O(1)	8351(2)	4308(1)	8268(1)	14(1)
O(2)	6235(1)	4290(1)	7583(1)	12(1)
O(3)	5395(2)	5954(1)	6287(1)	15(1)
O(4)	6115(2)	8052(1)	6281(1)	15(1)
O(5)	8342(2)	8156(1)	7096(1)	15(1)
O(6)	9390(2)	6170(1)	8160(1)	14(1)
O(7)	10678(2)	6273(1)	5839(1)	19(1)
O(8)	9002(2)	6889(1)	4857(1)	17(1)
O(9)	8720(2)	4175(1)	5840(1)	28(1)
O(10)	8985(2)	643(1)	11204(1)	20(1)

O(11)	6916(2)	1897(1)	11947(1)	23(1)
O(12)	6673(2)	76(2)	11530(1)	29(1)
O(13)	5651(2)	6943(1)	8347(1)	18(1)
N(1)	8396(2)	2054(1)	8906(1)	15(1)
N(2)	6065(2)	2130(1)	8495(1)	16(1)
C(1)	9477(2)	4198(2)	8610(1)	12(1)
C(2)	10107(2)	5184(2)	8540(1)	14(1)
C(3)	11320(2)	5125(2)	8842(2)	17(1)
C(4)	11927(2)	4078(2)	9261(2)	20(1)
C(5)	11331(2)	3120(2)	9357(2)	18(1)
C(6)	10102(2)	3159(2)	9035(1)	15(1)
C(7)	9527(2)	2126(2)	9140(1)	16(1)
C(8)	7965(2)	923(2)	9006(2)	20(1)
C(9)	6392(2)	1126(2)	9186(2)	21(1)
C(10)	5044(2)	2183(2)	8097(2)	16(1)
C(11)	4591(2)	3125(2)	7444(2)	15(1)
C(12)	3444(2)	3042(2)	7078(2)	19(1)
C(13)	2916(2)	3933(2)	6488(2)	21(1)
C(14)	3553(2)	4924(2)	6216(2)	19(1)
C(15)	4694(2)	5015(2)	6556(2)	14(1)
C(16)	5218(2)	4128(2)	7213(1)	13(1)
C(17)	4708(2)	6952(2)	5798(2)	17(1)
C(18)	5682(2)	7835(2)	5462(2)	17(1)
C(19)	6846(2)	9016(2)	6056(2)	18(1)
C(20)	7312(2)	9120(2)	6944(2)	18(1)

C(21)	8810(2)	8185(2)	7948(2)	18(1)
C(22)	9962(2)	7213(2)	8044(2)	16(1)
C(23)	11197(2)	7905(2)	4409(2)	16(1)
C(24)	7730(2)	59(2)	12989(2)	18(1)
C(25)	5064(3)	6198(2)	9179(2)	24(1)

Table S8. Bond lengths [Å] and angles [°] for **2Ba(OTf)2**.

Ba(1)-O(1)	2.6983(14)
Ba(1)-O(2)	2.7166(13)
Ba(1)-O(9)	2.7482(16)
Ba(1)-O(3)	2.7742(14)
Ba(1)-O(13)	2.7838(16)
Ba(1)-O(6)	2.7920(14)
Ba(1)-O(5)	2.8185(14)
Ba(1)-O(4)	2.8567(14)
Ba(1)-O(8)	2.9175(15)
Ba(1)-O(7)	2.9207(15)
Ba(1)-S(1)	3.4343(5)
Ba(1)-Ni(1)	3.7095(4)
Ni(1)-N(2)	1.8459(17)
Ni(1)-N(1)	1.8602(17)
Ni(1)-O(1)	1.8678(14)
Ni(1)-O(2)	1.8756(14)
S(1)-O(9)#1	1.4412(16)
S(1)-O(7)	1.4484(15)
S(1)-O(8)	1.4486(15)
S(1)-C(23)	1.831(2)
S(2)-O(10)	1.4418(16)
S(2)-O(12)	1.4426(16)
S(2)-O(11)	1.4487(16)

S(2)-C(24)	1.830(2)
F(1)-C(23)	1.328(2)
F(2)-C(23)	1.334(2)
F(3)-C(23)	1.333(2)
F(4)-C(24)	1.336(3)
F(5)-C(24)	1.333(2)
F(6)-C(24)	1.339(3)
O(1)-C(1)	1.325(2)
O(2)-C(16)	1.327(2)
O(3)-C(15)	1.386(2)
O(3)-C(17)	1.443(2)
O(4)-C(19)	1.430(2)
O(4)-C(18)	1.433(2)
O(5)-C(21)	1.429(2)
O(5)-C(20)	1.435(2)
O(6)-C(2)	1.380(2)
O(6)-C(22)	1.440(2)
O(9)-S(1)#1	1.4412(16)
O(13)-C(25)	1.423(3)
N(1)-C(7)	1.288(3)
N(1)-C(8)	1.475(3)
N(2)-C(10)	1.288(3)
N(2)-C(9)	1.475(3)
C(1)-C(6)	1.411(3)
C(1)-C(2)	1.421(3)

C(2)-C(3)	1.383(3)
C(3)-C(4)	1.407(3)
C(4)-C(5)	1.364(3)
C(5)-C(6)	1.418(3)
C(6)-C(7)	1.433(3)
C(8)-C(9)	1.509(3)
C(10)-C(11)	1.432(3)
C(11)-C(16)	1.412(3)
C(11)-C(12)	1.413(3)
C(12)-C(13)	1.369(3)
C(13)-C(14)	1.404(3)
C(14)-C(15)	1.387(3)
C(15)-C(16)	1.423(3)
C(17)-C(18)	1.505(3)
C(19)-C(20)	1.500(3)
C(21)-C(22)	1.503(3)

O(1)-Ba(1)-O(2)	56.76(4)
O(1)-Ba(1)-O(9)	77.89(5)
O(2)-Ba(1)-O(9)	66.92(5)
O(1)-Ba(1)-O(3)	114.91(4)
O(2)-Ba(1)-O(3)	58.25(4)
O(9)-Ba(1)-O(3)	80.74(5)
O(1)-Ba(1)-O(13)	86.81(4)
O(2)-Ba(1)-O(13)	77.53(4)

O(9)-Ba(1)-O(13)	144.15(5)
O(3)-Ba(1)-O(13)	76.67(5)
O(1)-Ba(1)-O(6)	56.61(4)
O(2)-Ba(1)-O(6)	110.55(4)
O(9)-Ba(1)-O(6)	114.60(5)
O(3)-Ba(1)-O(6)	157.21(4)
O(13)-Ba(1)-O(6)	81.55(5)
O(1)-Ba(1)-O(5)	114.92(4)
O(2)-Ba(1)-O(5)	148.00(4)
O(9)-Ba(1)-O(5)	144.87(5)
O(3)-Ba(1)-O(5)	117.49(4)
O(13)-Ba(1)-O(5)	70.95(4)
O(6)-Ba(1)-O(5)	59.96(4)
O(1)-Ba(1)-O(4)	150.61(4)
O(2)-Ba(1)-O(4)	110.90(4)
O(9)-Ba(1)-O(4)	124.49(5)
O(3)-Ba(1)-O(4)	58.42(4)
O(13)-Ba(1)-O(4)	63.90(4)
O(6)-Ba(1)-O(4)	116.94(4)
O(5)-Ba(1)-O(4)	59.49(4)
O(1)-Ba(1)-O(8)	140.03(4)
O(2)-Ba(1)-O(8)	129.35(4)
O(9)-Ba(1)-O(8)	72.75(5)
O(3)-Ba(1)-O(8)	86.63(4)
O(13)-Ba(1)-O(8)	132.30(4)

O(6)-Ba(1)-O(8)	113.40(4)
O(5)-Ba(1)-O(8)	78.41(4)
O(4)-Ba(1)-O(8)	69.24(4)
O(1)-Ba(1)-O(7)	97.98(4)
O(2)-Ba(1)-O(7)	137.80(4)
O(9)-Ba(1)-O(7)	75.45(5)
O(3)-Ba(1)-O(7)	133.90(4)
O(13)-Ba(1)-O(7)	139.38(5)
O(6)-Ba(1)-O(7)	68.39(4)
O(5)-Ba(1)-O(7)	70.53(4)
O(4)-Ba(1)-O(7)	105.72(4)
O(8)-Ba(1)-O(7)	48.86(4)
O(1)-Ba(1)-S(1)	117.98(3)
O(2)-Ba(1)-S(1)	135.42(3)
O(9)-Ba(1)-S(1)	68.83(4)
O(3)-Ba(1)-S(1)	109.47(3)
O(13)-Ba(1)-S(1)	145.47(3)
O(6)-Ba(1)-S(1)	92.30(3)
O(5)-Ba(1)-S(1)	76.56(3)
O(4)-Ba(1)-S(1)	89.77(3)
O(8)-Ba(1)-S(1)	24.69(3)
O(7)-Ba(1)-S(1)	24.69(3)
O(1)-Ba(1)-Ni(1)	28.74(3)
O(2)-Ba(1)-Ni(1)	29.03(3)
O(9)-Ba(1)-Ni(1)	64.53(4)

O(3)-Ba(1)-Ni(1)	87.05(3)
O(13)-Ba(1)-Ni(1)	86.69(3)
O(6)-Ba(1)-Ni(1)	85.07(3)
O(5)-Ba(1)-Ni(1)	140.21(3)
O(4)-Ba(1)-Ni(1)	137.89(3)
O(8)-Ba(1)-Ni(1)	137.28(3)
O(7)-Ba(1)-Ni(1)	115.97(3)
S(1)-Ba(1)-Ni(1)	126.822(9)
N(2)-Ni(1)-N(1)	86.70(8)
N(2)-Ni(1)-O(1)	173.86(7)
N(1)-Ni(1)-O(1)	93.26(7)
N(2)-Ni(1)-O(2)	93.88(7)
N(1)-Ni(1)-O(2)	173.35(7)
O(1)-Ni(1)-O(2)	86.87(6)
N(2)-Ni(1)-Ba(1)	137.95(6)
N(1)-Ni(1)-Ba(1)	133.51(6)
O(1)-Ni(1)-Ba(1)	44.00(4)
O(2)-Ni(1)-Ba(1)	44.66(4)
O(9)#1-S(1)-O(7)	115.35(10)
O(9)#1-S(1)-O(8)	115.47(10)
O(7)-S(1)-O(8)	112.91(9)
O(9)#1-S(1)-C(23)	102.86(10)
O(7)-S(1)-C(23)	104.45(9)
O(8)-S(1)-C(23)	103.80(9)
O(9)#1-S(1)-Ba(1)	128.65(7)

O(7)-S(1)-Ba(1)	57.38(6)
O(8)-S(1)-Ba(1)	57.26(6)
C(23)-S(1)-Ba(1)	128.49(7)
O(10)-S(2)-O(12)	115.52(11)
O(10)-S(2)-O(11)	114.48(10)
O(12)-S(2)-O(11)	114.83(11)
O(10)-S(2)-C(24)	102.68(10)
O(12)-S(2)-C(24)	103.57(10)
O(11)-S(2)-C(24)	103.36(10)
C(1)-O(1)-Ni(1)	127.80(12)
C(1)-O(1)-Ba(1)	122.32(12)
Ni(1)-O(1)-Ba(1)	107.26(6)
C(16)-O(2)-Ni(1)	126.69(12)
C(16)-O(2)-Ba(1)	123.80(12)
Ni(1)-O(2)-Ba(1)	106.31(6)
C(15)-O(3)-C(17)	116.21(15)
C(15)-O(3)-Ba(1)	121.66(11)
C(17)-O(3)-Ba(1)	121.61(11)
C(19)-O(4)-C(18)	112.41(15)
C(19)-O(4)-Ba(1)	112.18(11)
C(18)-O(4)-Ba(1)	106.59(11)
C(21)-O(5)-C(20)	111.43(15)
C(21)-O(5)-Ba(1)	115.06(11)
C(20)-O(5)-Ba(1)	116.98(11)
C(2)-O(6)-C(22)	117.80(15)

C(2)-O(6)-Ba(1)	118.62(11)
C(22)-O(6)-Ba(1)	117.21(11)
S(1)-O(7)-Ba(1)	97.93(7)
S(1)-O(8)-Ba(1)	98.06(7)
S(1)#1-O(9)-Ba(1)	162.28(10)
C(25)-O(13)-Ba(1)	118.22(13)
C(7)-N(1)-C(8)	118.99(17)
C(7)-N(1)-Ni(1)	128.60(14)
C(8)-N(1)-Ni(1)	112.09(13)
C(10)-N(2)-C(9)	119.81(17)
C(10)-N(2)-Ni(1)	127.91(15)
C(9)-N(2)-Ni(1)	112.28(14)
O(1)-C(1)-C(6)	124.25(18)
O(1)-C(1)-C(2)	118.22(17)
C(6)-C(1)-C(2)	117.53(18)
O(6)-C(2)-C(3)	124.82(18)
O(6)-C(2)-C(1)	113.68(16)
C(3)-C(2)-C(1)	121.50(18)
C(2)-C(3)-C(4)	119.9(2)
C(5)-C(4)-C(3)	120.02(19)
C(4)-C(5)-C(6)	120.94(19)
C(1)-C(6)-C(5)	120.09(19)
C(1)-C(6)-C(7)	121.09(18)
C(5)-C(6)-C(7)	118.81(18)
N(1)-C(7)-C(6)	124.83(18)

N(1)-C(8)-C(9)	106.09(17)
N(2)-C(9)-C(8)	105.76(17)
N(2)-C(10)-C(11)	125.19(18)
C(16)-C(11)-C(12)	120.89(19)
C(16)-C(11)-C(10)	121.33(18)
C(12)-C(11)-C(10)	117.69(18)
C(13)-C(12)-C(11)	120.7(2)
C(12)-C(13)-C(14)	119.62(19)
C(15)-C(14)-C(13)	120.6(2)
O(3)-C(15)-C(14)	123.19(19)
O(3)-C(15)-C(16)	115.70(16)
C(14)-C(15)-C(16)	121.11(19)
O(2)-C(16)-C(11)	123.91(18)
O(2)-C(16)-C(15)	119.08(17)
C(11)-C(16)-C(15)	117.01(17)
O(3)-C(17)-C(18)	108.92(16)
O(4)-C(18)-C(17)	109.26(16)
O(4)-C(19)-C(20)	107.55(16)
O(5)-C(20)-C(19)	108.40(16)
O(5)-C(21)-C(22)	110.12(16)
O(6)-C(22)-C(21)	107.54(16)
F(1)-C(23)-F(3)	107.89(17)
F(1)-C(23)-F(2)	107.87(17)
F(3)-C(23)-F(2)	107.34(17)
F(1)-C(23)-S(1)	111.57(14)

F(3)-C(23)-S(1)	110.82(14)
F(2)-C(23)-S(1)	111.17(14)
F(5)-C(24)-F(4)	107.54(17)
F(5)-C(24)-F(6)	107.32(17)
F(4)-C(24)-F(6)	107.56(19)
F(5)-C(24)-S(2)	111.20(15)
F(4)-C(24)-S(2)	111.45(14)
F(6)-C(24)-S(2)	111.56(15)

Symmetry transformations used to generate equivalent atoms:

#1 -x+2,-y+1,-z+1

Table S9. Anisotropic displacement parameters ($Å^2 x 10^3$) for **2Ba(OTf)₂**. The anisotropic displacement factor exponent takes the form: $-2\square^2[h^2 a^{*2}U^{11} + ... + 2 h k a^* b^* U^{12}]$

	U^{11}	U^{22}	U^{33}	U^{23}	U^{13}	U^{12}
Ba(1)	10(1)	9(1)	10(1)	-1(1)	-3(1)	-2(1)
Ni(1)	16(1)	11(1)	15(1)	-1(1)	-4(1)	-3(1)
S(1)	13(1)	11(1)	13(1)	-2(1)	-1(1)	-3(1)
S(2)	14(1)	14(1)	16(1)	1(1)	-5(1)	-4(1)
F(1)	35(1)	16(1)	35(1)	-12(1)	2(1)	-8(1)
F(2)	15(1)	31(1)	42(1)	7(1)	-12(1)	-10(1)
F(3)	31(1)	26(1)	20(1)	8(1)	-9(1)	-11(1)
F(4)	53(1)	29(1)	23(1)	0(1)	-19(1)	-16(1)
F(5)	27(1)	17(1)	34(1)	4(1)	-15(1)	0(1)
F(6)	26(1)	40(1)	26(1)	12(1)	4(1)	-4(1)
O(1)	14(1)	11(1)	17(1)	0(1)	-7(1)	-4(1)
O(2)	12(1)	12(1)	14(1)	0(1)	-6(1)	-4(1)
O(3)	14(1)	13(1)	19(1)	1(1)	-9(1)	-2(1)
O(4)	17(1)	13(1)	14(1)	1(1)	-5(1)	-3(1)
O(5)	18(1)	10(1)	17(1)	-2(1)	-6(1)	-1(1)
O(6)	16(1)	11(1)	18(1)	0(1)	-8(1)	-5(1)
O(7)	14(1)	23(1)	16(1)	3(1)	-4(1)	-2(1)
O(8)	15(1)	22(1)	17(1)	-1(1)	-6(1)	-7(1)
O(9)	33(1)	16(1)	26(1)	-10(1)	11(1)	-8(1)
O(10)	18(1)	22(1)	17(1)	-3(1)	0(1)	-2(1)

O(11)	20(1)	17(1)	27(1)	1(1)	-4(1)	2(1)
O(12)	33(1)	28(1)	33(1)	5(1)	-20(1)	-18(1)
O(13)	16(1)	19(1)	16(1)	2(1)	-2(1)	1(1)
N(1)	22(1)	9(1)	12(1)	-1(1)	-3(1)	0(1)
N(2)	20(1)	13(1)	14(1)	0(1)	-2(1)	-7(1)
C(1)	13(1)	13(1)	10(1)	-2(1)	-2(1)	0(1)
C(2)	13(1)	16(1)	11(1)	-3(1)	-3(1)	-1(1)
C(3)	14(1)	23(1)	16(1)	-6(1)	-4(1)	-3(1)
C(4)	16(1)	29(1)	17(1)	-6(1)	-7(1)	3(1)
C(5)	18(1)	22(1)	14(1)	-2(1)	-6(1)	4(1)
C(6)	16(1)	16(1)	11(1)	-2(1)	-3(1)	2(1)
C(7)	22(1)	13(1)	11(1)	0(1)	-1(1)	3(1)
C(8)	29(1)	10(1)	20(1)	-2(1)	-7(1)	-2(1)
C(9)	27(1)	15(1)	19(1)	2(1)	-4(1)	-8(1)
C(10)	17(1)	15(1)	18(1)	-5(1)	1(1)	-9(1)
C(11)	14(1)	17(1)	16(1)	-5(1)	-1(1)	-7(1)
C(12)	17(1)	24(1)	21(1)	-8(1)	-2(1)	-9(1)
C(13)	14(1)	31(1)	23(1)	-12(1)	-6(1)	-6(1)
C(14)	14(1)	25(1)	20(1)	-7(1)	-7(1)	-1(1)
C(15)	12(1)	17(1)	15(1)	-5(1)	-2(1)	-3(1)
C(16)	11(1)	16(1)	13(1)	-6(1)	-1(1)	-3(1)
C(17)	15(1)	17(1)	19(1)	1(1)	-9(1)	1(1)
C(18)	19(1)	17(1)	14(1)	1(1)	-7(1)	1(1)
C(19)	20(1)	12(1)	21(1)	2(1)	-6(1)	-3(1)
C(20)	20(1)	8(1)	24(1)	-1(1)	-6(1)	-1(1)

C(21)	22(1)	15(1)	19(1)	-6(1)	-7(1)	-4(1)
C(22)	18(1)	13(1)	22(1)	-3(1)	-9(1)	-6(1)
C(23)	15(1)	14(1)	19(1)	-2(1)	-5(1)	-3(1)
C(24)	18(1)	17(1)	19(1)	1(1)	-5(1)	-4(1)
C(25)	27(1)	22(1)	16(1)	2(1)	1(1)	-1(1)

Table S10. Hydrogen coordinates (x 10^4) and isotropic displacement parameters ($Å^2$x 10^3) for **2Ba(OTf)$_2$**.

	x	y	z	U(eq)
H(13)	5050(40)	7330(30)	8190(20)	36(9)
H(3)	11742	5791	8766	21
H(4)	12752	4038	9476	24
H(5)	11744	2417	9643	22
H(7)	10017	1444	9400	20
H(8A)	8253	419	9561	24
H(8B)	8398	561	8401	24
H(9A)	6050	455	9065	25
H(9B)	5948	1276	9869	25
H(10)	4551	1547	8249	20
H(12)	3035	2360	7242	23
H(13A)	2123	3882	6265	25
H(14)	3199	5537	5797	23
H(17A)	3835	7261	6252	21
H(17B)	4463	6750	5230	21
H(18A)	6510	7557	4956	20
H(18B)	5197	8547	5172	20
H(19A)	6222	9715	5885	21
H(19B)	7666	8909	5492	21
H(20A)	7718	9834	6847	21

H(20B)	6503	9141	7522	21
H(21A)	8018	8130	8535	21
H(21B)	9156	8917	7900	21
H(22A)	10750	7252	7453	19
H(22B)	10314	7251	8619	19
H(25A)	4366	5832	9027	35
H(25B)	4616	6635	9729	35
H(25C)	5807	5614	9353	35

Comparison of ωB97X-D and PBE0 spectra for Ni(II) complexes

Ni(II)3'-OCH_3-salen			Ni(II)Na			Ni(II)Ba		
**ωB97X-D**	**PBE0**	**Exp.**	**ωB97X-D**	**PBE0**	**Exp.**	**ωB97X-D**	**PBE0**	**Exp.**
608	618		595	608		640	652	
563	557	547	565	563	552	607	603	555
504	498		507	505		539	537	
464	463		452	454		471	474	
390	419	415	389	418	406	386	415	404
	403			388			379	
	385			369			360	
356	347	349	344	336	345	337	331	344

wavelengths (nm)

UωB97X-D/def2-TZVP//UωB97X-D/def2-SVP SMD(*N,N*-Dimethylformamide or Acetonitrile)-Solvated Electronic Energies (Hartrees), Free Energy Corrections (Hartrees), and Cartesian Coordinates (Å)

Ni(II)(3'-OCH_3-salen) singlet
Electronic Energy: -2615.667902
Free Energy Correction: 0.291209

Ni	0.000010000	-0.882227000	-0.000162000
O	-1.281050000	0.462781000	0.079427000
O	1.281047000	0.462775000	-0.079861000
O	2.639463000	2.695930000	0.027431000
O	-2.639523000	2.695896000	-0.027636000
N	-1.262769000	-2.244816000	0.177521000
N	1.262822000	-2.244833000	-0.177611000
C	-2.557632000	0.348157000	0.036027000
C	-3.355385000	1.554282000	-0.025188000
C	-4.733954000	1.486592000	-0.078978000
H	-5.324668000	2.402481000	-0.130170000
C	-5.408577000	0.240298000	-0.067057000
H	-6.499504000	0.220925000	-0.108823000
C	-4.684756000	-0.923115000	0.001420000
H	-5.185898000	-1.894792000	0.019390000
C	-3.264052000	-0.887342000	0.053246000
C	-2.547592000	-2.125071000	0.161462000
H	-3.150116000	-3.040511000	0.252347000

C	-0.647235000	-3.551194000	0.392964000
H	-1.314791000	-4.370606000	0.088015000
H	-0.430533000	-3.660222000	1.468557000
C	0.647371000	-3.551362000	-0.392351000
H	1.314978000	-4.370568000	-0.086978000
H	0.430652000	-3.660973000	-1.467883000
C	2.547641000	-2.125066000	-0.161210000
H	3.150208000	-3.040536000	-0.251492000
C	3.264070000	-0.887291000	-0.053330000
C	4.684771000	-0.923029000	-0.001414000
H	5.185935000	-1.894697000	-0.019226000
C	5.408566000	0.240407000	0.066987000
H	6.499494000	0.221060000	0.108759000
C	4.733912000	1.486682000	0.078807000
H	5.324595000	2.402593000	0.130001000
C	3.355345000	1.554336000	0.024960000
C	2.557627000	0.348198000	-0.036305000
C	3.327315000	3.917498000	0.086374000
H	3.996216000	4.057074000	-0.781305000
H	3.923758000	4.010199000	1.011337000
C	-3.327451000	3.917474000	-0.085616000
H	-3.996050000	4.056454000	0.782383000
H	-3.924165000	4.010762000	-1.010339000
H	-2.567818000	4.709725000	-0.075743000
H	2.567658000	4.709723000	0.076669000

2Na singlet
Electronic Energy: -3084.442312
Free Energy Correction: 0.39086

Ni	-0.084411000	-1.887197000	0.132576000
Na	0.076750000	1.475429000	0.047735000
O	-1.275286000	-0.468198000	-0.011796000
O	1.230114000	-0.610355000	-0.162315000
O	2.651227000	1.498889000	-0.637227000
O	1.409084000	3.612908000	0.436878000
O	-1.278142000	3.424007000	1.052858000
O	-2.471482000	1.739750000	-0.685926000
N	-1.420174000	-3.137629000	0.480037000
N	1.107945000	-3.319312000	0.196166000
C	-2.552154000	-0.557039000	-0.227639000

C	-3.252020000	0.634009000	-0.594803000
C	-4.614956000	0.620784000	-0.823612000
H	-5.128385000	1.541318000	-1.108398000
C	-5.352593000	-0.574822000	-0.696363000
H	-6.428505000	-0.566118000	-0.879740000
C	-4.708394000	-1.737955000	-0.338483000
H	-5.265139000	-2.671838000	-0.226090000
C	-3.310969000	-1.748206000	-0.105713000
C	-2.685939000	-2.977379000	0.314131000
H	-3.348799000	-3.830590000	0.514790000
C	-0.875524000	-4.392392000	0.988879000
H	-1.576274000	-5.227636000	0.846085000
H	-0.689527000	-4.271527000	2.068464000
C	0.434058000	-4.616056000	0.260624000
H	1.058454000	-5.372057000	0.757960000
H	0.235598000	-4.949154000	-0.771084000
C	2.392443000	-3.255313000	0.132032000
H	2.962235000	-4.192900000	0.198482000
C	3.155965000	-2.048310000	-0.054018000
C	4.570066000	-2.141558000	-0.105228000
H	5.035476000	-3.121730000	0.025606000
C	5.339474000	-1.021435000	-0.317939000
H	6.428192000	-1.090333000	-0.353868000
C	4.717078000	0.232674000	-0.499626000
H	5.336607000	1.112519000	-0.678667000
C	3.340269000	0.345132000	-0.461382000
C	2.512698000	-0.796888000	-0.217119000
C	3.358412000	2.720088000	-0.563415000
H	4.051824000	2.833698000	-1.413758000
H	3.943182000	2.753020000	0.372238000
C	2.351569000	3.838847000	-0.584952000
H	2.891093000	4.789068000	-0.428703000
H	1.844823000	3.896338000	-1.566795000
C	0.735026000	4.770071000	0.872143000
H	0.392456000	5.367779000	0.007804000
H	1.401782000	5.414998000	1.473600000
C	-0.435170000	4.332940000	1.723278000
H	-0.071308000	3.807939000	2.618674000
H	-0.998652000	5.224353000	2.054727000
C	-1.959854000	3.952553000	-0.063057000

H	-1.277362000	4.087227000	-0.923352000
H	-2.401558000	4.937842000	0.170640000
C	-3.064887000	2.999305000	-0.430382000
H	-3.781312000	2.911309000	0.403629000
H	-3.601752000	3.376562000	-1.316780000

2Ba singlet
Electronic Energy: -2947.678159
Free Energy Correction: 0.391444

Ba	1.422952000	-0.338194000	-0.829792000
Ni	-2.191401000	0.412455000	0.035931000
O	-1.100070000	-1.113691000	-0.018494000
O	-0.609243000	1.436981000	0.026948000
O	1.832097000	2.474029000	-0.188014000
O	3.949565000	0.732984000	0.252089000
O	3.430087000	-2.049286000	0.458270000
O	0.777178000	-2.897033000	0.389178000
N	-3.763822000	-0.563738000	-0.204017000
N	-3.298177000	1.883851000	0.318783000
C	-1.481057000	-2.362722000	0.025479000
C	-0.485567000	-3.373727000	0.204000000
C	-0.817495000	-4.715890000	0.182399000
H	-0.043605000	-5.476786000	0.288805000
C	-2.157682000	-5.121084000	0.027850000
H	-2.399480000	-6.185152000	0.018777000
C	-3.144730000	-4.171465000	-0.103940000
H	-4.191294000	-4.465869000	-0.213237000
C	-2.822044000	-2.793026000	-0.104232000
C	-3.894340000	-1.841820000	-0.248442000
H	-4.900890000	-2.253259000	-0.406295000
C	-4.921137000	0.304327000	-0.410697000
H	-5.862123000	-0.215400000	-0.181331000
H	-4.937012000	0.612220000	-1.468699000
C	-4.703449000	1.509897000	0.475580000
H	-5.372969000	2.341881000	0.215453000
H	-4.872655000	1.236901000	1.529793000
C	-2.939112000	3.110364000	0.457196000
H	-3.721820000	3.857200000	0.649339000
C	-1.587775000	3.602839000	0.389093000
C	-1.389828000	4.993003000	0.567672000

H	-2.261653000	5.626920000	0.745888000
C	-0.125144000	5.530639000	0.524929000
H	0.036639000	6.599661000	0.671942000
C	0.976244000	4.688260000	0.282826000
H	1.972996000	5.127475000	0.245342000
C	0.805156000	3.329041000	0.088086000
C	-0.494112000	2.729663000	0.164238000
C	3.162771000	2.961633000	-0.076966000
H	3.360554000	3.252709000	0.968192000
H	3.312421000	3.840614000	-0.723653000
C	4.126309000	1.895795000	-0.525624000
H	3.980528000	1.669174000	-1.600703000
H	5.149366000	2.294675000	-0.410810000
C	4.945196000	-0.249529000	0.046071000
H	5.937974000	0.142346000	0.328474000
H	4.987658000	-0.539191000	-1.021479000
C	4.630459000	-1.445125000	0.901997000
H	5.465242000	-2.165543000	0.842282000
H	4.528219000	-1.127213000	1.955280000
C	2.951416000	-3.031572000	1.350783000
H	2.615325000	-2.555551000	2.290644000
H	3.751530000	-3.750266000	1.601724000
C	1.818821000	-3.801518000	0.728017000
H	2.151011000	-4.342119000	-0.174278000
H	1.467646000	-4.535820000	1.469775000

Fe(II)(3’-OCH_3-salen)-MeCN quintet
Electronic Energy: -2503.825399
Free Energy Correction: 0.322683

C	-2.765913213	0.478247037	-0.530917041
C	-3.696699285	1.574264120	-0.362723028
C	-5.063143386	1.363864103	-0.371917028
H	-5.748929456	2.201690168	-0.235555018
C	-5.595588442	0.066289005	-0.557117040
H	-6.678243500	-0.075993006	-0.564382041
C	-4.742303363	-0.997827077	-0.727871056
H	-5.138259392	-2.006641155	-0.877126065
C	-3.333191255	-0.820145063	-0.711627055
C	-2.513167194	-1.996157153	-0.924504071
H	-3.065840236	-2.921758225	-1.165296087

C	-0.519037040	-3.264072250	-1.159142089
H	-0.151617012	-3.208279246	-2.198580167
H	-1.166591090	-4.153422315	-1.076993084
C	0.679714053	-3.379451258	-0.222577017
H	0.315321024	-3.567595271	0.802429061
H	1.322459100	-4.226842324	-0.516377039
C	2.676045205	-2.092716158	-0.275877021
H	3.227286245	-3.048303233	-0.328562025
C	3.500873269	-0.901011067	-0.268241021
C	4.907170376	-1.085133084	-0.351251027
H	5.295579391	-2.105810162	-0.414576032
C	5.766497453	-0.012348001	-0.353292027
H	6.846676544	-0.159086012	-0.417476032
C	5.243895401	1.299447097	-0.270621021
H	5.933952471	2.144828163	-0.273283021
C	3.880741295	1.514778115	-0.187559014
C	2.944184222	0.411123031	-0.183096014
C	4.121924314	3.868404295	-0.116861009
H	4.710541361	3.943704302	-1.048540080
H	4.816687369	3.885777295	0.741709056
C	-3.921804301	3.897422295	0.029374002
H	-4.533107346	3.785483289	0.942533071
H	-4.594639351	4.097330315	-0.823403064
Fe	0.057479004	-0.448107034	-0.187499014
N	1.393060108	-2.116797162	-0.226766017
N	-1.231223095	-2.035215155	-0.861264067
O	-1.507415116	0.729166058	-0.515499039
O	1.689253129	0.670817054	-0.101732008
O	3.300301254	2.731462207	-0.106278008
O	-3.106953238	2.777701213	-0.194192015
H	-3.252081247	4.757646365	0.157653012
H	3.458561262	4.740397361	-0.048953004
C	-0.683594054	-0.798287063	3.028790229
N	-0.381947029	-0.673299054	1.923027149
C	-1.064502082	-0.952765073	4.418504339
H	-2.153502166	-0.841315063	4.511305343
H	-0.565966043	-0.182165014	5.022292384
H	-0.766073060	-1.948415147	4.773935367

Fe(II)K-MeCN quintet

Electronic Energy: -3410.238287
Free Energy Correction: 0.42084

C	-2.431942185	1.392376104	-0.383784029
C	-2.228690173	2.812561214	-0.466574036
C	-3.299603253	3.682261281	-0.547579044
H	-3.139752242	4.761374366	-0.569434043
C	-4.617828353	3.179873244	-0.614640047
H	-5.453696442	3.878631296	-0.685352050
C	-4.838981368	1.819914141	-0.600448047
H	-5.857292468	1.427523110	-0.671111049
C	-3.764745287	0.901374067	-0.485336037
C	-4.086023311	-0.521864040	-0.493043038
H	-5.153373393	-0.767882057	-0.629083045
C	-3.609428278	-2.873883221	-0.356960027
H	-4.605649349	-3.045229232	-0.797825060
H	-3.640988276	-3.210074247	0.693774054
C	-2.549833192	-3.682887282	-1.110965083
H	-2.710996208	-4.761752362	-0.953432075
H	-2.652529203	-3.483662266	-2.191293169
C	-0.186064014	-3.995054307	-0.975482076
H	-0.341986026	-4.986644380	-1.434098111
C	1.206879095	-3.616547278	-0.787049058
C	2.201335170	-4.559218348	-1.160129089
H	1.881653145	-5.545320413	-1.508518117
C	3.540947270	-4.246207322	-1.103485083
H	4.295360327	-4.977887380	-1.399283106
C	3.951199304	-2.963542225	-0.672061049
H	5.015532383	-2.724107210	-0.646224049
C	3.009959229	-2.027493157	-0.292964022
C	1.603361121	-2.324386179	-0.339102026
C	4.610037351	-0.300676023	0.148087011
H	4.987130381	-0.218469017	-0.886803066
H	5.270747405	-0.988404076	0.703488055
C	4.650494354	1.038823079	0.840641066
H	4.303463327	0.930353073	1.886419143
H	5.703351420	1.374914107	0.873022068
C	3.954233304	3.283006249	0.673916053
H	5.001299383	3.634858277	0.625677049
H	3.643883278	3.304056254	1.736561131
C	3.098783239	4.226190325	-0.131911010

H	3.331558257	5.263061402	0.173300013
H	3.353348257	4.127889317	-1.204153091
C	0.889448068	4.692969358	-0.778805059
H	1.032343081	4.368857332	-1.827700138
H	1.136255088	5.769598432	-0.728051055
C	-0.560885042	4.538128349	-0.393165030
H	-0.747560058	4.927135376	0.623397047
H	-1.149414086	5.139050391	-1.106013085
Fe	-1.182315089	-1.294033100	0.017989001
K	1.190406093	1.258690098	0.069633005
N	-3.233790246	-1.471728111	-0.361165028
N	-1.211843095	-3.269097249	-0.699158051
O	0.787639059	-1.388398104	0.009796001
O	-1.384920108	0.650064050	-0.228470018
O	-0.917203071	3.172867244	-0.457028035
O	1.729847131	3.951704303	0.075160006
O	3.852991293	1.975367150	0.154310012
O	3.278915249	-0.769604058	0.144110011
C	-1.608614121	-1.562156121	3.280668249
N	-1.439738108	-1.492683112	2.142145165
C	-1.822748140	-1.651123125	4.710657362
H	-1.218302095	-2.473254190	5.117954390
H	-2.885784221	-1.844442142	4.909663372
H	-1.524426116	-0.706508054	5.185663397

Fe(II)Ba-MeCN quintet
Electronic Energy: -2835.836772
Free Energy Correction: 0.422766

Ba	1.593438122	0.046097003	-0.905026067
Fe	-1.975128152	0.025556002	0.202292016
O	-0.628048049	-1.409582107	-0.035533003
O	-0.643127050	1.481422115	0.047132004
O	1.523753114	2.846768215	0.215520016
O	3.928384297	1.394354105	0.400573030
O	3.958265302	-1.524990119	-0.086663007
O	1.496403112	-2.826413217	0.213937017
N	-3.358323259	-1.254781098	-0.748505058
N	-3.434661263	1.436609110	-0.355192027
N	-2.574247197	-0.197179015	2.222859169
C	-0.778857061	-2.678183203	-0.273621021

C	0.378198029	-3.512795268	-0.143130011
C	0.311650024	-4.873915370	-0.369211028
H	1.198772094	-5.499567431	-0.256803020
C	-0.911983071	-5.462172433	-0.755035057
H	-0.955452071	-6.537971523	-0.935405070
C	-2.034737154	-4.678771358	-0.917964070
H	-2.977394228	-5.129051391	-1.240538096
C	-1.997184154	-3.280747250	-0.683654052
C	-3.200482243	-2.508983191	-0.975648073
H	-4.011719305	-3.072984234	-1.466483113
C	-4.527209348	-0.542332040	-1.253631095
H	-5.440506434	-1.155923088	-1.192389089
H	-4.348868333	-0.312962024	-2.317828175
C	-4.718667359	0.773646057	-0.492721038
H	-5.464434401	1.402819105	-1.006251076
H	-5.093760391	0.556229044	0.521580040
C	-3.266422247	2.680738203	-0.613728047
H	-4.128994313	3.286724249	-0.940518070
C	-1.996463150	3.396312258	-0.514269039
C	-2.000448151	4.787454364	-0.782678060
H	-2.948736226	5.267091404	-1.040359080
C	-0.840457066	5.531888413	-0.726948054
H	-0.857476068	6.602402505	-0.939954073
C	0.381782029	4.911119377	-0.395272030
H	1.295559099	5.506391436	-0.354197027
C	0.413101031	3.556728272	-0.124917009
C	-0.772370057	2.754527213	-0.188172014
C	2.759765210	3.496366267	0.439564034
H	2.622289200	4.391414337	1.068243080
H	3.206278247	3.808896292	-0.520461040
C	3.666609282	2.543856193	1.178093090
H	4.608857354	3.072139233	1.409782109
H	3.196068243	2.254941174	2.136373164
C	4.815375369	0.490577037	1.032141079
H	4.395070333	0.157202012	1.999594154
H	5.778249421	0.990718073	1.242669093
C	5.083571390	-0.690609050	0.125571009
H	5.392911386	-0.324681025	-0.865182069
H	5.917370438	-1.277837096	0.546227044
C	3.742278284	-2.519536191	0.895798068

H	3.395434258	-2.069064157	1.843248138
H	4.680483358	-3.064370234	1.098817084
C	2.720169210	-3.509007269	0.395761030
H	3.052578233	-3.958604303	-0.556102041
H	2.610569199	-4.312913327	1.143241085
C	-2.911484222	-0.360786028	3.313197251
C	-3.342176256	-0.565637043	4.680394355
H	-2.471849188	-0.796749063	5.309599413
H	-4.055192312	-1.401131109	4.714635362
H	-3.831091292	0.347503026	5.046927384

Fe(II)(3’-OCH_3-salen)-Cl quintet
Electronic Energy: -2831.440081
Free Energy Correction: 0.280865

C	2.865415220	0.422177032	-0.137735011
C	3.775994289	1.546478118	-0.237088018
C	5.131309390	1.363283103	-0.439740034
H	5.798085419	2.224487172	-0.509248039
C	5.677214456	0.063530005	-0.560100044
H	6.750563509	-0.058650005	-0.720281056
C	4.845375369	-1.028089078	-0.473846036
H	5.250863400	-2.040302157	-0.565478041
C	3.449316266	-0.877006069	-0.264616020
C	2.654085201	-2.088420159	-0.192589015
H	3.216408245	-3.033469230	-0.306326023
C	0.686256054	-3.398034259	0.040781003
H	0.337253025	-3.542484270	1.077253084
H	1.325108103	-4.253987323	-0.239774018
C	-0.526645040	-3.306027254	-0.880243067
H	-0.172118013	-3.273194250	-1.925650148
H	-1.170308089	-4.195992323	-0.771672057
C	-2.517520190	-2.037191155	-0.713731053
H	-3.058010233	-2.963151226	-0.982812075
C	-3.352949257	-0.865600066	-0.538300043
C	-4.757361366	-1.042921081	-0.651961050
H	-5.141772394	-2.051177157	-0.833775066
C	-5.622053443	0.020094002	-0.539385041
H	-6.701512535	-0.122046010	-0.624519045
C	-5.103306388	1.317743099	-0.314648024
H	-5.797325467	2.155606164	-0.228119018

C	-3.741601287	1.526692117	-0.202515016
C	-2.797003212	0.431256033	-0.306732023
C	-3.989940306	3.863068294	0.084183006
H	-4.705018359	3.803229290	0.924123069
H	-4.558024350	4.021291307	-0.849969064
C	3.973845301	3.904377298	-0.160614012
H	4.486908341	4.013897309	-1.132904084
H	4.733902362	3.915443300	0.640941050
Cl	-0.362059028	-0.866641069	2.631803203
Fe	-0.000882000	-0.512608039	0.280022021
N	-1.239505094	-2.072895157	-0.599339045
N	1.386300107	-2.134137163	-0.012998001
O	1.621279121	0.654315048	0.054946004
O	-1.547058117	0.678713050	-0.200768015
O	-3.166153239	2.731252209	0.010948001
O	3.177122242	2.751976208	-0.112612008
H	3.302578251	4.761608362	-0.019859002
H	-3.331321254	4.726326358	0.246370019

Fe(II)K-Cl quintet
Electronic Energy: -3737.857351
Free Energy Correction: 0.381412

C	-2.745858210	-0.634260050	-0.376340029
C	-3.513426269	0.587277043	-0.391217030
C	-4.879612370	0.584948044	-0.188024014
H	-5.442280395	1.520060118	-0.185231014
C	-5.567170406	-0.632738046	0.021352002
H	-6.648787483	-0.620367046	0.170218013
C	-4.866349373	-1.818811140	0.044105003
H	-5.390378426	-2.764042211	0.215073017
C	-3.458791264	-1.845461141	-0.124380009
C	-2.788128211	-3.135249240	-0.024354002
H	-3.450474264	-4.019291308	-0.020477002
C	-0.911701072	-4.609979353	0.187920014
H	-1.590871120	-5.422636436	-0.121499009
H	-0.641015050	-4.775962364	1.245190093
C	0.362407028	-4.626902351	-0.660384049
H	0.951482072	-5.537104407	-0.456716035
H	0.074911006	-4.645737357	-1.725988133
C	2.395665185	-3.381826259	-0.617245049

H	2.920945223	-4.317881327	-0.879234069
C	3.239126247	-2.200414169	-0.521148040
C	4.646800353	-2.365890180	-0.589958045
H	5.049848384	-3.374883257	-0.717658056
C	5.497161421	-1.285957099	-0.495821038
H	6.578747488	-1.422313111	-0.557547045
C	4.971895379	0.014873001	-0.302917023
H	5.659366419	0.857510067	-0.206506016
C	3.606995274	0.205054015	-0.224807017
C	2.688077203	-0.892173066	-0.396924031
C	3.721050286	2.574416195	0.151246012
H	4.089660314	2.901212220	-0.837434062
H	4.591378349	2.436769187	0.815537062
C	2.838850216	3.628487279	0.775526060
H	2.552944194	3.315533251	1.799100140
H	3.431021264	4.558157348	0.867422065
C	0.915690069	4.938017377	0.434185033
H	1.493743113	5.877395436	0.351960027
H	0.633971050	4.812468366	1.497753112
C	-0.327164025	5.065436385	-0.408286031
H	-0.813805064	6.030931452	-0.175508013
H	-0.047819004	5.080987385	-1.478886112
C	-2.353916180	4.011188308	-0.963297071
H	-2.072703156	3.826206293	-2.017819156
H	-2.851247219	4.997925380	-0.919121069
C	-3.349546256	2.971678230	-0.512322039
H	-3.673554278	3.164051241	0.526516040
H	-4.234227321	3.051104231	-1.167564088
Cl	-0.205746016	-0.774197058	2.458688190
Fe	-0.076228006	-1.762270133	0.284643022
K	0.058230005	1.556610121	0.119483009
N	-1.521131115	-3.299698251	0.083227006
N	1.125212085	-3.410187259	-0.423222032
O	1.434576111	-0.627176049	-0.430717033
O	-1.481563114	-0.555691040	-0.583387045
O	-2.756880210	1.696691128	-0.609332044
O	-1.209470095	3.998346303	-0.143182011
O	1.689252129	3.845558295	-0.006772001
O	2.979271230	1.383973108	0.021932002

Fe(II)Ba-Cl quintet
Electronic Energy: -3163.459114
Free Energy Correction: 0.383544

C	-2.605134199	-1.236943093	-0.261968020
C	-2.384776183	-2.652633202	-0.201437015
C	-3.436034261	-3.548552272	-0.167113013
H	-3.248177247	-4.623886353	-0.166481013
C	-4.764736366	-3.077634236	-0.123567009
H	-5.588901441	-3.792699287	-0.092068007
C	-5.008802380	-1.719995131	-0.105518008
H	-6.034906473	-1.346925102	-0.042787003
C	-3.949898300	-0.781821057	-0.154947012
C	-4.295536326	0.632700046	-0.061139005
H	-5.373867408	0.864165064	-0.121643009
C	-3.876118297	2.967930226	0.235902018
H	-3.811573293	3.250524249	1.300936101
H	-4.915948374	3.127435240	-0.095737007
C	-2.916022224	3.842225293	-0.572961041
H	-3.117974238	3.686066283	-1.646499127
H	-3.080420233	4.909907376	-0.351554027
C	-0.570204044	4.249350323	-0.439836033
H	-0.777761061	5.310785405	-0.663181052
C	0.837585062	3.895027297	-0.298777023
C	1.784756134	4.939120379	-0.175205013
H	1.431361111	5.973597478	-0.207497016
C	3.127512240	4.668439358	-0.007602001
H	3.851779292	5.479977395	0.085326007
C	3.572528272	3.330726252	0.054348004
H	4.633769352	3.125207239	0.206576016
C	2.666860201	2.295154174	-0.074266006
C	1.273244097	2.541563194	-0.302428023
C	4.255268324	0.569009045	0.454770034
H	4.569039349	1.170660087	1.324071101
H	4.999351381	0.699282054	-0.349417027
C	4.178962319	-0.873132068	0.890949069
H	5.174394393	-1.179570088	1.257943096
H	3.462964265	-0.965617074	1.727732131
C	3.965888301	-3.074925235	-0.022280002
H	4.966216381	-3.281153251	0.396253030
H	3.918479301	-3.520761269	-1.027175078

C	2.924110222	-3.719834284	0.864430065
H	3.199833244	-4.779491363	1.012874079
H	2.900635221	-3.241084246	1.860744140
C	0.615787049	-4.161618318	1.049521080
H	0.448745035	-3.515903269	1.931977150
H	0.889995068	-5.168721396	1.410329109
C	-0.663223053	-4.283236326	0.259899020
H	-0.534365038	-4.954913377	-0.605307048
H	-1.420681107	-4.719394359	0.928939069
Ba	1.029153079	-1.074420081	-1.040502079
Cl	-0.730125054	1.024917079	2.613030201
Fe	-1.356064104	1.407329108	0.398069030
N	-1.546261118	3.427109263	-0.313616024
N	-3.457401263	1.584607122	0.117721009
O	-1.576280121	-0.466090035	-0.407285031
O	0.493583038	1.527622114	-0.497434038
O	2.975779226	0.969604075	0.005366000
O	3.783289288	-1.682961127	-0.198524015
O	1.652858128	-3.630000276	0.251816019
O	-1.063131079	-2.998380228	-0.185303014

Fe(III)(3’-OCH_3-salen)-MeCN quartet
Electronic Energy: -2503.661203
Free Energy Correction: 0.327534

C	-2.523544190	0.508682039	-0.494991038
C	-3.289540250	1.702742127	-0.286271022
C	-4.672482356	1.635462124	-0.225671017
H	-5.256121401	2.541531195	-0.057634004
C	-5.349848423	0.406585031	-0.378713029
H	-6.440181473	0.388758030	-0.329602025
C	-4.639334355	-0.750789055	-0.590983046
H	-5.147990391	-1.708848129	-0.720631054
C	-3.222086248	-0.714236052	-0.642119047
C	-2.507515189	-1.934116148	-0.897003068
H	-3.108182237	-2.824051218	-1.131658088
C	-0.580932043	-3.330425256	-1.222067095
H	-0.278817021	-3.284338252	-2.280405173
H	-1.272295097	-4.174579319	-1.090368081
C	0.644500048	-3.458422263	-0.339141026
H	0.342476026	-3.684340281	0.696356053

H	1.318147099	-4.251967327	-0.691178053
C	2.599359198	-2.066508157	-0.338254026
H	3.177004241	-3.000940229	-0.363118028
C	3.352319256	-0.846449067	-0.281531022
C	4.769413362	-0.923591071	-0.280995022
H	5.244131398	-1.905512144	-0.341685026
C	5.520980429	0.224062017	-0.203212016
H	6.611516524	0.176524014	-0.201543015
C	4.884719375	1.481912112	-0.121906009
H	5.499832443	2.380411180	-0.058018005
C	3.503339267	1.587168123	-0.121514009
C	2.694663206	0.405129031	-0.206551016
C	3.529153269	3.949493300	0.016723001
H	4.172425319	4.092633311	-0.868206068
H	4.151374320	4.007304305	0.926434071
C	-3.232608244	4.042741311	0.063487005
H	-3.805931290	4.027842309	1.006309079
H	-3.914926299	4.290251327	-0.767520058
Fe	0.039960003	-0.693012055	-0.307078023
N	1.311196102	-2.156732164	-0.344477026
N	-1.223591094	-2.063533160	-0.874094064
O	-1.226612093	0.617308047	-0.562125042
O	1.401875106	0.550948040	-0.211150016
O	2.814844217	2.738562210	-0.049300004
O	-2.562662195	2.825000218	-0.158223012
H	-2.459219187	4.818071369	0.129808010
H	2.781938213	4.752266363	0.046388004
C	-0.643892050	-1.079552084	2.905677222
N	-0.373941029	-0.946632073	1.793523135
C	-0.981786074	-1.247008098	4.302365327
H	-1.959776149	-0.785791061	4.496832346
H	-0.214864017	-0.761620060	4.921412377
H	-1.023988076	-2.318831178	4.539806349

Fe(III)K-MeCN quartet

Electronic Energy: -3410.070222

Free Energy Correction: 0.428448

C	-0.139968010	2.739271207	-0.225170017
C	1.174882091	3.297898250	-0.240769018
C	1.355912103	4.661466357	-0.073592006

H	2.361455183	5.082476390	-0.055650004
C	0.253427020	5.525967396	0.069144005
H	0.425958032	6.596045498	0.194637015
C	-1.025092078	5.019119384	0.040971003
H	-1.892296143	5.676283421	0.134467010
C	-1.236293094	3.625812277	-0.103877008
C	-2.591629199	3.148288242	-0.142980011
H	-3.382543257	3.909519298	-0.090070007
C	-4.366296336	1.527189115	-0.234002018
H	-5.006281380	2.363228181	-0.548018044
H	-4.639209353	1.239972093	0.794391059
C	-4.503141345	0.336337025	-1.162639088
H	-5.465720407	-0.176858013	-1.029194080
H	-4.419214336	0.663430052	-2.211100168
C	-3.485980264	-1.838873139	-1.033923078
H	-4.461524343	-2.237321170	-1.345007104
C	-2.434107187	-2.799751214	-0.826074065
C	-2.750893210	-4.172954321	-0.960600072
H	-3.773606286	-4.456103340	-1.219147095
C	-1.779600138	-5.129092391	-0.768289058
H	-2.013748153	-6.189364474	-0.876529068
C	-0.471508036	-4.744026364	-0.420133032
H	0.280620021	-5.517542418	-0.263565020
C	-0.139141011	-3.406222261	-0.270367020
C	-1.116439083	-2.393443180	-0.508607039
C	2.132701161	-3.888636297	0.297755023
H	2.413495183	-4.337511332	-0.670038052
H	1.813989140	-4.693358357	0.979553073
C	3.310007254	-3.189719243	0.927869072
H	3.029841233	-2.796300216	1.924765147
H	4.103480312	-3.943661301	1.081250082
C	4.978174379	-1.569919119	0.518020039
H	5.805592433	-2.295661173	0.416980032
H	4.918476375	-1.272961098	1.582261120
C	5.276631401	-0.361373028	-0.330232025
H	6.297393470	-0.004089000	-0.102109008
H	5.247819398	-0.640662047	-1.400288108
C	4.420313337	1.758333134	-0.916699072
H	4.149931317	1.470548115	-1.951200149
H	5.452505423	2.152967163	-0.940678072

C	3.519264269	2.867262218	-0.437980034
H	3.808586289	3.199563242	0.573514042
H	3.630809277	3.715704283	-1.132352089
Fe	-1.798567136	0.375699028	-0.270296021
K	1.772809138	-0.291077022	-0.059218004
N	-2.953740227	1.913108145	-0.236081018
N	-3.382090260	-0.563427045	-0.874548067
O	-0.735706054	-1.139325089	-0.436191034
O	-0.257004020	1.438596112	-0.332198025
O	2.176775165	2.405135182	-0.424763032
O	4.328091331	0.647735051	-0.057835004
O	3.761005285	-2.147573164	0.097366008
O	1.081983082	-2.953425223	0.101781008
C	-2.086461161	-0.102633008	2.972110229
N	-1.988595152	0.088590007	1.840026142
C	-2.206141168	-0.344615026	4.392219338
H	-1.310042099	-0.873665066	4.744777361
H	-3.097099238	-0.959441073	4.580208349
H	-2.301194176	0.615906045	4.916988376

Fe(III)Ba-MeCN quartet
Electronic Energy: -2835.666118
Free Energy Correction: 0.4328

Ba	1.734738000	-0.032760000	-1.206243000
Fe	-2.053854000	0.212541000	-0.166508000
O	-0.815352000	-1.162297000	-0.327987000
O	-0.610666000	1.382940000	0.001881000
O	1.675080000	2.526790000	0.418932000
O	3.998093000	0.951148000	0.420669000
O	3.807410000	-1.814996000	-0.196375000
O	1.190618000	-2.690649000	0.293184000
N	-3.495744000	-0.911091000	-0.819151000
N	-3.331122000	1.634308000	-0.364123000
N	-2.454558000	-0.121405000	1.898006000
C	-1.067556000	-2.451927000	-0.266112000
C	0.015895000	-3.318876000	0.047496000
C	-0.176681000	-4.691098000	0.088965000
H	0.652979000	-5.357659000	0.325899000
C	-1.443843000	-5.244272000	-0.172487000
H	-1.571627000	-6.327234000	-0.135705000

C	-2.509381000	-4.423892000	-0.471243000
H	-3.497765000	-4.840505000	-0.676705000
C	-2.337264000	-3.020262000	-0.513105000
C	-3.473195000	-2.199529000	-0.851435000
H	-4.388225000	-2.724420000	-1.159752000
C	-4.666318000	-0.148015000	-1.258919000
H	-5.586979000	-0.736050000	-1.140230000
H	-4.535299000	0.092005000	-2.325795000
C	-4.702959000	1.128357000	-0.439243000
H	-5.378746000	1.875015000	-0.878608000
H	-5.036452000	0.910744000	0.588165000
C	-3.067894000	2.896930000	-0.397666000
H	-3.908709000	3.591125000	-0.531194000
C	-1.763318000	3.487453000	-0.247872000
C	-1.659157000	4.898781000	-0.300584000
H	-2.562616000	5.487034000	-0.475353000
C	-0.437728000	5.512166000	-0.134011000
H	-0.350994000	6.598837000	-0.179891000
C	0.713678000	4.737921000	0.103689000
H	1.672955000	5.239516000	0.236532000
C	0.632207000	3.357073000	0.172334000
C	-0.613310000	2.695910000	-0.026520000
C	2.937680000	3.076128000	0.764606000
H	2.819476000	3.864780000	1.524718000
H	3.412799000	3.514252000	-0.129464000
C	3.795525000	1.989445000	1.354793000
H	4.760178000	2.444354000	1.640567000
H	3.324305000	1.587946000	2.270671000
C	5.019795000	0.054261000	0.808252000
H	4.855802000	-0.279284000	1.848797000
H	6.005313000	0.552594000	0.773381000
C	5.039786000	-1.122190000	-0.140224000
H	5.237799000	-0.775576000	-1.165589000
H	5.855339000	-1.805867000	0.153155000
C	3.458719000	-2.537034000	0.967316000
H	3.156164000	-1.855777000	1.782794000
H	4.316176000	-3.135960000	1.319615000
C	2.323421000	-3.470592000	0.647178000
H	2.596620000	-4.139957000	-0.185698000
H	2.103836000	-4.081432000	1.537963000

C	-2.673518000	-0.361885000	3.003406000
C	-2.949913000	-0.665158000	4.389297000
H	-2.078644000	-0.391257000	5.000078000
H	-3.150035000	-1.740793000	4.490461000
H	-3.828332000	-0.092295000	4.716946000

Fe(III)(3'-OCH_3-salen)-Cl sextet
Electronic Energy: -2831.296981
Free Energy Correction: 0.284556

C	2.743439208	0.409284031	-0.119035009
C	3.558476272	1.587448121	-0.202548016
C	4.919534375	1.481277115	-0.445141034
H	5.535671448	2.379166179	-0.509739039
C	5.528686414	0.220867017	-0.613767047
H	6.602120506	0.165850013	-0.805115064
C	4.768993364	-0.924509072	-0.538342043
H	5.227349399	-1.908280143	-0.668004053
C	3.375984258	-0.848012066	-0.290021022
C	2.629619199	-2.086348160	-0.229247018
H	3.210761246	-3.008299232	-0.391721030
C	0.706794053	-3.478442266	0.018955001
H	0.406190031	-3.683701284	1.059518079
H	1.367855105	-4.289756329	-0.322855025
C	-0.531518038	-3.375821256	-0.861066065
H	-0.220964017	-3.341048257	-1.918529148
H	-1.189065089	-4.248126325	-0.724346058
C	-2.489815189	-2.033048158	-0.716999056
H	-3.047954235	-2.927158225	-1.038575080
C	-3.275704249	-0.832163065	-0.540660041
C	-4.684735360	-0.932895071	-0.658286052
H	-5.129195391	-1.915123146	-0.837763062
C	-5.475609407	0.187578015	-0.545689040
H	-6.561655486	0.113553009	-0.629088046
C	-4.883256375	1.448822113	-0.324734025
H	-5.525384401	2.326668177	-0.240171018
C	-3.507715267	1.579742120	-0.215457016
C	-2.660154202	0.426039033	-0.319086024
C	-3.606074273	3.923041301	0.102019008
H	-4.310565330	3.887098296	0.951062075
H	-4.171447320	4.135719314	-0.821778062

C	3.618066276	3.949635299	-0.077055006
H	4.092440315	4.105621316	-1.061493080
H	4.395857336	3.988865304	0.705024055
Cl	-0.413274032	-0.955656074	2.523538195
Fe	-0.007729001	-0.616855045	0.330252025
N	-1.218134091	-2.128333163	-0.549273041
N	1.368698105	-2.185823164	-0.007283001
O	1.471009113	0.553146041	0.105125008
O	-1.374530104	0.587192046	-0.224590017
O	-2.856359220	2.738661209	-0.005798000
O	2.893895222	2.745254212	-0.031307002
H	2.897139222	4.757779363	0.099694008
H	-2.888686219	4.735659362	0.272597021

Fe(III)K-Cl sextet
Electronic Energy: -3737.707693
Free Energy Correction: 0.383616

C	-2.586367198	1.042863077	-0.247592019
C	-2.497909188	2.465467188	-0.326231025
C	-3.641937276	3.243712246	-0.246132019
H	-3.573563274	4.331582330	-0.278429021
C	-4.908683373	2.641494201	-0.126354010
H	-5.797492423	3.272683250	-0.073162005
C	-5.020759384	1.268405099	-0.079257006
H	-6.000338445	0.792031061	0.006965001
C	-3.867107297	0.450965035	-0.125236010
C	-4.050954311	-0.987991075	-0.062383005
H	-5.092299391	-1.343005105	-0.102611008
C	-3.356984255	-3.283389252	0.087407007
H	-4.372489334	-3.536005268	-0.252853019
H	-3.250026246	-3.610388274	1.135071086
C	-2.306527177	-3.970451301	-0.777536060
H	-2.306133178	-5.057426388	-0.606714046
H	-2.536723191	-3.788634291	-1.840149139
C	0.078789006	-4.047517310	-0.734088057
H	-0.012199001	-5.091390391	-1.072781082
C	1.431615111	-3.543446272	-0.606996044
C	2.506712189	-4.446787339	-0.786551060
H	2.284484172	-5.495387407	-0.998635078
C	3.810187294	-4.008165309	-0.697479051

H	4.639535352	-4.702826359	-0.841972063
C	4.086271310	-2.655741203	-0.412654032
H	5.124862392	-2.331010176	-0.338461026
C	3.052509234	-1.753315131	-0.223915017
C	1.695485128	-2.176017166	-0.357058028
C	4.501251346	0.102758008	0.210431016
H	4.967289378	0.163533013	-0.787932059
H	5.137627391	-0.523936040	0.856885067
C	4.405807334	1.466931114	0.845311066
H	4.004756306	1.377096106	1.873955146
H	5.430415395	1.874178141	0.924419070
C	3.595840276	3.655663277	0.502419038
H	4.608724351	4.087419312	0.403749031
H	3.304503255	3.723514283	1.568346119
C	2.641674200	4.459350340	-0.341890026
H	2.773911213	5.531990438	-0.109597008
H	2.882808218	4.314035331	-1.411556107
C	0.376548029	4.634161356	-0.959122074
H	0.527453040	4.247626322	-1.985283154
H	0.497179038	5.732392456	-0.995380077
C	-1.031863081	4.347166330	-0.505531039
H	-1.220113093	4.773562365	0.494889038
H	-1.716160131	4.827328367	-1.223893096
Cl	-0.908766070	-1.344238104	2.504771194
Fe	-1.079140083	-1.423891107	0.261138020
K	1.013283080	1.361867104	0.151123011
N	-3.106099236	-1.851640143	0.037629003
N	-0.998045077	-3.383004260	-0.496112038
O	0.757494059	-1.274328095	-0.254815020
O	-1.472558110	0.357003027	-0.304573023
O	-1.238080094	2.945095227	-0.475503036
O	1.313519100	4.059522310	-0.079111006
O	3.589269276	2.312194179	0.071315005
O	3.199817247	-0.445165034	0.098323008

Fe(III)Ba-Cl sextet
Electronic Energy: -3163.305896
Free Energy Correction: 0.387651

C	0.918598072	2.674816206	-0.006438000
C	-0.273258021	3.453755264	-0.006921001

C	-0.228829017	4.811913370	-0.271983021
H	-1.145482087	5.402887435	-0.286842022
C	1.003448077	5.442682411	-0.525885040
H	1.022306076	6.513967514	-0.733149054
C	2.173092165	4.711256359	-0.512862039
H	3.133274240	5.195594398	-0.706426052
C	2.150757165	3.321717253	-0.250506019
C	3.409297263	2.599030198	-0.253120019
H	4.304458328	3.189853241	-0.501866038
C	4.849716369	0.697209055	-0.032935003
H	5.143030391	0.458590035	1.002288079
H	5.619964410	1.348516105	-0.472343036
C	4.702011357	-0.587952045	-0.839254066
H	4.609433353	-0.336984026	-1.908491147
H	5.581354421	-1.237042092	-0.712623057
C	3.327463256	-2.529194195	-0.611038048
H	4.180913322	-3.111869237	-0.991121073
C	2.103789160	-3.274104250	-0.380642029
C	2.150468166	-4.685043357	-0.471215036
H	3.111484239	-5.168805392	-0.661481049
C	1.003756074	-5.436073411	-0.321290024
H	1.042726079	-6.524961489	-0.383228029
C	-0.232799018	-4.802355365	-0.096928008
H	-1.132985085	-5.408595406	0.011163001
C	-0.304726023	-3.421662262	-0.019051001
C	0.868623065	-2.625681199	-0.150712012
C	-2.654600201	-3.388668261	0.463841035
H	-2.513631192	-4.092598311	1.300425101
H	-2.986781229	-3.953579299	-0.423395032
C	-3.687852282	-2.367034182	0.862143067
H	-4.618032351	-2.899677219	1.125815088
H	-3.342977256	-1.815738139	1.755459135
C	-5.063113387	-0.662564053	-0.107746008
H	-5.923315426	-1.254762095	0.248494019
H	-5.289299401	-0.311350024	-1.125615085
C	-4.874043372	0.526122040	0.807339064
H	-5.840621427	1.052875079	0.904167070
H	-4.572027348	0.199714015	1.819606139
C	-3.612845276	2.493088193	1.105468085
H	-3.203334243	2.139316162	2.069797158

H	-4.535180345	3.062824232	1.317351100
C	-2.631463202	3.427734264	0.448442034
H	-3.014296228	3.787206290	-0.521779040
H	-2.489475188	4.293890325	1.114278087
Ba	-1.548418119	0.074431006	-1.010427079
Cl	2.318295180	-0.401070031	2.677231202
Fe	2.052023158	-0.031990002	0.496235038
N	3.472877264	-1.264180095	-0.433285033
N	3.549411269	1.349748104	0.010081001
O	0.801154061	1.386917106	0.207042016
O	0.737120058	-1.320509103	-0.081362006
O	-1.442076109	-2.709246209	0.180964014
O	-3.915358298	-1.484915113	-0.218702017
O	-3.897047299	1.394678105	0.266316020
O	-1.400628105	2.743023211	0.260563020

1. C. J. Van Staveren, J. Van Eerden, F. C. J. M. Van Veggel, S. Harkema and D. N. Reinhoudt, *J. Am. Chem. Soc.*, 1988, **110**, 4994-5008.
2. APEX2 Version 2014.11-0, Bruker AXS, Inc.; Madison, WI 2014.
3. SAINT Version 8.34a, Bruker AXS, Inc.; Madison, WI 2013.
4. Sheldrick, G. M. SADABS, Version 2014/5, Bruker AXS, Inc.; Madison, WI 2014.
5. Sheldrick, G. M. SHELXTL, Version 2014/7, Bruker AXS, Inc.; Madison, WI 201.
International Tables for Crystallography 1992, Vol. C., Dordrecht: Kluwer Academic Publishers
6. A. Coletti, P. Galloni, A. Sartorel, V. Conte, B. Floris, *Catal. Today*, 2012, **192**, 44-55.
7. D.-F. Liu, X.-Q. Lü and R. Lu, *Transit. Met. Chem.*, 2014, **39**, 705-712.
8. M. Ferguson, N. Giri, X. Huang, D. Apperley, S. L. James, *Green Chem.*, 2014, **16**, 1374.
